# Magnon Straintronics in the 2D van der Waals Ferromagnet CrSBr from First-Principles

**DOI:** 10.1021/acs.nanolett.2c02863

**Published:** 2022-09-26

**Authors:** Dorye L. Esteras, Andrey Rybakov, Alberto M. Ruiz, José J. Baldoví

**Affiliations:** Instituto de Ciencia Molecular, Universitat de València, Catedrático José Beltrán 2, 46980 Paterna, Spain

**Keywords:** 2D materials, 2D magnetism, magnonics, straintronics, first-principles calculations

## Abstract

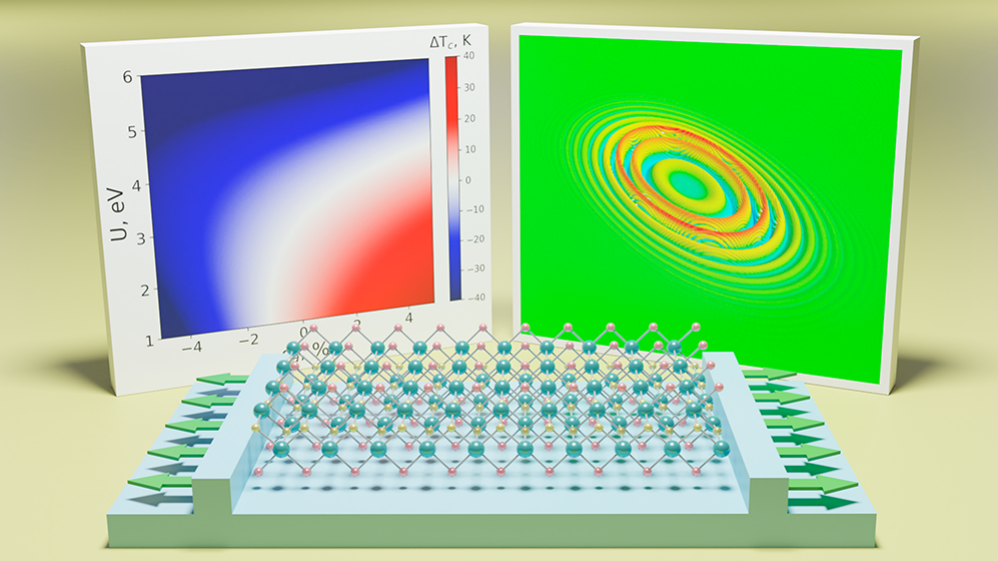

The recent isolation of two-dimensional (2D) magnets offers tantalizing opportunities for spintronics and magnonics at the limit of miniaturization. One of the key advantages of atomically thin materials is their outstanding deformation capacity, which provides an exciting avenue to control their properties by strain engineering. Herein, we investigate the magnetic properties, magnon dispersion, and spin dynamics of the air-stable 2D magnetic semiconductor CrSBr (*T*_C_ = 146 K) under mechanical strain using first-principles calculations. Our results provide a deep microscopic analysis of the competing interactions that stabilize the long-range ferromagnetic order in the monolayer. We showcase that the magnon dynamics of CrSBr can be modified selectively along the two main crystallographic directions as a function of applied strain, probing the potential of this quasi-1D electronic system for magnon straintronics applications. Moreover, we predict a strain-driven enhancement of *T*_C_ by ∼30%, allowing the propagation of spin waves at higher temperatures.

Magnonics is an emerging research field within nanomagnetism and nanoscience that investigates the transmission, storage, and processing of information using spin waves (SWs) as an alternative to conventional electronics.^1−3^ The use of SWs, whose quanta are referred to as magnons, instead of transport of electric charges offers unique and compelling opportunities such as extremely low power consumption, shorter wavelengths, nanoscale devices, tunable spectrum, and wave-based computing concepts, to name a few.^4−7^ Besides unlocking new horizons in fundamental physics, the recent discovery of long-range magnetic order in atomically thin crystals provides an unprecedented platform for magnonics at the limit of miniaturization.^8,9^ Among the family of 2D magnetic crystals, layered antiferromagnets such as CrI_3_ or CrSBr are particularly interesting because their magnetic properties can be controlled by electrostatic doping,^10^ electric fields,^11^ or strain^12^ and can hold long-lived magnons in the GHz to THz range,^13,14^ and their van der Waals (vdW) nature ensures an easy transfer onto the surface of other nanomaterials in order to improve the device performance.^15^

Strain engineering has been shown to be a powerful tool to tune the electronic and magnetic properties of 2D materials due to their larger elasticity compared to bulk crystals.^16−22^ In intrinsic 2D magnets, strain has been able to induce switching between antiferromagnetic (AFM) and ferromagnetic (FM) interlayer coupling and successfully modulate magnetic exchange interactions, magnetic anisotropy, and Curie temperature (*T*_C_) down to the single-layer.^23−25^ In the context of magnonics, a new branch called magnon straintronics has recently been proposed and experimentally implemented, offering a promising route to generate SWs.^26−28^ However, the effect of strain on the SW dynamics of 2D materials is still unexplored—even from a theoretical point of view—and deserves urgent attention, owing to its potential to develop a new generation of 2D magnonic devices.

Motivated by these burgeoning developments, herein, we focus on the 2D air-stable semiconductor CrSBr, which is formed by ferromagnetic layers (*T*_C_ = 146 K) with antiferromagnetic interlayer coupling.^29,30^ Interestingly, the material presents a quasi-1D electronic structure entangled with its magnetic structure, resulting in an ideal candidate to control the magnetic properties by applying uniaxial deformations of the lattice.^31^ With the aim of investigating the effects of tensile and compressive strain on the SW dynamics of CrSBr at the 2D limit, we have implemented an efficient first-principles methodology that combines density functional theory (DFT), a derived tight-binding Hamiltonian, spin wave theory, and atomistic simulations. This allows us to provide a detailed microscopic analysis of magnetic exchange and thus SW propagation. Interestingly, we show that magnons in single-layer CrSBr can be modified selectively along the two main crystallographic directions as a function of the applied strain, which paves the way to the use of this 2D semiconductor for magnon straintronics applications.

Bulk CrSBr crystallizes in the orthorhombic *Pmmm* space group with lattice parameters *a* = 3.50 Å, *b* = 4.76 Å, and *c* = 7.96 Å, exhibiting a vdW layered structure.^32^ This allows the crystal to be exfoliated down to single-layer flakes.^30^ In each layer, the Cr atoms are embedded in a distorted octahedral coordination environment and are connected to their nearest-neighbor Cr atoms by sulfur and bromine atoms along the *a* axis, whereas they are connected only by sulfur atoms along *b* and *c* axes, resulting in decoupled quasi-1D chains as proved by conductivity measurements.^33^

In the CrSBr monolayer, magnetic exchange interactions between Cr atoms can be modeled considering up to third nearest-neighbors superexchange mechanisms through the p orbitals of Br and S ligands.^34^ Thus, there are mainly three magnetic exchange interactions represented by *J*_1_, *J*_2_, and *J*_3_, where *J*_1_ accounts for the interaction between Cr–Br–Cr (89°) and Cr–S–Cr (95°) atoms along the *a* direction; *J*_2_ coupling between Cr atoms from different “sublayers” along *c* (97°); and *J*_3_ the interaction between Cr atoms mediated by softer S bridges along the *b* axis (160°) (Figure 1). The intricate relation between magnetism and crystal lattice has been recently investigated by synchrotron X-ray diffraction measurements, evidencing that the thermal evolution of the lattice parameters follows opposing trends when cooling down the crystal.^31^ While *a* tends to elongate due to an unconventional expansion when the temperature decreases from 260 K to the so-called spin-freezing temperature T* ≈ 40 K, both *b* and *c* lattice parameters become shorter. Intriguingly, these trends lead to a progressive enhancement of *J*_1_, *J*_2_, and *J*_3_.^35^

**Figure 1:**
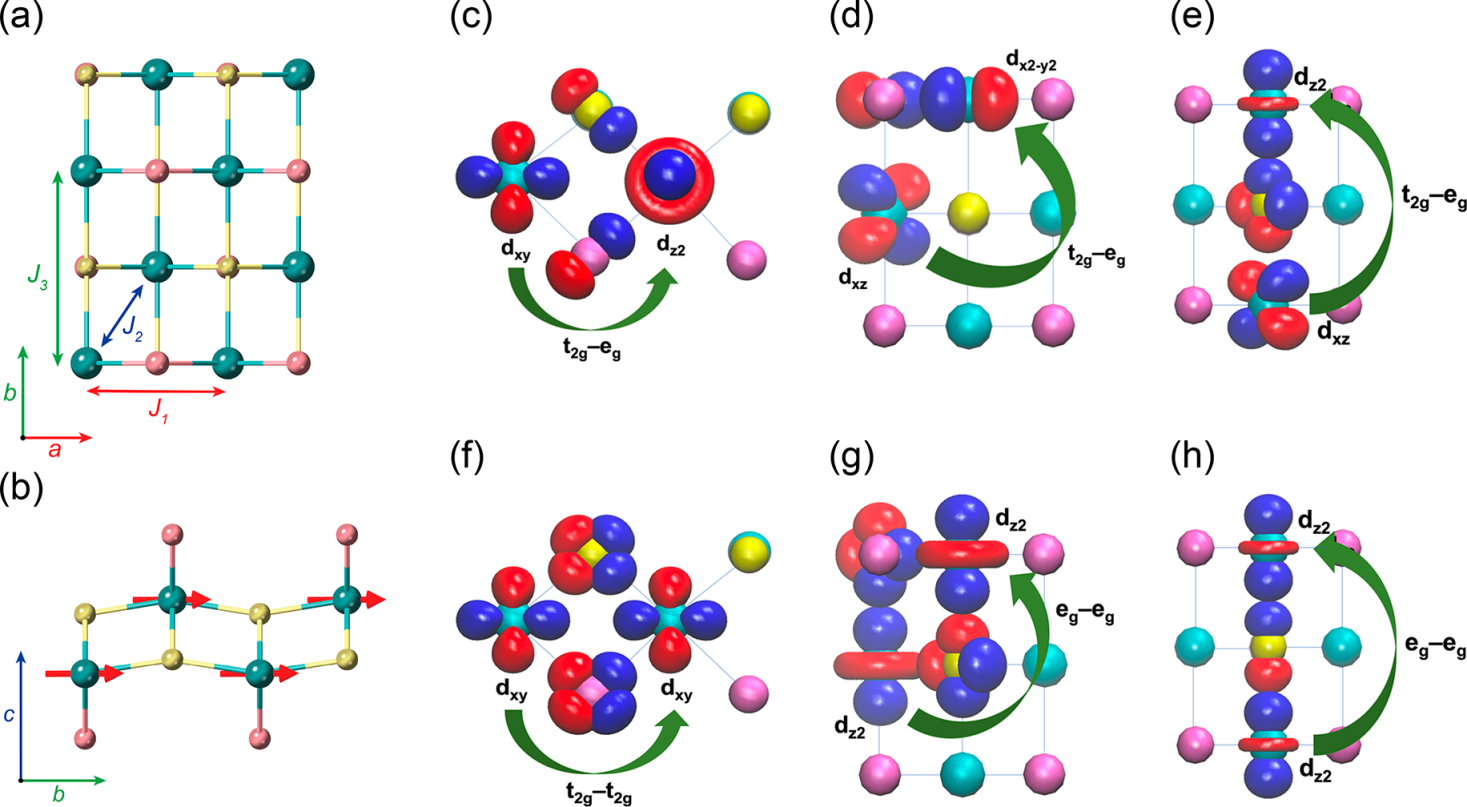
(a) Top view of the crystal structure of a single CrSBr layer. Cyan, yellow, and pink balls represent chromium, sulfur, and bromine atoms, respectively. *J*_1_, *J*_2_, and *J*_3_ magnetic exchange interactions for first, second, and third neighbors are represented by arrows that connect the Cr atoms. (b) Side view of the same CrSBr structure showing the spin orientation along *b*. Calculated maximally localized Wannier orbitals. Green arrows illustrate the most relevant magnetic superexchange channels, namely, t_2g_–e_g_ (FM), t_2g_–t_2g_ (AFM), and e_g_–e_g_ (AFM) for *J*_1_ (c, f), *J*_2_ (d, g), and *J*_3_ (e, h).

In order to understand the electronic and magnetic structure of single-layer CrSBr, we perform first-principles calculations including spin–orbit coupling (SOC) (see the Methods section). The DFT+U electronic band structure and density of states (DOS) are plotted in Figure S1. One can observe the presence of highly dispersive conduction bands along the *X*–*S* and *Y*–Γ directions, which are characteristic of this 2D semiconductor with quasi-1D electronic properties.^33^ The orbital-resolved DOS (Figures S1–S4) illustrates the key contribution of the d orbitals of Cr and p orbitals of S and Br atoms around the Fermi level. The octahedral crystal field around the Cr atoms splits the d orbitals into two sets of energy levels, namely, t_2g_ (d_*xy*_, d_*xz*_, and d_*yz*_) and e_g_ (d_*z*^2^_ and d_*x*^2^–*y*^2^_) (Figure S2).^36^ Accordingly, each Cr atom carries a net magnetic moment of 3.03 μ_B_, which agrees well with *S* = 3/2 for Cr^3+^. S and Br atoms are slightly spin polarized with −0.27 μ_B_ and −0.08 μ_B_, respectively. The most stable magnetic configuration is achieved for FM ordering with in-plane magnetization along the *b* axis (Figure 1b), in agreement with experiments.^37−39^

To determine the magnetic exchange interactions, we derive a tight-binding Hamiltonian expressed in the basis of maximally localized Wannier functions (MLWFs)^40^ that permits the use of Green’s function approach in the TB2J package.^41^ This method treats the local spin rotation as a perturbation and is an efficient route that circumvents the main limitations of total energy mapping analysis, i.e., (i) the requirement of a number of magnetic configurations at least N+1 than the parameters of the Hamiltonian, (ii) the difficult convergence for some metastable configurations, or (iii) the use of large supercells. The obtained magnetic exchange parameters for *U* = 3 eV (isotropic *J*_1_ = 3.54 meV, *J*_2_ = 3.08 meV, and *J*_3_ = 4.15 meV; anisotropic *J*_1,*yy*_ = 0.015 meV, *J*_2,*yy*_ = 0.004 meV, and *J*_3,*yy*_ = 0.001 meV) are close to the ones recently estimated from neutron diffraction measurements in bulk CrSBr^42^ and agree well with theoretical calculations for the monolayer reported in the literature (see Table S1).^43,44^ We also provide the evolution of the isotropic parameters as a function of Hubbard *U* (Table S2) accompanied by the orbital decomposed magnetic exchange channels (Table S3 and Figures S6–S16), which are graphically represented in Figure 1c–h. One can observe that almost in the entire range of *U*, the magnetic exchange parameters are >0 due to the dominant t_2g_–e_g_ (FM) pathway; however, while *J*_1_ increases with *U*, *J*_2_ and *J*_3_ decrease. This different evolution comes from the particular orbitals involved in the FM and AFM superexchange mechanisms for each direction. In the case of *J*_1_, the AFM t_2g_–t_2g_ hopping takes place between the d_*xy*_ of the two Cr atoms and p_*x*_ and p_*y*_ orbitals of the ligands contained in the *ac* plane. It can be observed that an enhancement of Coulomb interactions drastically limits the AFM t_2g_–t_2g_ pathway, which is relevant along *a*. By contrast, the dominant AFM exchange for *J*_2_ and *J*_3_ arises from e_g_-like orbitals, mainly d_*z*^2^_ that points along *b*, whose occupations increase with *U* (Figure S17). This favors the AFM d_*z*^2^_–p_*z*_–d_*z*^2^_ superexchange pathway along the *b* direction, whereas the t_2g_–e_g_ FM mechanism is slightly improved, leading to *J*_3_ < 0 at *U* ≈ 6 eV.

Then, we investigate the strain-dependent evolution of magnetic exchange parameters by applying two types of uniaxial strain (along *a* and *b* axes). The lattice parameters were varied up to 5% of compression and elongation. In parallel, Hubbard *U* was evaluated in the range 1–6 eV to simulate the effect of different screening scenarios. We achieved a high-density grid of outputs by applying the least-squares method using eq 1, which successfully reproduces the calculated results and can be used to interpolate the intermediate points:
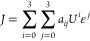
1where *J* are the magnetic exchange interactions up to three nearest neighbors, *a*_*i*,*j*_ are the fitting coefficients, *U* is the on-site Hubbard parameter, ε is % strain, and *i* and *j* are the powers of the fit.

Figure 2 shows a 3D surface plot of each *J* dependence with respect to ε and *U*. Due to the asymmetric structure of CrSBr, one can observe that each exchange interaction presents an independent evolution depending on the crystallographic direction along which strain is applied. As described above, *J*_1_ and *J*_3_ connect Cr atoms along the *a* and *b* axes, respectively. This causes the *J*_1_ parameter to be strongly influenced by uniaxial strain in *a*, whereas *J*_3_ changes notably by applying strain along *b*. On the other hand, *J*_2_ interaction takes place between both axes (Figure 1a) and thus is affected by ε in both lattice directions. In Tables S4 and S5, we present a complete orbital-resolved analysis of exchange parameters as a function of strain. Application of strain in *a* can be used to precisely modify the influence of the t_2g_–t_2g_ mechanism (Figure 1f), which is reduced by minimizing the overlap between the in-plane *d*_*xy*_, *p*_*x*_, and *p*_*y*_ orbitals (Tables S6–S8). Thus, an expansion of the *a* lattice parameter results in a decrease of the AFM contribution of *J*_1_, favoring FM interactions (Figures S19–S22). The opposite effect is encountered when applying strain along *b*, which modifies the t_2g_–e_g_ channels that include orbitals with the *z* component (Tables S14, S15, S19, and S20). This mainly affects second and third neighbors, resulting in an enhancement of ferromagnetism when the system is compressed along *b* (Figures S35–S40).

**Figure 2:**
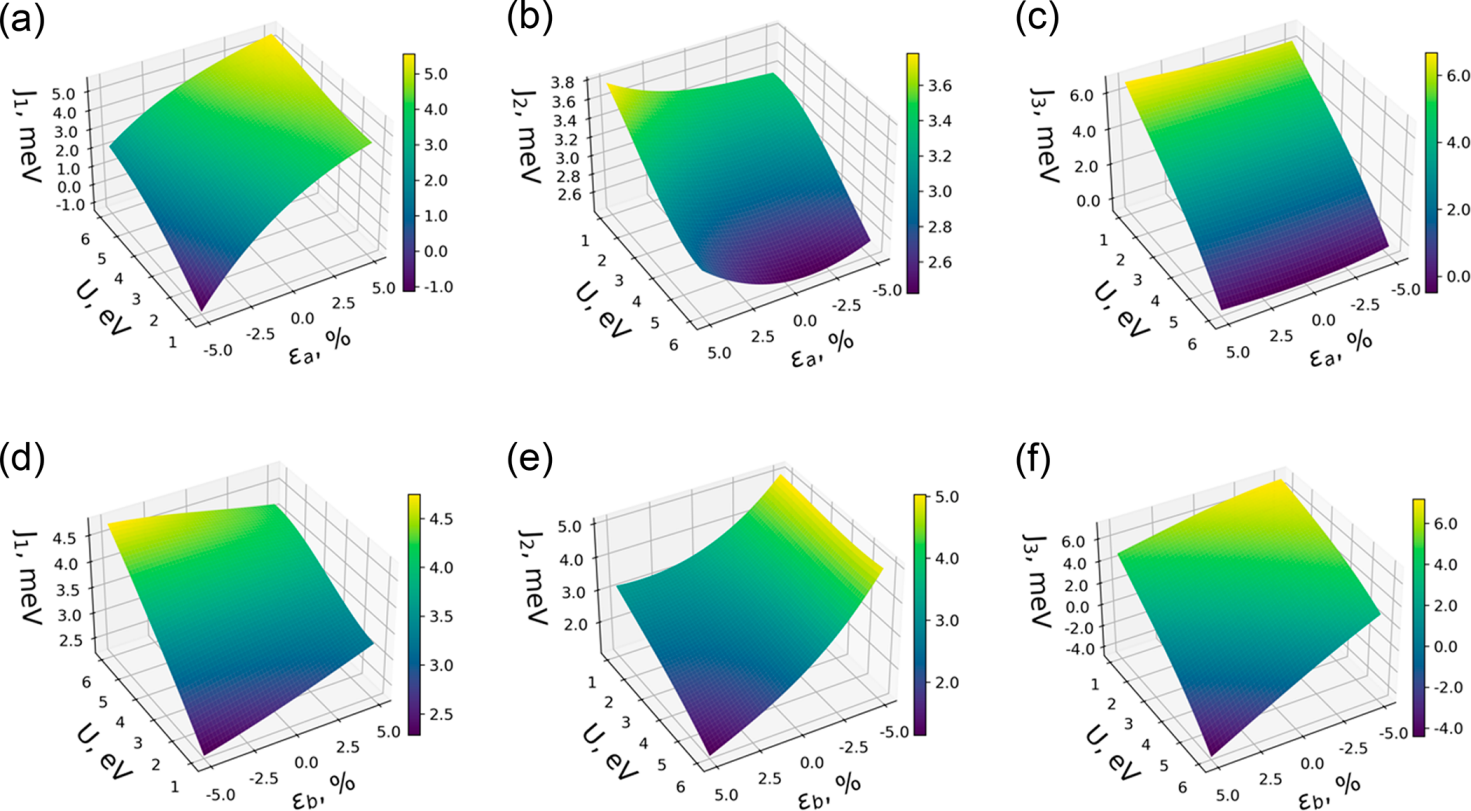
High-density 3D surface plots of isotropic exchange parameters (*J*_1_, *J*_2_, *J*_3_) as a function of strain (ε) and Hubbard *U* for the CrSBr monolayer: (a–c) uniaxial strain in the *a* axis; (d–f) uniaxial strain in the *b* axis.

From magnetic exchange, the magnon dispersion is obtained via a Holstein–Primakoff^45^ transformation in the framework of linear spin-wave theory (LSWT), considering the bosonic operator terms up to second order. The resulting spin-wave Hamiltonian in reciprocal space is represented in eqs 2 and 3:
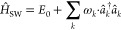
2
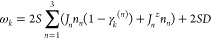
3where *J*_*n*_ (*J*_*n*_^*z*^) are isotropic (anisotropic) exchange parameters, *n*_*n*_ the coordination numbers, and γ_*k*_^(*n*)^ the structural factors of the different neighbors.

The effects of uniaxial strain on the magnon dispersion of the CrSBr monolayer are reported in Figure 3. As a result of the two magnetic Cr atoms in the unit cell, we can observe both acoustic and optical modes, which are degenerated along *X*–*S* and *S*–*Y* directions when a symmetric anisotropic Hamiltonian is considered. This degeneracy could be lifted through antisymmetric Dzyaloshinskii–Moriya interaction (DMI) induced by proximity effects, since DMI is negligible in CrSBr itself.^46^ By contrast, highly dispersive branches appear in the Γ–*X* and Γ–*Y* directions which are related to the two main reciprocal axes (*a* and *b* axis direction in real space, respectively). This leads to a selective control of the magnon dispersion with strain as observed in Figure 3a (uniaxial *a*) and Figure 3b (uniaxial *b*), having a strong influence in the anisotropy gap at Γ,^47^ which plays a crucial role in establishing long-range magnetic ordering at a finite temperature, thus removing the Mermin–Wagner theorem restriction.^48^ The full evolution of the magnon dispersion as a function of strain and *U* is presented in Figures S44 and S45.

**Figure 3:**
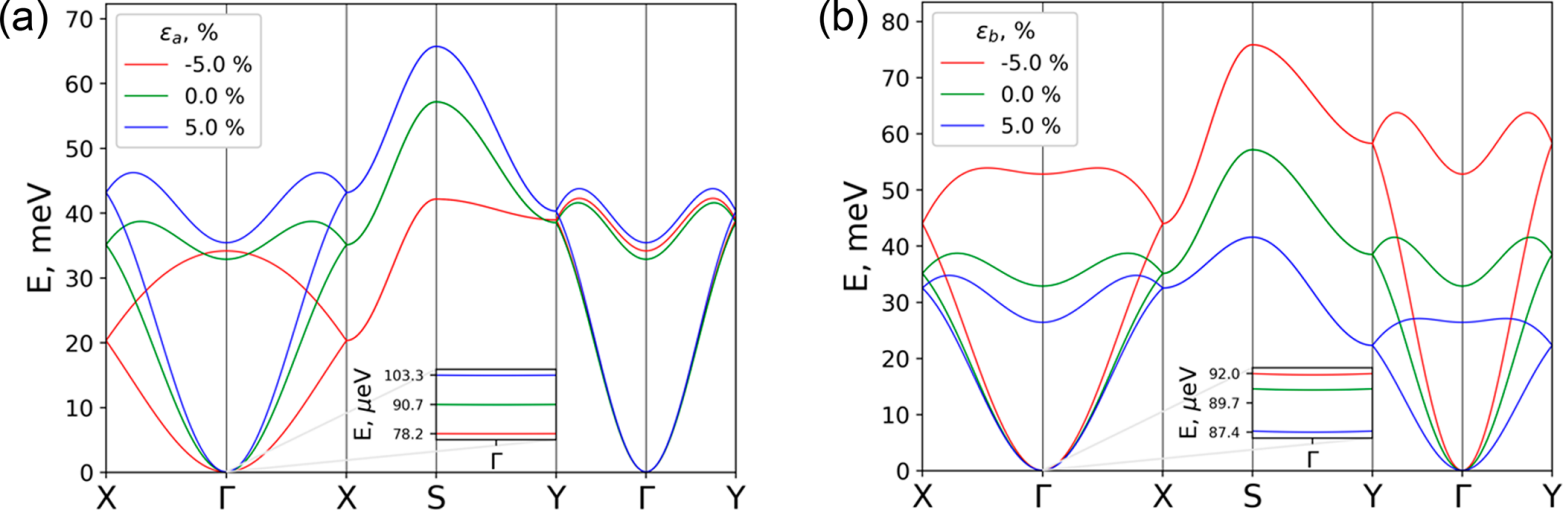
Magnon dispersion of the CrSBr monolayer (*U* = 3 eV): (a) uniaxial strain in the *a* axis and (b) uniaxial strain in the *b* axis. Inset: anisotropic gap at the Γ point.

Figure 4 presents the evolution of *T*_C_ calculated from renormalized spin-wave theory (RSWT) (panels a, c, d, and f) and the gap at the Γ point in the spin wave spectrum (panels b and e) as a function of ε and *U*. The high-density 3D maps show a dramatic effect of uniaxial strain along *a* on the anisotropy gap, which increases (decreases) up to 14% (14%) with positive (negative) 5% strain for *U* = 3 eV (Figures 3a and 4b). On the contrary, when strain is applied along *b*, the gap exhibits a moderate decrease with positive strain, i.e., only by 4% under a 5% strain (Figures 3b and 4e). This can be attributed to the unbalanced effect of both types of strain on the distorted octahedral coordination environment, which involves four ligands directly affected by strain along *a* (*ac* plane) versus two sulfur atoms along the *b* direction.

**Figure 4:**
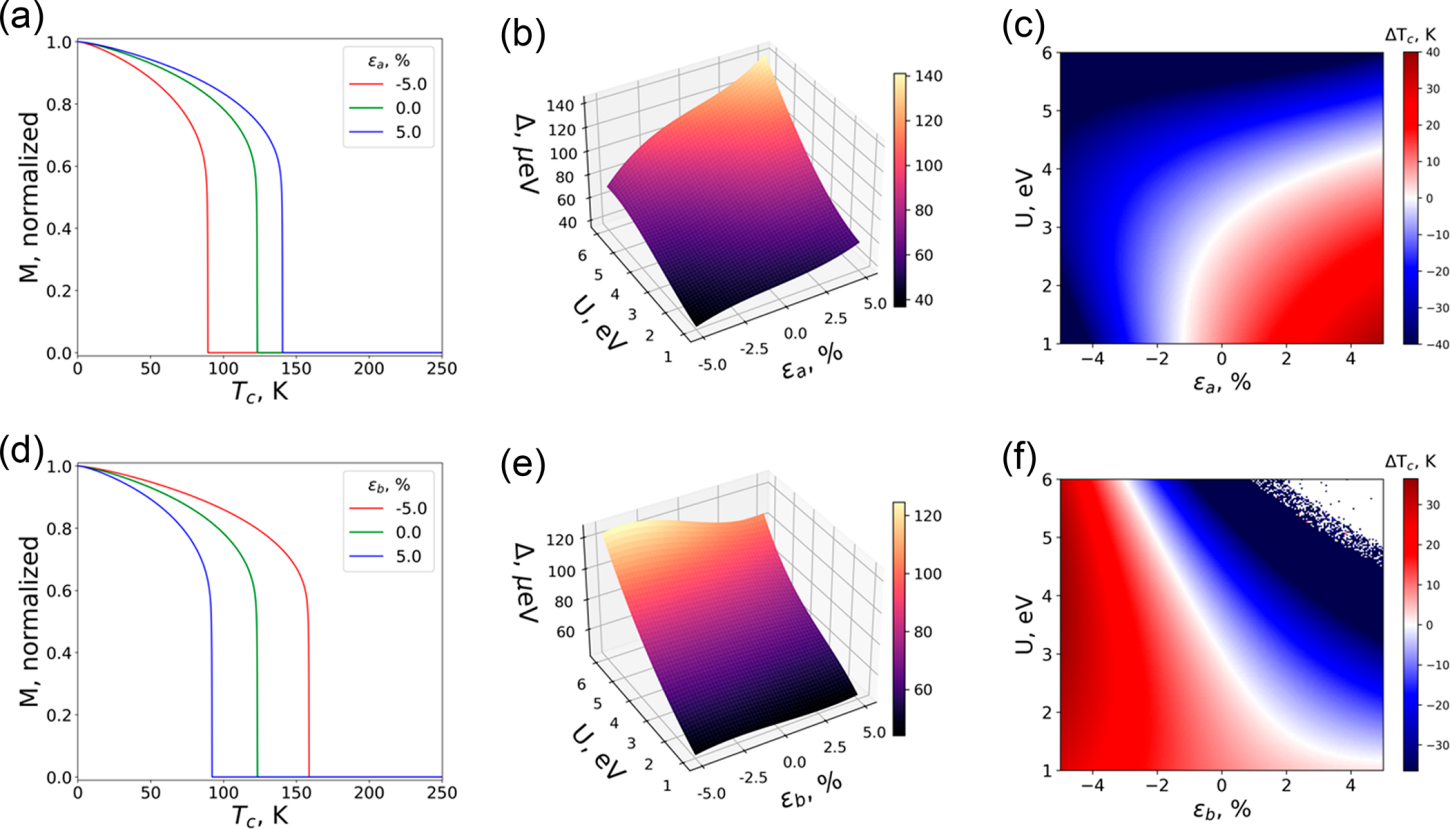
(a, d) Temperature dependence of the Cr magnetic moment. (b, e) Gap at the Γ point in the magnon dispersion. (c, f) Curie temperature map. (a–c) Uniaxial strain in the *a* axis. (d–f) Uniaxial strain in the *b* axis. Relative point *T*_c_ = 122 K corresponds to *U* = 3 eV and an unstrained sample.

Regarding the dependence of the calculated *T*_C_ on the anisotropy gap and the isotropic magnetic exchange interactions, one can find a clear competition between them when applying uniaxial deformation along the *a* axis (Figure 4b,c). This is more evident at higher *U* values where *T*_C_ drops dramatically despite the improvement of the anisotropy gap and *J*_1_. The reason behind that is the growth of the population of the e_g_ orbitals (Figure S17) that activate the AFM exchange channel between the d_*z*^2^_–p_*z*_–d_*z*^2^_ orbitals parallel to the *b* axis. Thus, *T*_C_ follows the gap evolution for *U* < 4 eV, but at larger *U*, *J*_3_ becomes negative and starts to play a crucial role in destabilizing the FM configuration. On the other hand, our results show a good correlation between the gap and the critical temperature of CrSBr for compressive/tensile strain applied in *b*. This is because, in this case, *J*_3_ is directly controlled by the proposed experiment. The compression of the *b* parameter yields a poorer effect of the e_g_–e_g_ AFM channel, thus making the FM configuration, mainly supported by t_2g_–e_g_ interactions, more stable. This results in a ∼30% enhancement of *T*_C_, predicting an upper limit for *T*_C_ of 158 K for *U* = 3 eV and ε = −5% along *b* (Figure 4d). In the extreme region of *U* ≈ 6 eV, for ε ≈ 2%, and *U* ≈ 5 eV, for ε ≈ 3.5%, the system becomes antiferromagnetic along *b*, and *T*_C_ vanishes (blank region in Figure 4f and Figure S43).^49^ This behavior can be rationalized by the Goodenough–Kanamori rules^50^ since the superexchange pathway for *J*_3_ becomes AFM when the angle Cr–S–Cr approaches 180° due to stretching of the *b* lattice parameter.

Finally, we evaluate the magnon dynamics by atomistic simulations based on the Landau–Lifshitz–Gilbert (LLG) equation:^51,52^
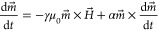
4where *m⃗* is the normalized magnetic moment of Cr atoms, *H⃗* is the effective exchange field, μ_0_ is the permeability of a vacuum, γ is the gyromagnetic ratio, and α is the Gilbert damping parameter that we set to a typical value of 0.01 for Cr-based 2D magnets.^53,54^ Our calculations start by perturbing the initial FM state with an oscillating magnetic field in a narrow region at the center of the sample for an ultrashort period of time (1 ps) with the objective of generating SWs. Then, the SWs propagate as graphically shown by selected snapshots from our real-time real-space spin dynamics simulations (see Figures S46 and S47).

The group velocity of SW propagation (*v*) is evaluated along the *a* and *b* crystallographic directions as a function of ε and *U* (see Figure 5 and Figure S48). One can observe several abrupt changes (e.g., at *v*_*a*_ = 2.6 × 10^3^, 3.3 × 10^3^, 3.7 × 10^3^, and 4.2 × 10^3^ m/s for ε along *a*) that can be attributed to the deactivation of higher-frequency magnon modes when mechanically modifying the lattice coordinates. This effect can be corrected by choosing an optimal magnetic field frequency for each value of strain and *U* (Figures S49–S55). According to our dynamic simulations, environmental screening has an opposite effect on *v* for *a* and *b* directions for both types of applied strain. In the case of *b*, the group velocity decreases with larger values of *U*. By contrast, higher Coulomb screening induces faster SWs along *a*. Strain mainly affects *v* in the same direction as it is applied. Thus, for uniaxial strain along *a*, changes in *v*_*b*_ are small—although they are more pronounced for larger values of *U* (see Figure S48)—whereas *v*_*a*_ can be significantly modified in the boundaries from −60% to 20% (see Figure 5a).

**Figure 5:**
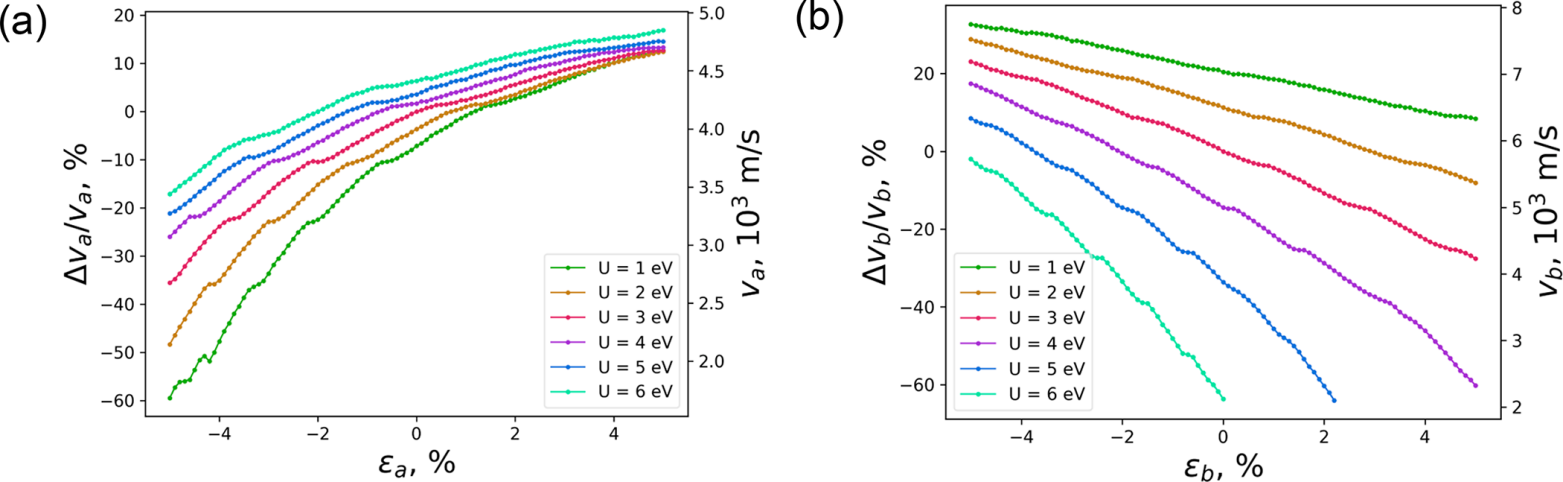
Group velocity in the direction of the *a* axis for uniaxial strain along *a* (a) and the *b* axis for uniaxial strain along *b* (b). The reference points are chosen to be at ε = 0% and *U* = 3 eV.

Our results show that strain-engineered tuning of group velocity is directly linked with changes in *J* values. This is also evident when inspecting the slope of the Γ–*X* and Γ–*Y* paths in the magnon dispersion, which correspond to *a* and *b*, respectively. Comparing Figures 3 and 5, it is straightforward to realize that Γ–*Y* has a larger slope at 0% strain, which is translated into a faster *v*_*b*_ = 5.8 × 10^3^ m/s with respect to *v*_*a*_ = 4.3 × 10^3^ m/s according to the LLG dynamics simulations. Regarding the application of mechanical strain, we can see that the Γ–*X* slope is strongly tuned by uniaxial strain along *a*, which results in pronounced changes in *v*_*a*_, whereas the Γ–*Y* path is controlled by uniaxial strain in *b*. This yields faster SWs along the Cr–S–Cr 1D-chains. Overall, we can observe that along *a* (*b*), the SW propagation is mostly influenced by *J*_1_ (*J*_3_), which can be microscopically controlled by uniaxial strain along those directions, with *J*_2_ playing a minor role for both of them.

Finally, we have seen that the microscopic mechanisms that are responsible for the spin dynamics can be tuned by varying Coulomb interactions. In the 2D limit, dielectric properties are extremely sensitive to the environment. Thus, embedded layers, substrates, capping materials, and encapsulation techniques do an effective job quenching electronic interactions.^55,56^ Substrates such as sapphire films or transition metal dichalcogenides, with a high dielectric constant, are the most effective to decrease Coulombic interactions.^57^ Nanomaterials like hBN or graphene can provide an efficient interface to reduce *U* due to their flat nature, even allowing the stacking of more layers to produce a higher screening.^58,59^ According to our predictions, this will increase the group velocity, reaching the best performance for uniaxial compressive strain along *b*.

In summary, we have investigated the magnetic properties, magnon dispersion, and spin dynamics of the air-stable 2D magnetic semiconductor CrSBr under uniaxial strain and Coulomb screening from first-principles. We provide a detailed microscopic understanding of the competing t_2g_–e_g_, t_2g_–t_2g_, and e_g_–e_g_, unveiling the magnetic exchange channels that stabilize ferromagnetism in the monolayer and shed light on the rational exploitation of magnon straintronics in this intriguing 2D material. Our calculations demonstrate that the magnetic properties and thus the magnon dynamics of CrSBr can be selectively tuned along the two main crystallographic directions (*a* and *b*) as a function of applied strain and environmental screening. Furthermore, we predict a strain-driven enhancement of *T*_C_ ≈ 30%, allowing the propagation of spin waves at higher temperatures.

## Methods

To describe the electronic structure of CrSBr, we performed *ab initio* calculations using the Quantum ESPRESSO package.^60^ In order to describe the strong correlation of the electrons present in CrSBr, we adopted a DFT+U approach, where *U* is the on-site Coulomb repulsion, using the simplified version proposed by Dudarev et al.^61^ A value of *U* = 3 eV was taken from the bibliography to optimize the structures. Hubbard *U* determined by orthogonalized atomic projectors was used to correctly describe the magnetic exchange under strain conditions. The generalized gradient approximation (GGA) and the Perdew–Burke–Ernzerhof (PBE) functional were used to describe the exchange-correlation energy.^62^ We selected standard solid-state US pseudopotentials from the QuantumEspresso database. The electronic wave functions were expanded with well-converged kinetic energy cut-offs for the wave functions and charge density. All the structures were fully optimized using the Broyden–Fletcher–Goldfarb–Shanno (BFGS) algorithm^63^ until the forces on each atom were smaller than 1 × 10^–3^ Ry/au, and the energy difference between two consecutive relaxation steps was less than 1 × 10^–4^ Ry. The Brillouin zone was sampled by a fine Γ-centered 8 × 8 × 1 *k*-point Monkhorst–Pack mesh for all calculations.^64^ Wannier90 calculations were performed ensuring a correct fit to the electronic band structure and spreads.^65^ Non-collinear TB2J calculations were performed with a 20 × 20 × 1 supercell and carefully selected limits of the exchange integral. Self-consistent Curie temperature calculations were performed using a *k*-point sampling of 300 × 300 × 1 for BZ integration. LLG-driven dynamics were solved with an integration time step of 10 fs. High-frequency magnons were induced by applying a driving magnetic field of the frequency 1 THz and magnitude 0.13 T oscillating along the *c* axis for a period of 1 ps. Simulations were performed for a 251 × 201 × 1 supercell with free boundary conditions and a Gilbert damping parameter of 0.01. The single ion anisotropy constant was obtained from previously reported calculations on the CrSBr monolayer.^36^

## Supporting Information Available

Electronic structure; exchange Hamiltonian; orbital-resolved analysis; hopping integrals; phase diagram of the exchange Hamiltonian; magnon dispersion and Curie temperature; LLG-driven atomistic spin dynamic simulations; and evolution of self-consistent Hubbard *U* with strain (PDF)
