# Supplementary material for: Magnon Straintronics in the 2D van der Waals Ferromagnet CrSBr from First-Principles
Source: Nano Lett. 2022 Sep 26;22(21):8771–8. doi: 10.1021/acs.nanolett.2c02863 (PMC9650781; doi:10.1021/acs.nanolett.2c02863)
Supplement: Supplementary file 1 — nl2c02863_si_001.pdf [file nl2c02863_si_001.pdf]

Supporting information

Magnon straintronics in the 2D van der Waals ferromagnet CrSBr from first-principles

Dorye L. Esteras, [‡,†] Andrey Rybakov, [‡,†] Alberto M. Ruiz,[†] José J. Baldoví[*,†]

[†]Instituto de Ciencia Molecular, Universitat de València, Catedrático José Beltrán 2, 46980 Paterna, Spain.

E-mail: j.jaime.baldovi@uv.es

Table of Contents

1. Band structure and orbital resolved density of states 2

2. Exchange Hamiltonian 6

3. Orbital-resolved analysis 8

3.1 Isotropic exchange vs Hubbard U (ε = 0%) 8
3.2 Occupations vs Hubbard U (ε = 0%) 20
3.3 Isotropic exchange vs strain applied along *a* (U = 3 eV) 21
3.4 Magnetic moment per atom vs strain applied along *a* (U = 3 eV) 32
3.5 Isotropic exchange vs strain applied along *b* (U = 3 eV) 33
3.6 Magnetic moment per atom vs strain applied along *b* (U = 3 eV) 44

4. Hopping integrals 45

5. Phase diagram of the exchange Hamiltonian. 63

6. Magnon dispersion and Curie temperature 64

7. LLG-driven atomistic spin dynamic simulation 67

7.1 Snapshots of spin dynamics simulations 68
7.2 Group velocity 69
7.3 Group velocity with frequency/time correction 70
7.4 Correction of frequency 72
7.5 Group velocities vs momentum within Brillouin zone 73

8. Evolution of self-consistent Hubbard U with strain 78

1. Band structure and orbital resolved density of states

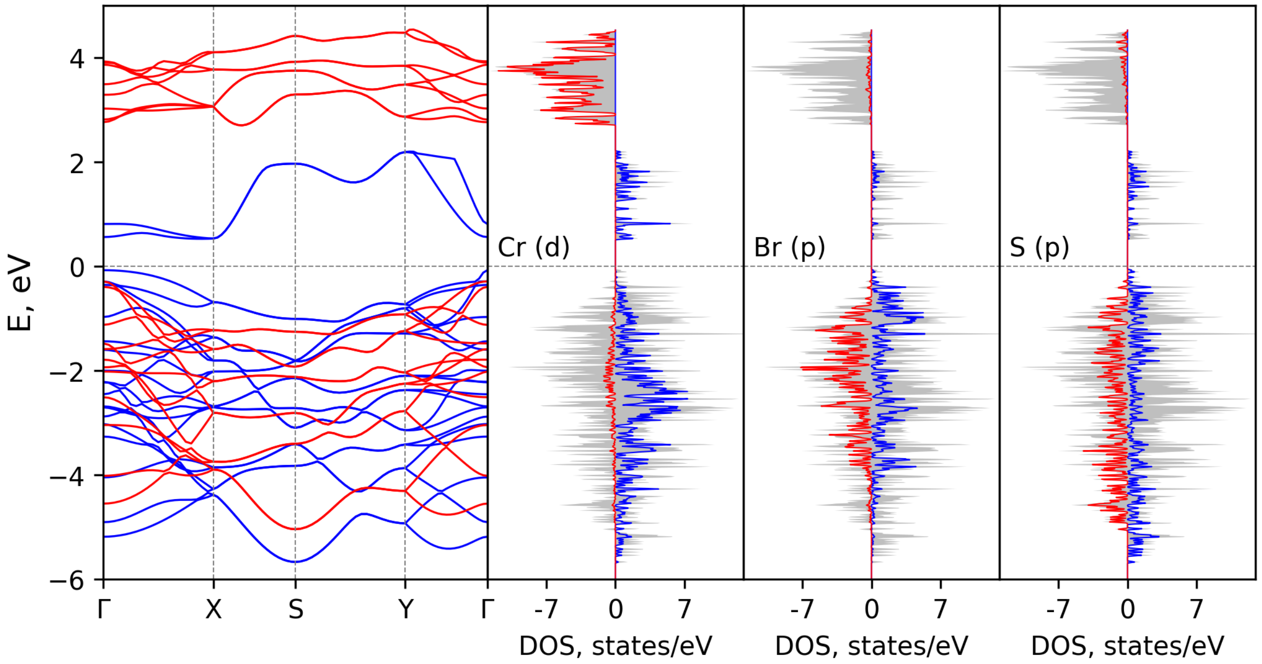

Figure S1. Electronic band structure (left) and orbital-resolved density of states (right) of CrSBr monolayer for U = 3 eV. Spin up (blue), spin down (red) and total DOS (grey background).

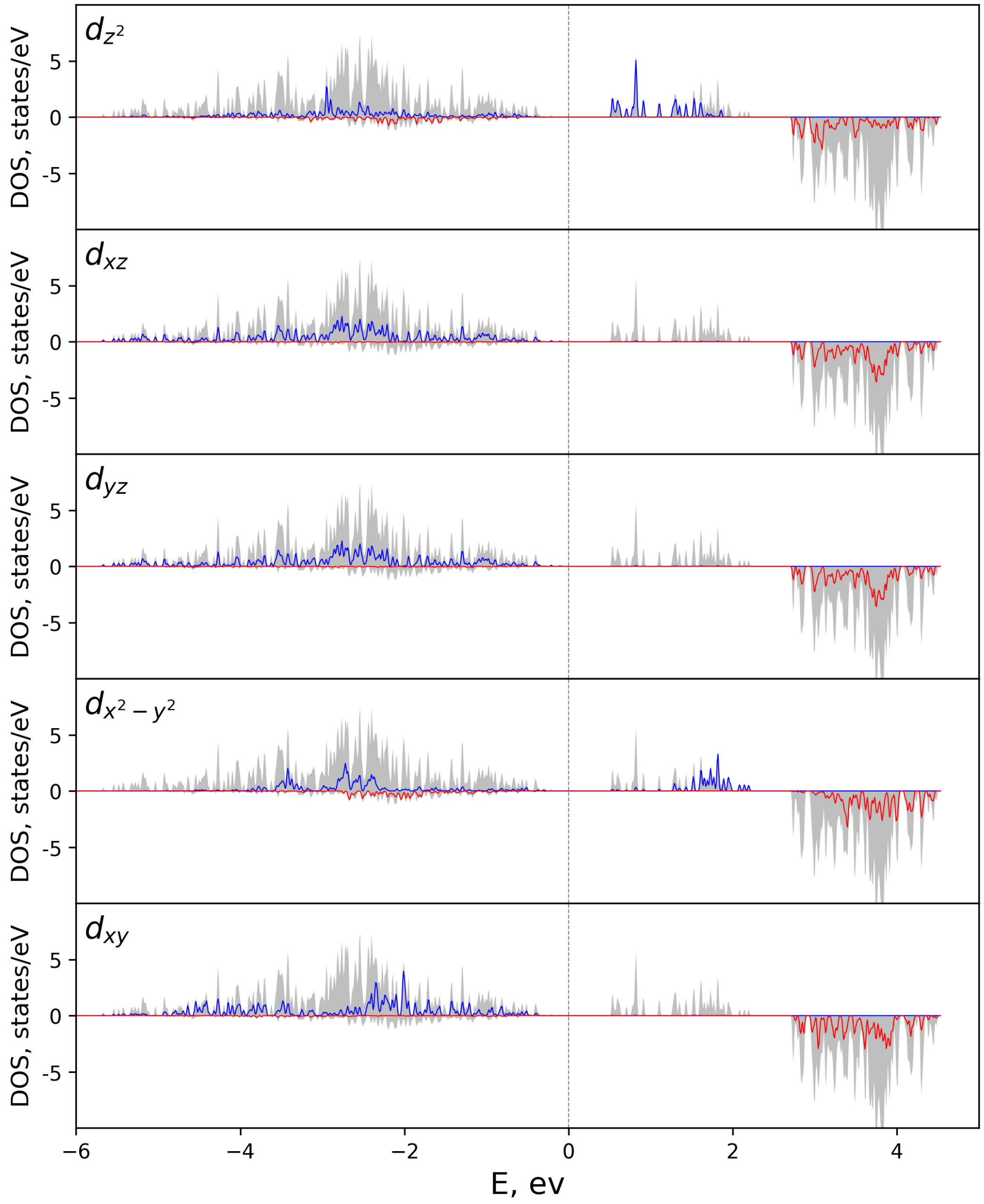

Figure S2. Orbitally-resolved density of states for Cr atoms of CrSBr monolayer for U = 3 eV. Spin up (blue), spin down (red) and total DOS (grey background) of Cr atoms.

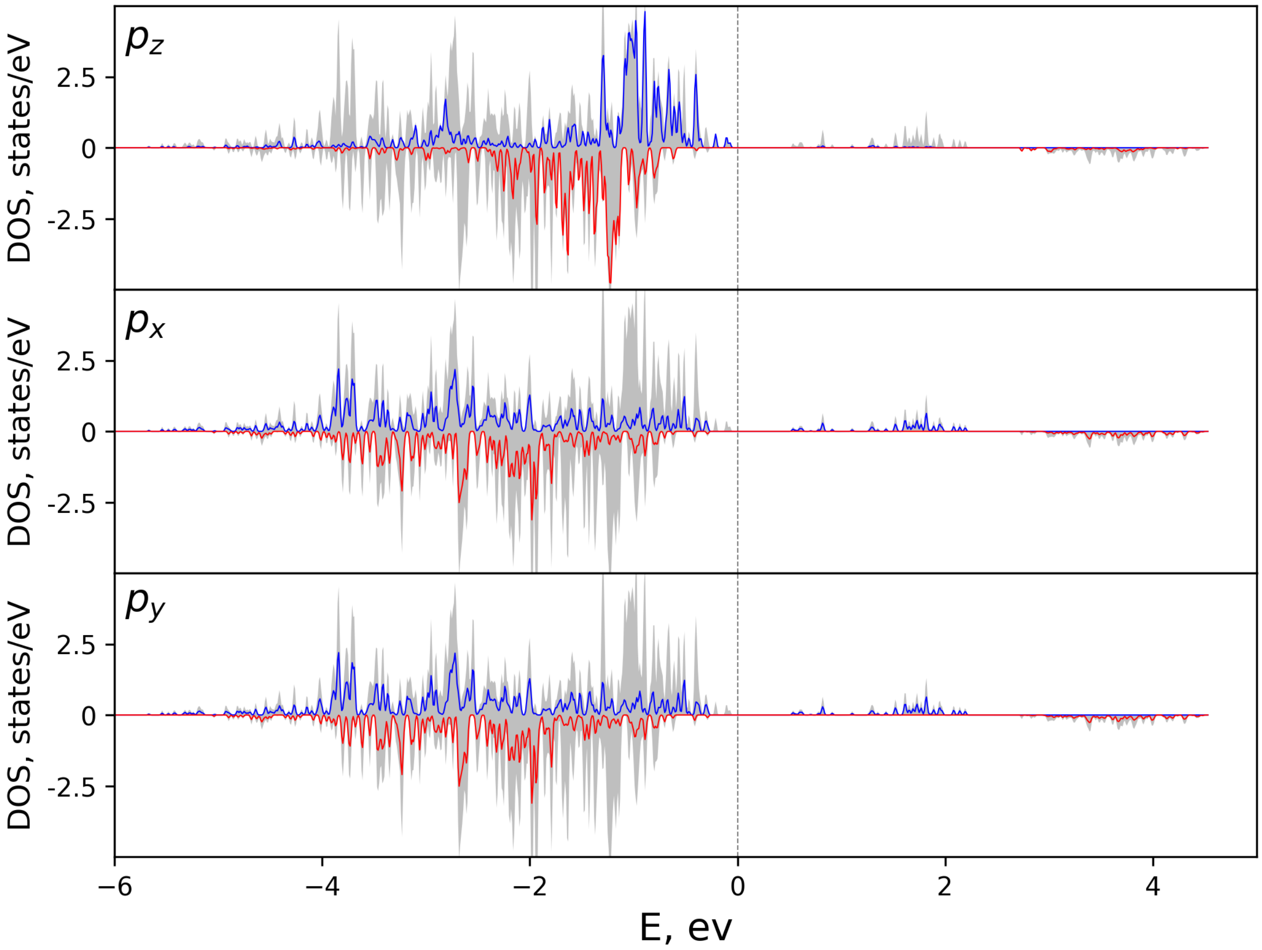

Figure S3. Orbitally-resolved density of states for Br atoms of CrSBr monolayer for U = 3 eV. Spin up (blue), spin down (red) and total DOS (grey background) of Br atoms.

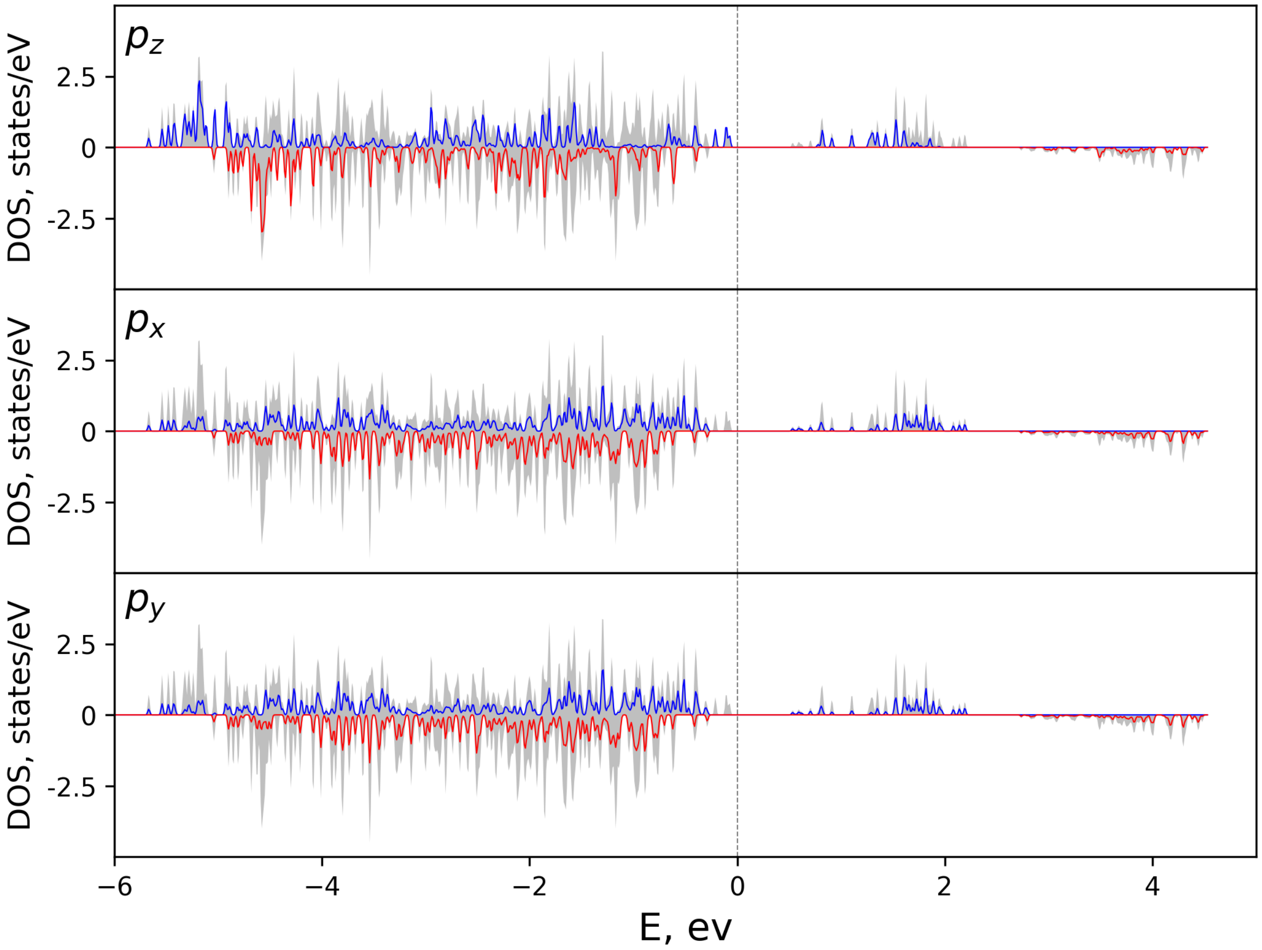

Figure S4. Orbitally-resolved density of states for S atoms of CrSBr monolayer for U = 3 eV. Spin up (blue), spin down (red) and total DOS (grey background) of S atoms.

2. Exchange Hamiltonian

We adopt an exchange Hamiltonian in the following form:

$$\hat{H} = \sum_{n=1}^{3}\left(-J_n \sum_{ij} \hat{\vec{S}}_i \hat{\vec{S}}_j - J_n^z \sum_{ij} \hat{S}_i^z \hat{S}_j^z\right) - D \sum_i (\hat{S}_i^z)^2 \quad (1)$$

where the sum over n runs over the nearest neighbors up to the third order (note that for the sites i and j the sum contains two terms: ij and ji i.e., there is a double counting), $\hat{\vec{S}}_i$, $\hat{\vec{S}}_j$ - spins of magnetic Cr atoms (normalized to 1). J_n - isotropic exchange parameters, J_n^z- anisotropic exchange parameters, D - single ion anisotropy. Comparison of calculated isotropic exchange parameters with the ones reported in the literature is provided in Table S1. Evolution of exchange parameters with Hubbard U for unstrained CrSBr monolayer is presented in Table S2. We used the value of single ion parameter D = 8.16 μeV reported in the literature [(4)].

Table S1. Isotropic exchange parameters from our calculations (U = 3 eV) and from literature in notation of eq. 1.

Reference	J_1 (meV)	J_2 (meV)	J_3 (meV)
Our calculations	3.54	3.08	4.14
Guo, et al. [1] (calc.)	3.87	7.32	-
Xu, et al. [2] (calc.)	15.82	16.13	-
Yang, et al. [3] (calc.)	3.62	4.62	3.13
Wang, et al. [4] (calc.)	5.68	6.23	2.11
Scheie, et al. [5] (exp.)	4.27	7.61	3.76

Table S2. Isotropic exchange parameters as a function of Hubbard U for single-layer CrSBr.

U, eV	1	2	3	4	5	6
J_1, meV	2.67	3.11	3.54	3.93	4.30	4.51
J_2, meV	3.48	3.29	3.08	2.87	2.63	2.43
J_3, meV	6.38	5.36	4.15	2.82	1.21	-0.64

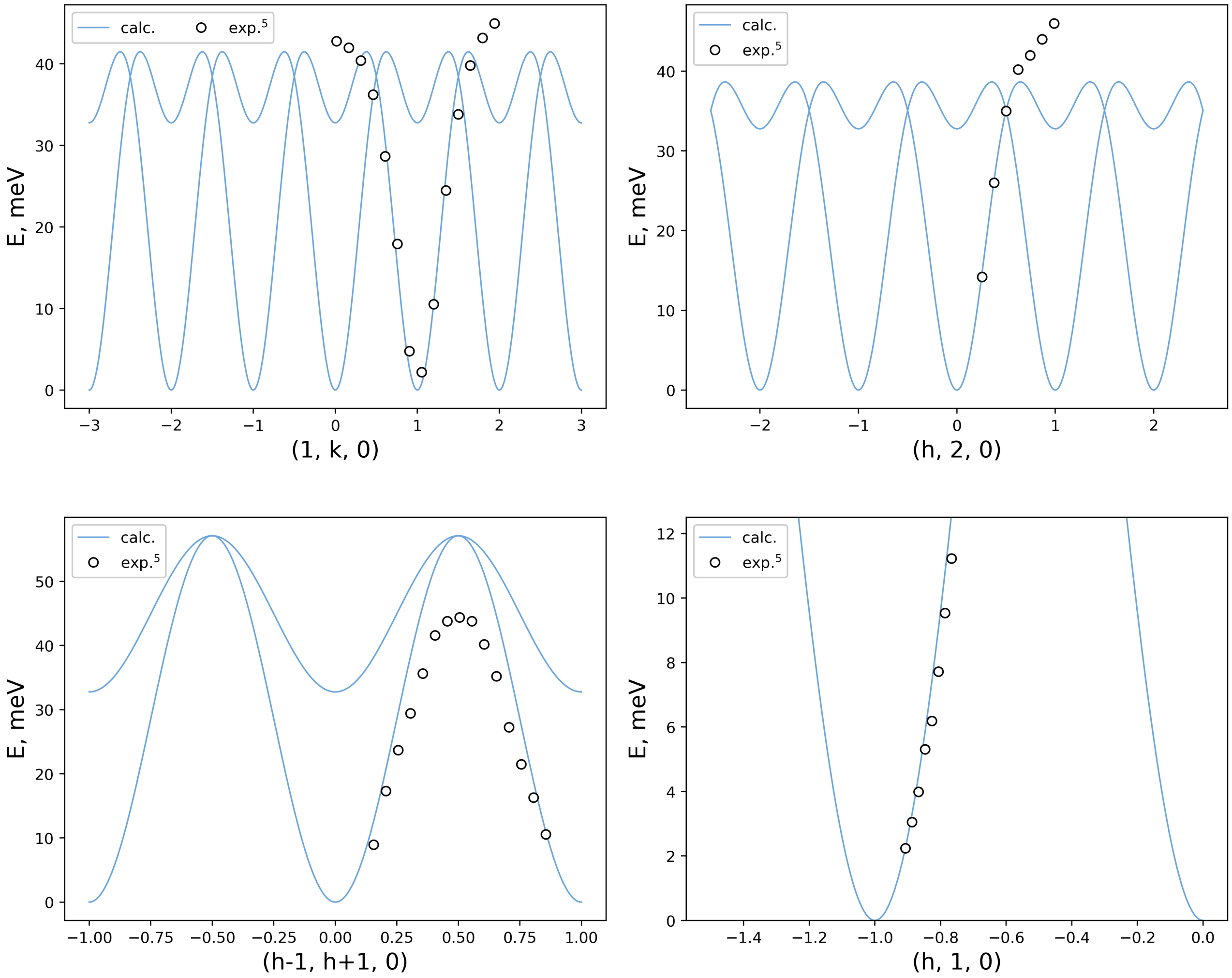

Figure S5. Calculated magnon dispersion (blue) with J values for U = 3 eV vs experimental data extracted from the fitted magnon dispersion by Scheie et al.[5]

3. Orbital-resolved analysis

In this section we present orbital-resolved exchange parameters with respect to Hubbard U (subsection 1), uniaxial strain along *a* (subsection 3) and *b* (subsection 4), as well as the occupations vs Hubbard U (subsection 2, Fig. S17). Note that for J_1 and J_2 neighbors are separated in two groups since some exchange pathways are mirrored between those two groups. Coordinate system adopted for the orbital-resolved calculations is defined as follows: z axis is directed along *b* crystallographic axis, x axis is directed along Cr-Br bond, y axis is defined in a way that xyz coordinate system is right-handed.

3.1 Isotropic exchange vs Hubbard U (ε = 0%)

Table S3. Orbital resolved isotropic exchange parameters under Coulomb screening.

	J_1, meV			J_2, meV			J_3, meV		
**U, eV**	t_{2g}-t_{2g}	t_{2g}-e_g	e_g-e_g	t_{2g}-t_{2g}	t_{2g}-e_g	e_g-e_g	t_{2g}-t_{2g}	t_{2g}-e_g	e_g-e_g
**1**	-3.88	6.93	-0.40	-1.20	5.85	-1.16	-0.74	9.56	-2.44
**2**	-3.11	6.69	-0.51	-0.91	5.75	-1.53	-0.64	9.44	-3.43
**3**	-2.60	6.76	-0.66	-0.74	5.83	-1.99	-0.56	9.46	-4.73
**4**	-2.24	7.00	-0.85	-0.62	6.07	-2.54	-0.50	9.68	-6.36
**5**	-1.99	7.34	-1.11	-0.55	6.39	-3.18	-0.45	10.04	-8.30
**6**	-1.85	7.71	-1.42	-0.50	6.83	-3.84	-0.41	10.35	-10.47

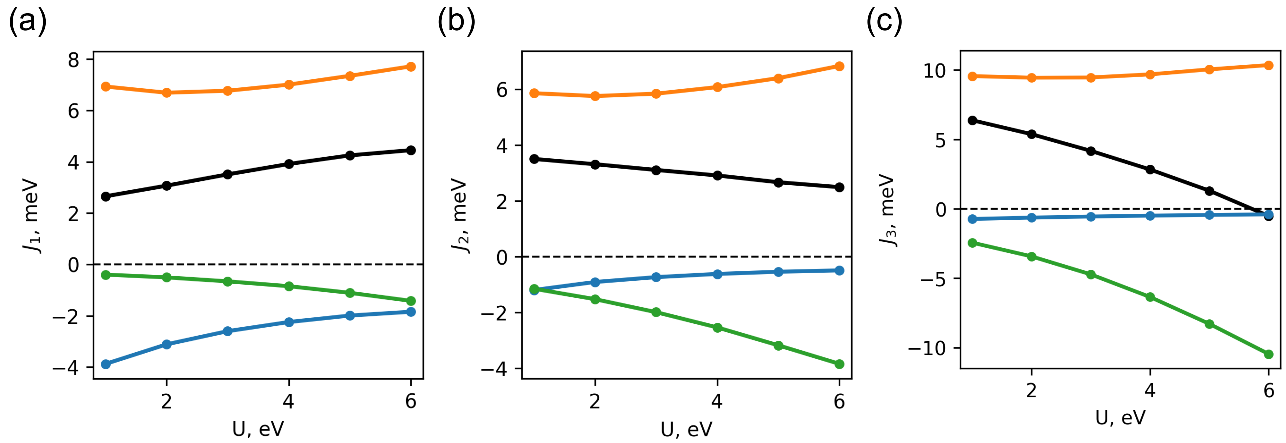

Figure S6. Orbital-resolved isotropic exchange parameters: (a) J_1; (b) J_2; (c) J_3, for the environmental Coulomb screening. Black color corresponds to the total parameter J_i, green - to the e_g-e_g exchange channel, orange - to the t_{2g}-e_g exchange channel and blue - to the t_{2g}-t_{2g} exchange channel.

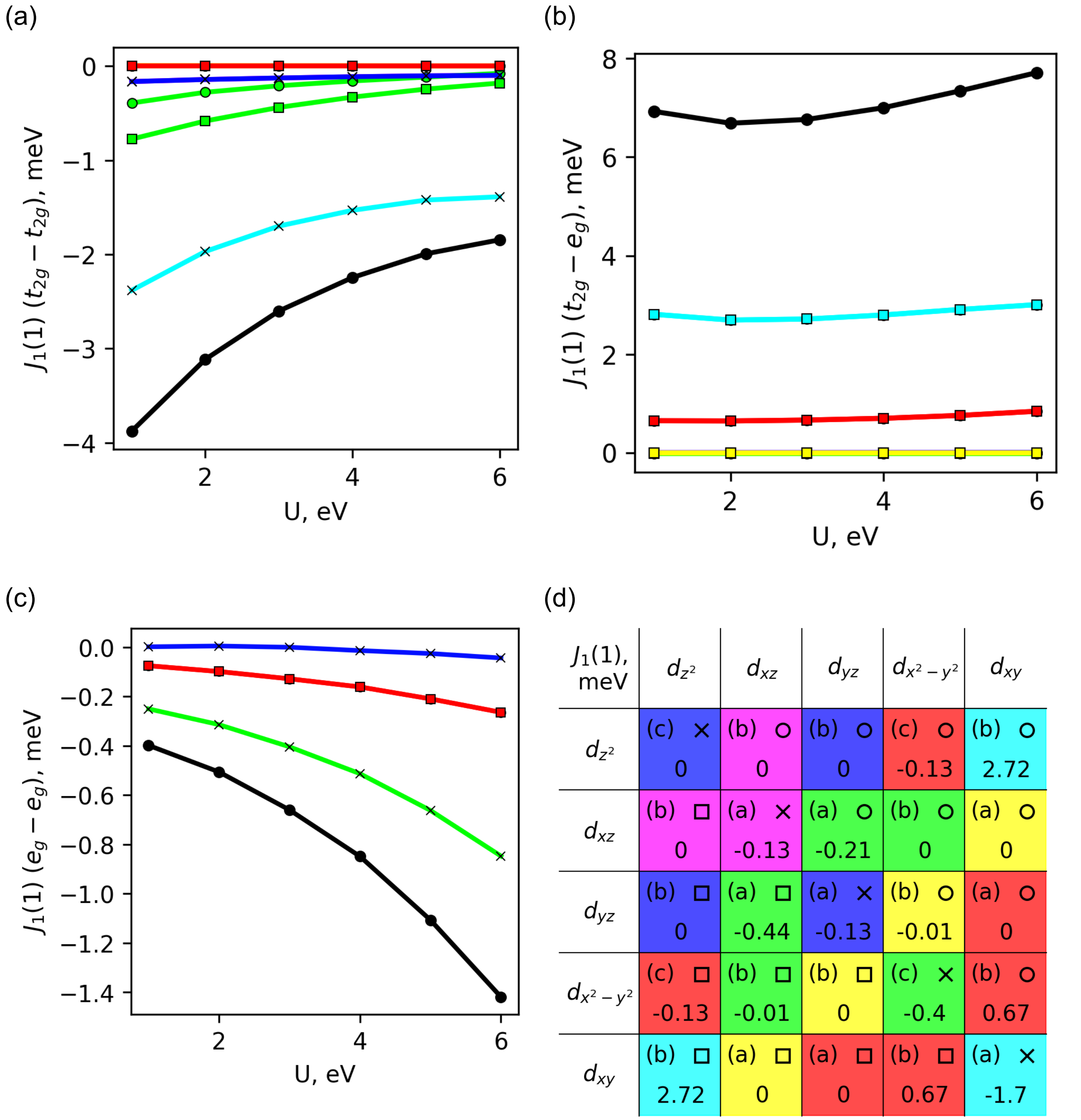

$J_1(1)$, meV	d_{z^2}	d_{xz}	d_{yz}	$d_{x^2-y^2}$	d_{xy}
d_{z^2}	(c) × 0	(b) ○ 0	(b) ○ 0	(c) ○ -0.13	(b) ○ 2.72
d_{xz}	(b) □ 0	(a) × -0.13	(a) ○ -0.21	(b) ○ 0	(a) ○ 0
d_{yz}	(b) □ 0	(a) □ -0.44	(a) × -0.13	(b) ○ -0.01	(a) ○ 0
$d_{x^2-y^2}$	(c) □ -0.13	(b) □ -0.01	(b) □ 0	(c) × -0.4	(b) ○ 0.67
d_{xy}	(b) □ 2.72	(a) □ 0	(a) □ 0	(b) □ 0.67	(a) × -1.7

Figure S7. Orbital-resolved isotropic exchange parameter $J_1(1)$ with variation of Hubbard U: (a) t_{2g}-t_{2g}; (b) t_{2g}-e_g; (c) e_g-e_g; (d) Color code for the graphs and values for U = 3 eV. Black color corresponds to the total exchange of each mechanism (t_{2g}-t_{2g}, t_{2g}-e_g or e_g-e_g).

U = 1 eV

$J_1(1)$, meV	d_{z^2}	d_{xz}	d_{yz}	$d_{x^2-y^2}$	d_{xy}
d_{z^2}	0	0	0	-0.08	2.81
d_{xz}	0	-0.16	-0.39	0	0
d_{yz}	0	-0.78	-0.16	-0.01	0
$d_{x^2-y^2}$	-0.08	-0.01	0	-0.25	0.65
d_{xy}	2.81	0	0	0.65	-2.38

U = 2 eV

$J_1(1)$, meV	d_{z^2}	d_{xz}	d_{yz}	$d_{x^2-y^2}$	d_{xy}
d_{z^2}	0	0	0	-0.1	2.7
d_{xz}	0	-0.14	-0.28	0	0
d_{yz}	0	-0.58	-0.14	-0.01	0
$d_{x^2-y^2}$	-0.1	-0.01	0	-0.31	0.65
d_{xy}	2.7	0	0	0.65	-1.97

U = 3 eV

$J_1(1)$, meV	d_{z^2}	d_{xz}	d_{yz}	$d_{x^2-y^2}$	d_{xy}
d_{z^2}	0	0	0	-0.13	2.72
d_{xz}	0	-0.13	-0.21	0	0
d_{yz}	0	-0.44	-0.13	-0.01	0
$d_{x^2-y^2}$	-0.13	-0.01	0	-0.4	0.67
d_{xy}	2.72	0	0	0.67	-1.7

U = 4 eV

$J_1(1)$, meV	d_{z^2}	d_{xz}	d_{yz}	$d_{x^2-y^2}$	d_{xy}
d_{z^2}	-0.01	0	0	-0.16	2.8
d_{xz}	0	-0.11	-0.16	0	0
d_{yz}	0	-0.33	-0.11	-0.01	0
$d_{x^2-y^2}$	-0.16	-0.01	0	-0.51	0.7
d_{xy}	2.8	0	0	0.7	-1.53

U = 5 eV

$J_1(1)$, meV	d_{z^2}	d_{xz}	d_{yz}	$d_{x^2-y^2}$	d_{xy}
d_{z^2}	-0.03	0	0	-0.21	2.91
d_{xz}	0	-0.1	-0.12	0	0
d_{yz}	0	-0.24	-0.1	-0.01	0
$d_{x^2-y^2}$	-0.21	-0.01	0	-0.66	0.76
d_{xy}	2.91	0	0	0.76	-1.42

U = 6 eV

$J_1(1)$, meV	d_{z^2}	d_{xz}	d_{yz}	$d_{x^2-y^2}$	d_{xy}
d_{z^2}	-0.04	0.01	0	-0.26	3.01
d_{xz}	0	-0.1	-0.08	0	0
d_{yz}	0.01	-0.18	-0.1	-0.01	0
$d_{x^2-y^2}$	-0.26	-0.01	0	-0.85	0.85
d_{xy}	3.01	0	0	0.85	-1.39

Figure S8. Orbital-resolved isotropic exchange parameter $J_1(1)$ with variation of Hubbard U. Color code: t_{2g}-t_{2g} - blue; t_{2g}-e_g - orange; e_g-e_g – green.

(a) (b)

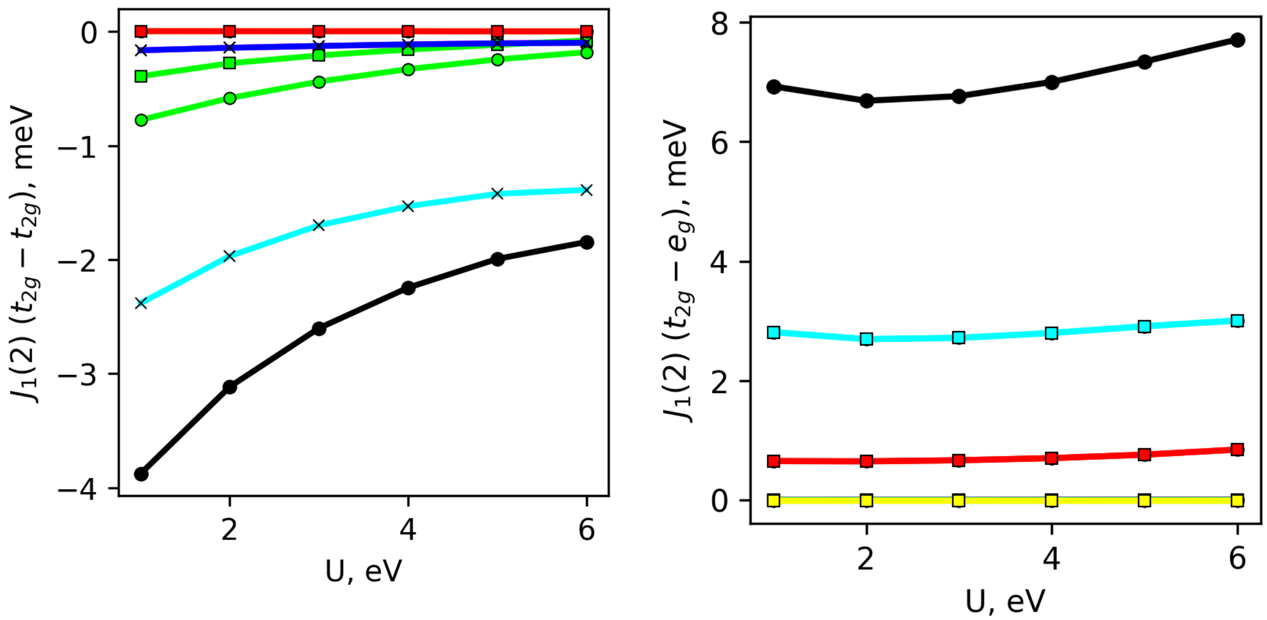

(c) (d)

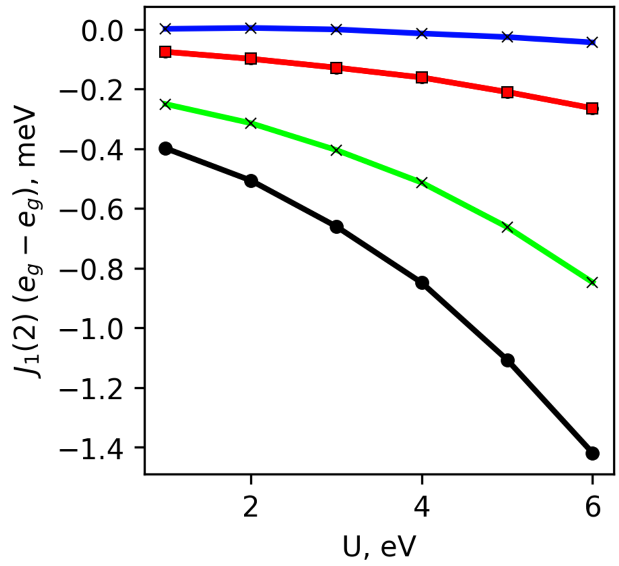

$J_1(2)$, meV	d_{z^2}	d_{xz}	d_{yz}	$d_{x^2-y^2}$	d_{xy}
d_{z^2}	(c) × 0	(b) ○ 0	(b) ○ 0	(c) ○ -0.13	(b) ○ 2.72
d_{xz}	(b) □ 0	(a) × -0.13	(a) ○ -0.44	(b) ○ -0.01	(a) ○ 0
d_{yz}	(b) □ 0	(a) □ -0.21	(a) × -0.13	(b) ○ 0	(a) ○ 0
$d_{x^2-y^2}$	(c) □ -0.13	(b) □ 0	(b) □ -0.01	(c) × -0.4	(b) ○ 0.67
d_{xy}	(b) □ 2.72	(a) □ 0	(a) □ 0	(b) □ 0.67	(a) × -1.7

Figure S9. Orbital-resolved isotropic exchange parameter $J_1(2)$ with variation of Hubbard U: (a) t_{2g}-t_{2g}; (b) t_{2g}-e_g; (c) e_g-e_g; (d) Color code for the graphs and values for U = 3 eV. Black color corresponds to the total exchange of each mechanism (t_{2g}-t_{2g}, t_{2g}-e_g or e_g-e_g).

U = 1 eV

$J_1(2)$, meV	d_{z^2}	d_{xz}	d_{yz}	$d_{x^2-y^2}$	d_{xy}
d_{z^2}	0	0	0	-0.08	2.81
d_{xz}	0	-0.16	-0.78	-0.01	0
d_{yz}	0	-0.39	-0.16	0	0
$d_{x^2-y^2}$	-0.08	0	-0.01	-0.25	0.65
d_{xy}	2.81	0	0	0.65	-2.38

U = 2 eV

$J_1(2)$, meV	d_{z^2}	d_{xz}	d_{yz}	$d_{x^2-y^2}$	d_{xy}
d_{z^2}	0	0	0	-0.1	2.7
d_{xz}	0	-0.14	-0.58	-0.01	0
d_{yz}	0	-0.28	-0.14	0	0
$d_{x^2-y^2}$	-0.1	0	-0.01	-0.31	0.65
d_{xy}	2.7	0	0	0.65	-1.97

U = 3 eV

$J_1(2)$, meV	d_{z^2}	d_{xz}	d_{yz}	$d_{x^2-y^2}$	d_{xy}
d_{z^2}	0	0	0	-0.13	2.72
d_{xz}	0	-0.13	-0.44	-0.01	0
d_{yz}	0	-0.21	-0.13	0	0
$d_{x^2-y^2}$	-0.13	0	-0.01	-0.4	0.67
d_{xy}	2.72	0	0	0.67	-1.7

U = 4 eV

$J_1(2)$, meV	d_{z^2}	d_{xz}	d_{yz}	$d_{x^2-y^2}$	d_{xy}
d_{z^2}	-0.01	0	0	-0.16	2.8
d_{xz}	0	-0.11	-0.33	-0.01	0
d_{yz}	0	-0.16	-0.11	0	0
$d_{x^2-y^2}$	-0.16	0	-0.01	-0.51	0.7
d_{xy}	2.8	0	0	0.7	-1.53

U = 5 eV

$J_1(2)$, meV	d_{z^2}	d_{xz}	d_{yz}	$d_{x^2-y^2}$	d_{xy}
d_{z^2}	-0.03	0	0	-0.21	2.91
d_{xz}	0	-0.1	-0.24	-0.01	0
d_{yz}	0	-0.12	-0.1	0	0
$d_{x^2-y^2}$	-0.21	0	-0.01	-0.66	0.76
d_{xy}	2.91	0	0	0.76	-1.42

U = 6 eV

$J_1(2)$, meV	d_{z^2}	d_{xz}	d_{yz}	$d_{x^2-y^2}$	d_{xy}
d_{z^2}	-0.04	0	0.01	-0.26	3.01
d_{xz}	0.01	-0.1	-0.18	-0.01	0
d_{yz}	0	-0.08	-0.1	0	0
$d_{x^2-y^2}$	-0.26	0	-0.01	-0.85	0.85
d_{xy}	3.01	0	0	0.85	-1.39

Figure S10. Orbital-resolved isotropic exchange parameter $J_1(2)$ with variation of Hubbard U. Color code: t_{2g}-t_{2g} - blue; t_{2g}-e_g - orange; e_g-e_g – green.

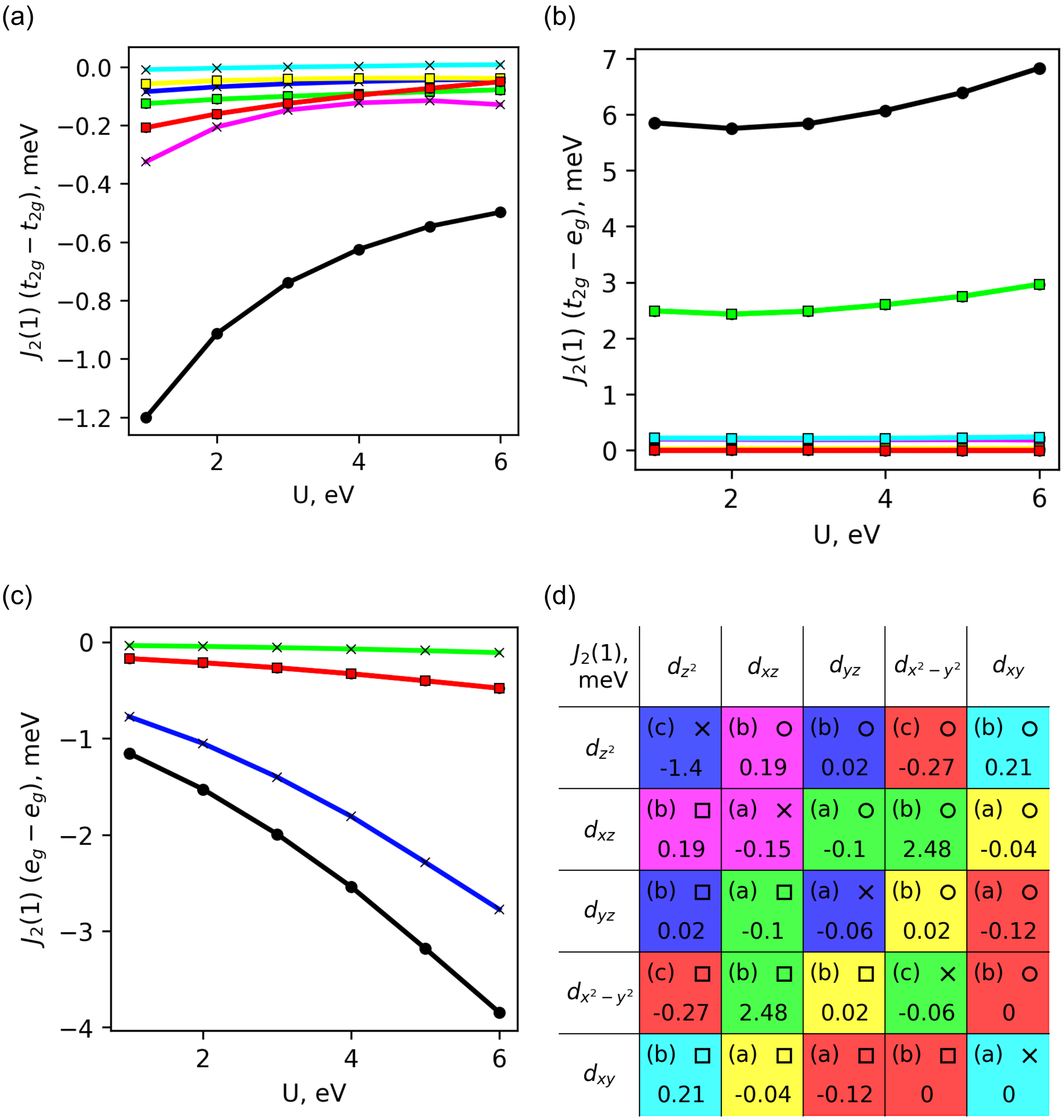

$J_2(1)$, meV	d_{z^2}	d_{xz}	d_{yz}	$d_{x^2-y^2}$	d_{xy}
d_{z^2}	(c) × -1.4	(b) ○ 0.19	(b) ○ 0.02	(c) ○ -0.27	(b) ○ 0.21
d_{xz}	(b) □ 0.19	(a) × -0.15	(a) ○ -0.1	(b) ○ 2.48	(a) ○ -0.04
d_{yz}	(b) □ 0.02	(a) □ -0.1	(a) × -0.06	(b) ○ 0.02	(a) ○ -0.12
$d_{x^2-y^2}$	(c) □ -0.27	(b) □ 2.48	(b) □ 0.02	(c) × -0.06	(b) ○ 0
d_{xy}	(b) □ 0.21	(a) □ -0.04	(a) □ -0.12	(b) □ 0	(a) × 0

Figure S11. Orbital-resolved isotropic exchange parameter $J_2(1)$ with variation of Hubbard U: (a) t_{2g}-t_{2g}; (b) t_{2g}-e_g; (c) e_g-e_g; (d) Color code for the graphs and values for U = 3 eV. Black color corresponds to the total exchange of each mechanism (t_{2g}-t_{2g}, t_{2g}-e_g or e_g-e_g).

U = 1 eV

$J_2(1)$, meV	d_{z^2}	d_{xz}	d_{yz}	$d_{x^2-y^2}$	d_{xy}
d_{z^2}	-0.77	0.2	0.01	-0.17	0.21
d_{xz}	0.2	-0.32	-0.13	2.49	-0.06
d_{yz}	0.01	-0.13	-0.08	0.02	-0.21
$d_{x^2-y^2}$	-0.17	2.49	0.02	-0.04	0
d_{xy}	0.21	-0.06	-0.21	0	-0.01

U = 2 eV

$J_2(1)$, meV	d_{z^2}	d_{xz}	d_{yz}	$d_{x^2-y^2}$	d_{xy}
d_{z^2}	-1.05	0.2	0.02	-0.22	0.21
d_{xz}	0.2	-0.21	-0.11	2.43	-0.05
d_{yz}	0.02	-0.11	-0.07	0.02	-0.16
$d_{x^2-y^2}$	-0.22	2.43	0.02	-0.05	0
d_{xy}	0.21	-0.05	-0.16	0	0

U = 3 eV

$J_2(1)$, meV	d_{z^2}	d_{xz}	d_{yz}	$d_{x^2-y^2}$	d_{xy}
d_{z^2}	-1.4	0.19	0.02	-0.27	0.21
d_{xz}	0.19	-0.15	-0.1	2.48	-0.04
d_{yz}	0.02	-0.1	-0.06	0.02	-0.12
$d_{x^2-y^2}$	-0.27	2.48	0.02	-0.06	0
d_{xy}	0.21	-0.04	-0.12	0	0

U = 4 eV

$J_2(1)$, meV	d_{z^2}	d_{xz}	d_{yz}	$d_{x^2-y^2}$	d_{xy}
d_{z^2}	-1.81	0.19	0.01	-0.33	0.21
d_{xz}	0.19	-0.12	-0.09	2.6	-0.04
d_{yz}	0.01	-0.09	-0.05	0.02	-0.1
$d_{x^2-y^2}$	-0.33	2.6	0.02	-0.07	0
d_{xy}	0.21	-0.04	-0.1	0	0

U = 5 eV

$J_2(1)$, meV	d_{z^2}	d_{xz}	d_{yz}	$d_{x^2-y^2}$	d_{xy}
d_{z^2}	-2.29	0.19	0.01	-0.4	0.22
d_{xz}	0.19	-0.12	-0.08	2.75	-0.04
d_{yz}	0.01	-0.08	-0.04	0.03	-0.07
$d_{x^2-y^2}$	-0.4	2.75	0.03	-0.09	-0.01
d_{xy}	0.22	-0.04	-0.07	-0.01	0.01

U = 6 eV

$J_2(1)$, meV	d_{z^2}	d_{xz}	d_{yz}	$d_{x^2-y^2}$	d_{xy}
d_{z^2}	-2.77	0.18	0.01	-0.48	0.23
d_{xz}	0.18	-0.13	-0.08	2.96	-0.04
d_{yz}	0.01	-0.08	-0.04	0.03	-0.05
$d_{x^2-y^2}$	-0.48	2.96	0.03	-0.11	-0.01
d_{xy}	0.23	-0.04	-0.05	-0.01	0.01

Figure S12. Orbital-resolved isotropic exchange parameter $J_2(1)$ with variation of Hubbard U. Color code: t_{2g}-t_{2g} - blue; t_{2g}-e_g - orange; e_g-e_g - green

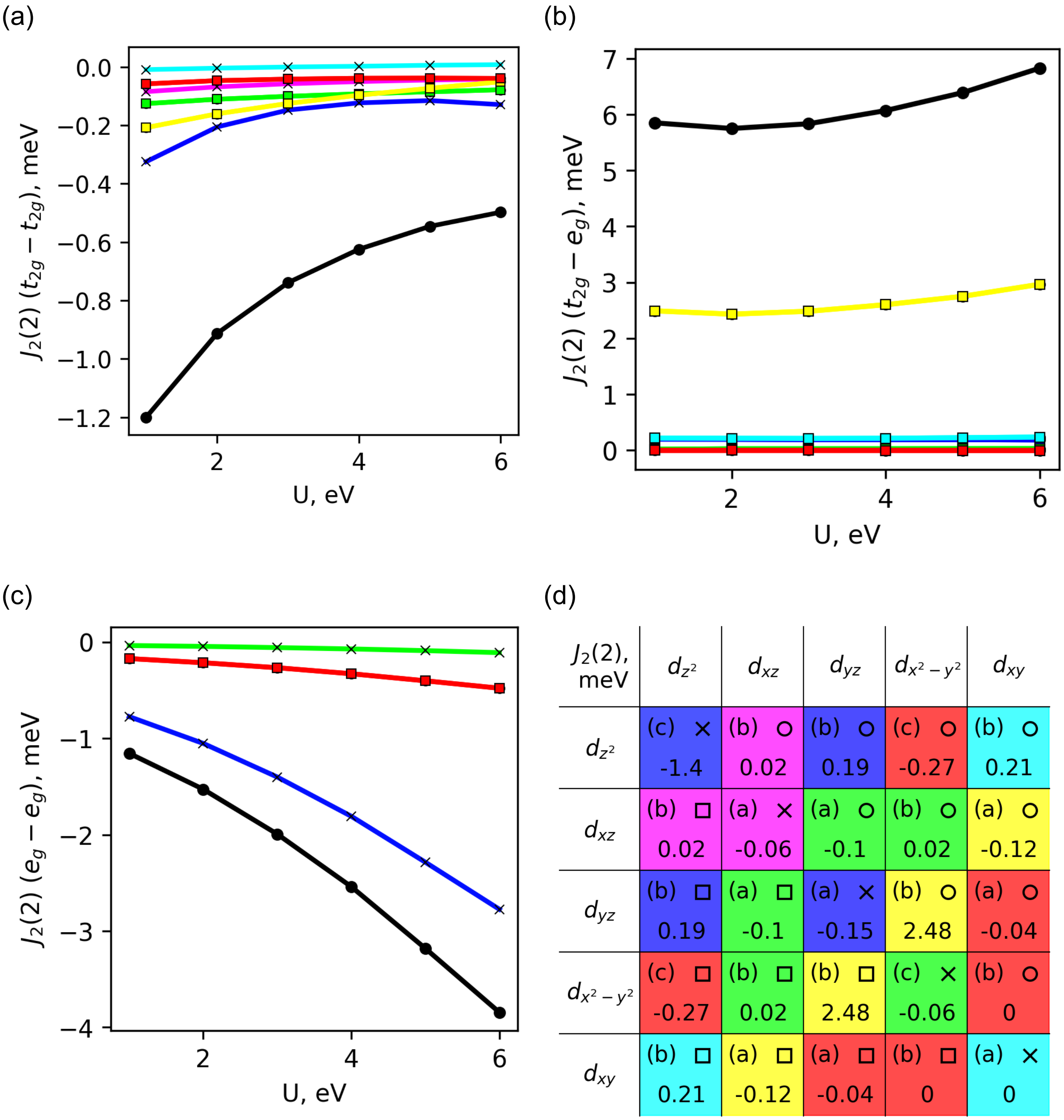

$J_2(2)$, meV	d_{z^2}	d_{xz}	d_{yz}	$d_{x^2-y^2}$	d_{xy}
d_{z^2}	(c) × -1.4	(b) ○ 0.02	(b) ○ 0.19	(c) ○ -0.27	(b) ○ 0.21
d_{xz}	(b) □ 0.02	(a) × -0.06	(a) ○ -0.1	(b) ○ 0.02	(a) ○ -0.12
d_{yz}	(b) □ 0.19	(a) □ -0.1	(a) × -0.15	(b) ○ 2.48	(a) ○ -0.04
$d_{x^2-y^2}$	(c) □ -0.27	(b) □ 0.02	(b) □ 2.48	(c) × -0.06	(b) ○ 0
d_{xy}	(b) □ 0.21	(a) □ -0.12	(a) □ -0.04	(b) □ 0	(a) × 0

Figure S13. Orbital-resolved isotropic exchange parameter $J_2(2)$ with variation of Hubbard U: (a) t_{2g}-t_{2g}; (b) t_{2g}-e_g; (c) e_g-e_g; (d) Color code for the graphs and values for U = 3 eV. Black color corresponds to the total exchange of each mechanism (t_{2g}-t_{2g}, t_{2g}-e_g or e_g-e_g).

U = 1 eV

$J_2(2)$, meV	d_{z^2}	d_{xz}	d_{yz}	$d_{x^2-y^2}$	d_{xy}
d_{z^2}	-0.77	0.01	0.2	-0.17	0.21
d_{xz}	0.01	-0.08	-0.13	0.02	-0.21
d_{yz}	0.2	-0.13	-0.32	2.49	-0.06
$d_{x^2-y^2}$	-0.17	0.02	2.49	-0.04	0
d_{xy}	0.21	-0.21	-0.06	0	-0.01

U = 2 eV

$J_2(2)$, meV	d_{z^2}	d_{xz}	d_{yz}	$d_{x^2-y^2}$	d_{xy}
d_{z^2}	-1.05	0.02	0.2	-0.22	0.21
d_{xz}	0.02	-0.07	-0.11	0.02	-0.16
d_{yz}	0.2	-0.11	-0.21	2.43	-0.05
$d_{x^2-y^2}$	-0.22	0.02	2.43	-0.05	0
d_{xy}	0.21	-0.16	-0.05	0	0

U = 3 eV

$J_2(2)$, meV	d_{z^2}	d_{xz}	d_{yz}	$d_{x^2-y^2}$	d_{xy}
d_{z^2}	-1.4	0.02	0.19	-0.27	0.21
d_{xz}	0.02	-0.06	-0.1	0.02	-0.12
d_{yz}	0.19	-0.1	-0.15	2.48	-0.04
$d_{x^2-y^2}$	-0.27	0.02	2.48	-0.06	0
d_{xy}	0.21	-0.12	-0.04	0	0

U = 4 eV

$J_2(2)$, meV	d_{z^2}	d_{xz}	d_{yz}	$d_{x^2-y^2}$	d_{xy}
d_{z^2}	-1.81	0.01	0.19	-0.33	0.21
d_{xz}	0.01	-0.05	-0.09	0.02	-0.1
d_{yz}	0.19	-0.09	-0.12	2.6	-0.04
$d_{x^2-y^2}$	-0.33	0.02	2.6	-0.07	0
d_{xy}	0.21	-0.1	-0.04	0	0

U = 5 eV

$J_2(2)$, meV	d_{z^2}	d_{xz}	d_{yz}	$d_{x^2-y^2}$	d_{xy}
d_{z^2}	-2.29	0.01	0.19	-0.4	0.22
d_{xz}	0.01	-0.04	-0.08	0.03	-0.07
d_{yz}	0.19	-0.08	-0.12	2.75	-0.04
$d_{x^2-y^2}$	-0.4	0.03	2.75	-0.09	-0.01
d_{xy}	0.22	-0.07	-0.04	-0.01	0.01

U = 6 eV

$J_2(2)$, meV	d_{z^2}	d_{xz}	d_{yz}	$d_{x^2-y^2}$	d_{xy}
d_{z^2}	-2.77	0.01	0.18	-0.48	0.23
d_{xz}	0.01	-0.04	-0.08	0.03	-0.05
d_{yz}	0.18	-0.08	-0.13	2.96	-0.04
$d_{x^2-y^2}$	-0.48	0.03	2.96	-0.11	-0.01
d_{xy}	0.23	-0.05	-0.04	-0.01	0.01

Figure S14. Orbital-resolved isotropic exchange parameter $J_2(2)$ with variation of Hubbard U. Color code: t_{2g}-t_{2g} - blue; t_{2g}-e_g - orange; e_g-e_g – green.

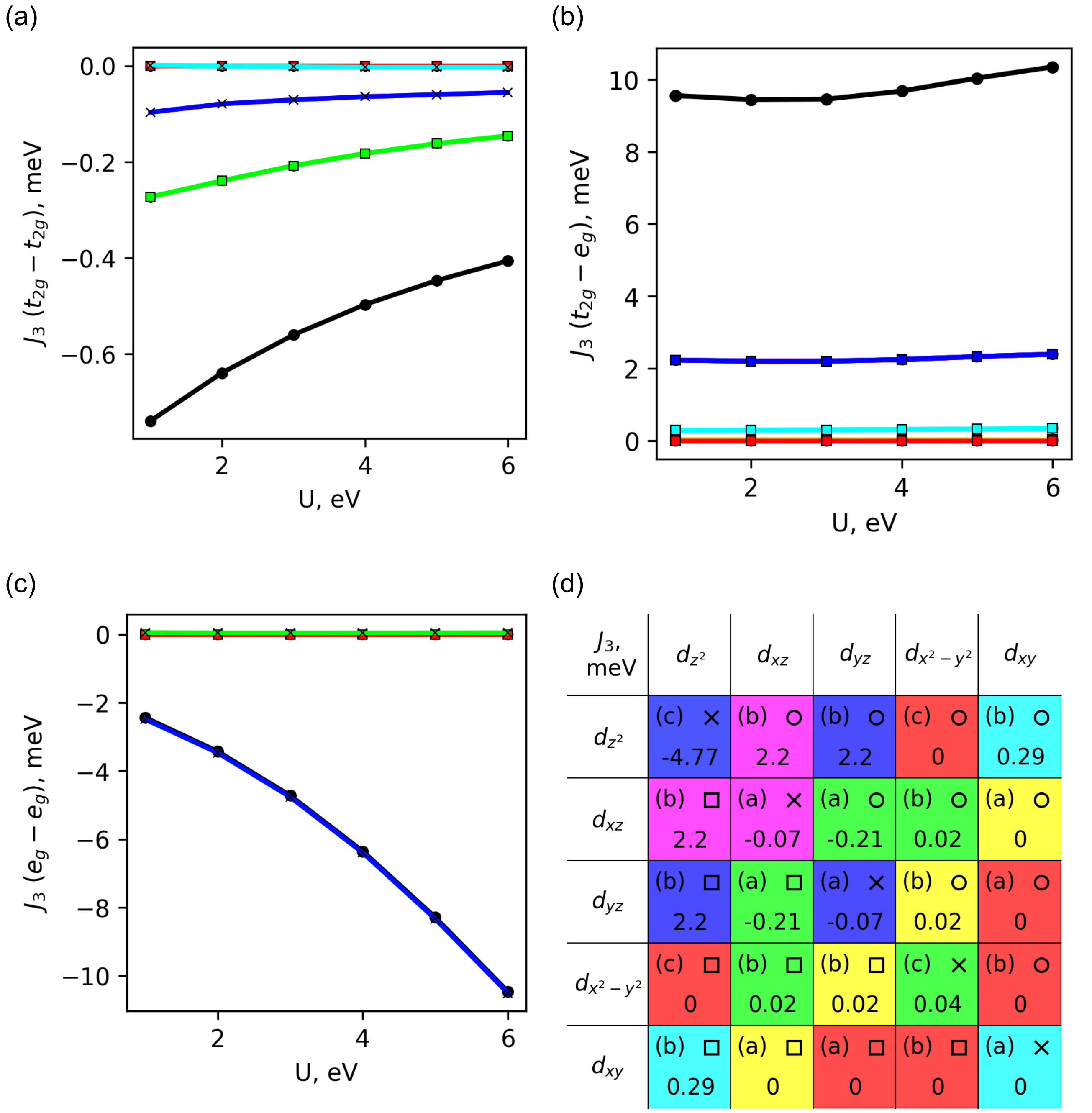

J_3, meV	d_{z^2}	d_{xz}	d_{yz}	$d_{x^2-y^2}$	d_{xy}
d_{z^2}	(c) × -4.77	(b) ○ 2.2	(b) ○ 2.2	(c) ○ 0	(b) ○ 0.29
d_{xz}	(b) □ 2.2	(a) × -0.07	(a) ○ -0.21	(b) ○ 0.02	(a) ○ 0
d_{yz}	(b) □ 2.2	(a) □ -0.21	(a) × -0.07	(b) ○ 0.02	(a) ○ 0
$d_{x^2-y^2}$	(c) □ 0	(b) □ 0.02	(b) □ 0.02	(c) × 0.04	(b) ○ 0
d_{xy}	(b) □ 0.29	(a) □ 0	(a) □ 0	(b) □ 0	(a) × 0

Figure S15. Orbital-resolved isotropic exchange parameter J_3 with variation of Hubbard U: (a) t_{2g}-t_{2g}; (b) t_{2g}-e_g; (c) e_g-e_g; (d) Color code for the graphs and values for U = 3 eV. Black color corresponds to the total exchange of each mechanism (t_{2g}-t_{2g}, t_{2g}-e_g or e_g-e_g).

U = 1 eV

J_3, meV	d_{z^2}	d_{xz}	d_{yz}	$d_{x^2-y^2}$	d_{xy}
d_{z^2}	-2.49	2.23	2.23	0	0.28
d_{xz}	2.23	-0.1	-0.27	0.02	0
d_{yz}	2.23	-0.27	-0.1	0.02	0
$d_{x^2-y^2}$	0	0.02	0.02	0.05	0
d_{xy}	0.28	0	0	0	0

U = 2 eV

J_3, meV	d_{z^2}	d_{xz}	d_{yz}	$d_{x^2-y^2}$	d_{xy}
d_{z^2}	-3.47	2.2	2.2	0	0.29
d_{xz}	2.2	-0.08	-0.24	0.02	0
d_{yz}	2.2	-0.24	-0.08	0.02	0
$d_{x^2-y^2}$	0	0.02	0.02	0.04	0
d_{xy}	0.29	0	0	0	0

U = 3 eV

J_3, meV	d_{z^2}	d_{xz}	d_{yz}	$d_{x^2-y^2}$	d_{xy}
d_{z^2}	-4.77	2.2	2.2	0	0.29
d_{xz}	2.2	-0.07	-0.21	0.02	0
d_{yz}	2.2	-0.21	-0.07	0.02	0
$d_{x^2-y^2}$	0	0.02	0.02	0.04	0
d_{xy}	0.29	0	0	0	0

U = 4 eV

J_3, meV	d_{z^2}	d_{xz}	d_{yz}	$d_{x^2-y^2}$	d_{xy}
d_{z^2}	-6.4	2.24	2.24	0	0.31
d_{xz}	2.24	-0.06	-0.18	0.02	0
d_{yz}	2.24	-0.18	-0.06	0.02	0
$d_{x^2-y^2}$	0	0.02	0.02	0.04	0
d_{xy}	0.31	0	0	0	0

U = 5 eV

J_3, meV	d_{z^2}	d_{xz}	d_{yz}	$d_{x^2-y^2}$	d_{xy}
d_{z^2}	-8.34	2.33	2.33	0	0.32
d_{xz}	2.33	-0.06	-0.16	0.02	0
d_{yz}	2.33	-0.16	-0.06	0.02	0
$d_{x^2-y^2}$	0	0.02	0.02	0.04	0
d_{xy}	0.32	0	0	0	0

U = 6 eV

J_3, meV	d_{z^2}	d_{xz}	d_{yz}	$d_{x^2-y^2}$	d_{xy}
d_{z^2}	-10.51	2.39	2.39	0	0.34
d_{xz}	2.39	-0.06	-0.15	0.03	0
d_{yz}	2.39	-0.15	-0.06	0.03	0
$d_{x^2-y^2}$	0	0.03	0.03	0.04	0
d_{xy}	0.34	0	0	0	0

Figure S16. Orbital-resolved isotropic exchange parameter J_3 with variation of Hubbard U. Color code: t_{2g}-t_{2g} - blue; t_{2g}-e_g - orange; e_g-e_g – green.

3.2 Occupations vs Hubbard U (ε = 0%)

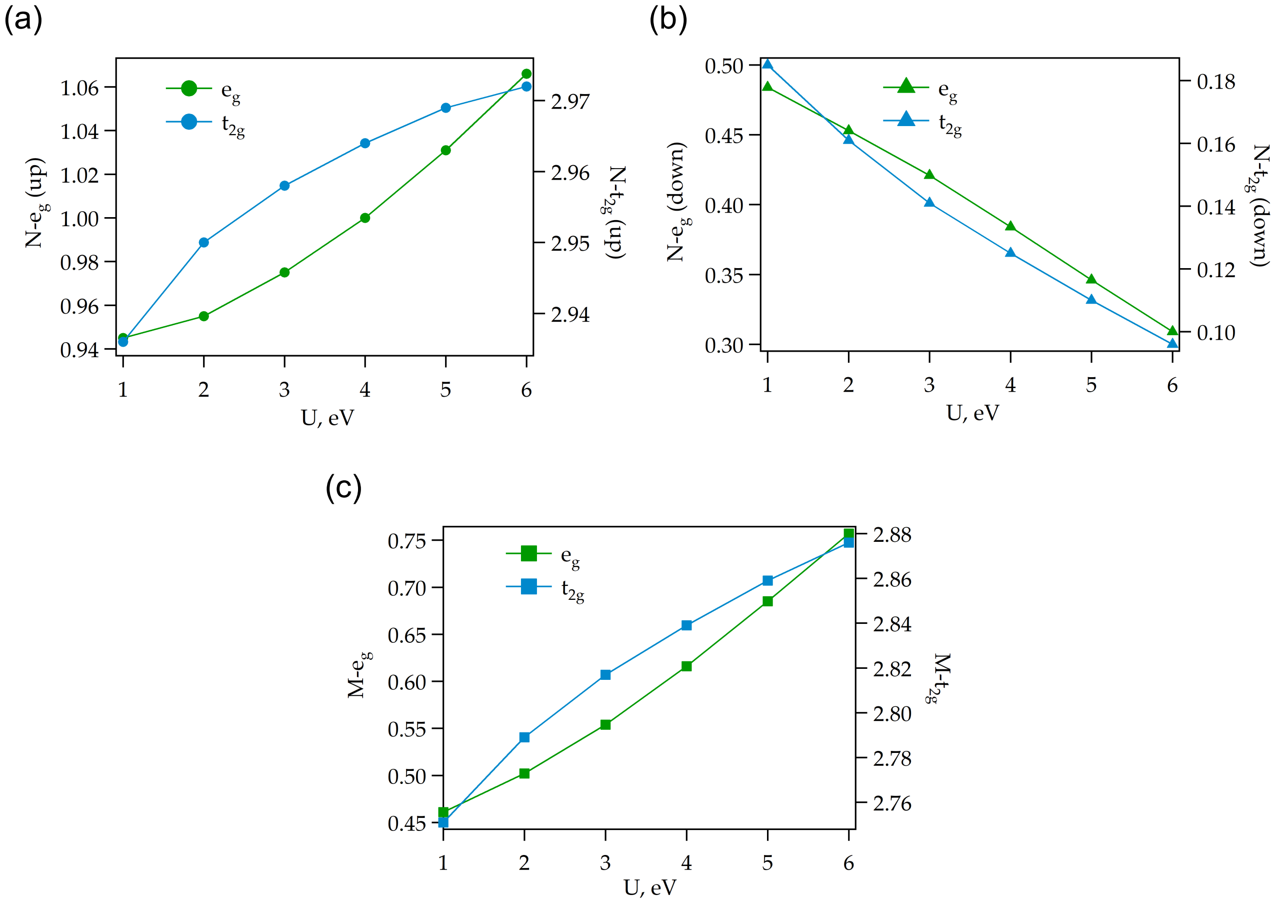

Figure S17. Occupation of *d* orbitals per Cr atom, both for t_{2g} and e_g orbitals in their (a) spin up and (b) spin down configurations. (c) total magnetic moments as a function of Hubbard U.

3.3 Isotropic exchange vs strain applied along *a* (U = 3 eV)

Table S4. Orbital resolved isotropic exchange parameters with strain applied along *a*.

	J_1, meV			J_2, meV			J_3, meV		
ε_a	t_{2g}-t_{2g}	t_{2g}-e_g	e_g-e_g	t_{2g}-t_{2g}	t_{2g}-e_g	e_g-e_g	t_{2g}-t_{2g}	t_{2g}-e_g	e_g-e_g
**-5%**	-5.18	6.52	-0.75	-0.89	5.94	-1.83	-0.55	8.75	-4.08
**-4%**	-4.52	6.60	-0.72	-0.86	5.89	-1.86	-0.55	8.90	-4.22
**-3%**	-3.94	6.67	-0.70	-0.83	5.85	-1.89	-0.55	9.00	-4.34
**-2%**	-3.43	6.75	-0.69	-0.80	5.84	-1.93	-0.55	9.18	-4.51
**-1%**	-2.99	6.77	-0.68	-0.77	5.84	-1.97	-0.55	9.33	-4.62
**0%**	-2.60	6.76	-0.66	-0.74	5.83	-1.99	-0.56	9.46	-4.77
**1%**	-2.26	6.75	-0.63	-0.71	5.86	-2.02	-0.57	9.57	-4.83
**2%**	-1.96	6.75	-0.60	-0.68	5.90	-2.05	-0.57	9.72	-4.95
**3%**	-1.70	6.69	-0.58	-0.66	5.94	-2.08	-0.59	9.85	-5.02
**4%**	-1.48	6.63	-0.53	-0.63	5.00	-2.10	-0.60	9.99	-5.10
**5%**	-1.29	6.54	-0.50	-0.61	6.06	-2.12	-0.61	10.10	-5.15

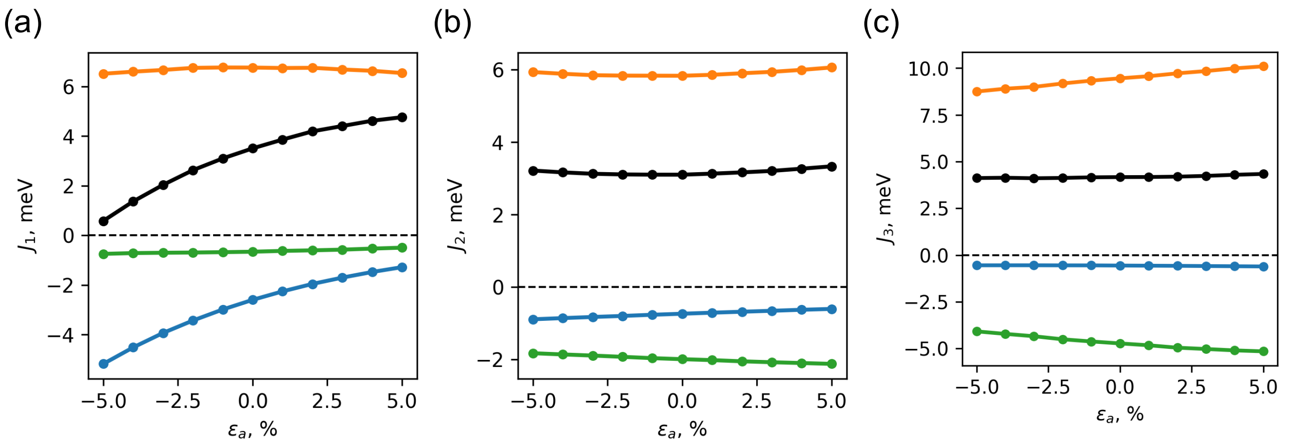

Figure S18. Orbital-resolved isotropic exchange parameters: (a) J_1; (b) J_2; (c) J_3, with strain applied along *a* axis. Black color corresponds to the total parameter J_i, green - to the e_g-e_g exchange channel, orange - to the t_{2g}-e_g exchange channel and blue - to the t_{2g}-t_{2g} exchange channel.

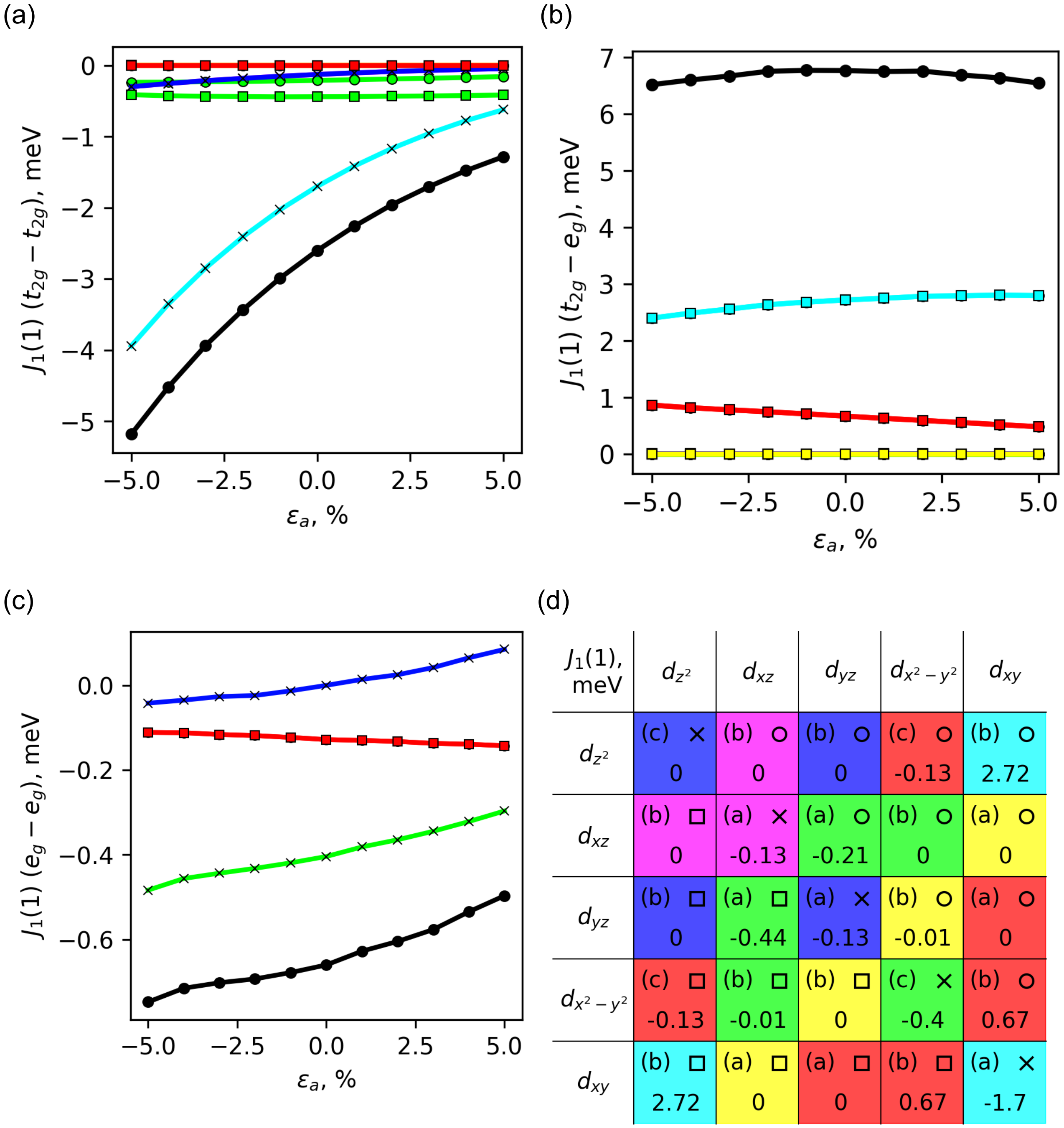

Figure S19. Orbital-resolved isotropic exchange parameter $J_1(1)$ with applied uniaxial strain along *a*. (a) t_{2g}-t_{2g}; (b) t_{2g}-e_g; (c) e_g-e_g; (d) Color code for the graphs and values for $\varepsilon_a = 0\%$. Black color corresponds to the total exchange of each mechanism (t_{2g}-t_{2g}, t_{2g}-e_g or e_g-e_g).

ε_a = -5%

$J_1(1)$, meV	d_{z^2}	d_{xz}	d_{yz}	$d_{x^2-y^2}$	d_{xy}
d_{z^2}	-0.04	0	0	-0.11	2.4
d_{xz}	0	-0.3	-0.24	0	0
d_{yz}	0	-0.41	-0.3	0	0
$d_{x^2-y^2}$	-0.11	0	0	-0.48	0.86
d_{xy}	2.4	0	0	0.86	-3.94

ε_a = -4%

$J_1(1)$, meV	d_{z^2}	d_{xz}	d_{yz}	$d_{x^2-y^2}$	d_{xy}
d_{z^2}	-0.04	0	0	-0.11	2.48
d_{xz}	0	-0.25	-0.23	0	0
d_{yz}	0	-0.43	-0.25	-0.01	0
$d_{x^2-y^2}$	-0.11	-0.01	0	-0.46	0.82
d_{xy}	2.48	0	0	0.82	-3.35

ε_a = -3%

$J_1(1)$, meV	d_{z^2}	d_{xz}	d_{yz}	$d_{x^2-y^2}$	d_{xy}
d_{z^2}	-0.03	0	0	-0.12	2.56
d_{xz}	0	-0.21	-0.23	0	0
d_{yz}	0	-0.43	-0.21	-0.01	0
$d_{x^2-y^2}$	-0.12	-0.01	0	-0.44	0.78
d_{xy}	2.56	0	0	0.78	-2.85

ε_a = -2%

$J_1(1)$, meV	d_{z^2}	d_{xz}	d_{yz}	$d_{x^2-y^2}$	d_{xy}
d_{z^2}	-0.02	0	0	-0.12	2.63
d_{xz}	0	-0.18	-0.23	0	0
d_{yz}	0	-0.44	-0.18	-0.01	0
$d_{x^2-y^2}$	-0.12	-0.01	0	-0.43	0.74
d_{xy}	2.63	0	0	0.74	-2.41

ε_a = -1%

$J_1(1)$, meV	d_{z^2}	d_{xz}	d_{yz}	$d_{x^2-y^2}$	d_{xy}
d_{z^2}	-0.01	0	0	-0.12	2.68
d_{xz}	0	-0.15	-0.22	0	0
d_{yz}	0	-0.44	-0.15	-0.01	0
$d_{x^2-y^2}$	-0.12	-0.01	0	-0.42	0.71
d_{xy}	2.68	0	0	0.71	-2.03

ε_a = 0%

$J_1(1)$, meV	d_{z^2}	d_{xz}	d_{yz}	$d_{x^2-y^2}$	d_{xy}
d_{z^2}	0	0	0	-0.13	2.72
d_{xz}	0	-0.13	-0.21	0	0
d_{yz}	0	-0.44	-0.13	-0.01	0
$d_{x^2-y^2}$	-0.13	-0.01	0	-0.4	0.67
d_{xy}	2.72	0	0	0.67	-1.7

ε_a = 1%

$J_1(1)$, meV	d_{z^2}	d_{xz}	d_{yz}	$d_{x^2-y^2}$	d_{xy}
d_{z^2}	0.01	0	0	-0.13	2.75
d_{xz}	0	-0.1	-0.2	0	0
d_{yz}	0	-0.44	-0.1	-0.01	0
$d_{x^2-y^2}$	-0.13	-0.01	0	-0.38	0.63
d_{xy}	2.75	0	0	0.63	-1.41

ε_a = 2%

$J_1(1)$, meV	d_{z^2}	d_{xz}	d_{yz}	$d_{x^2-y^2}$	d_{xy}
d_{z^2}	0.02	0.01	0	-0.13	2.78
d_{xz}	0	-0.08	-0.19	0	0
d_{yz}	0.01	-0.43	-0.08	0	0
$d_{x^2-y^2}$	-0.13	0	0	-0.36	0.59
d_{xy}	2.78	0	0	0.59	-1.17

ε_a = 3%

$J_1(1)$, meV	d_{z^2}	d_{xz}	d_{yz}	$d_{x^2-y^2}$	d_{xy}
d_{z^2}	0.04	0	0	-0.14	2.79
d_{xz}	0	-0.07	-0.18	0	0
d_{yz}	0	-0.43	-0.07	-0.01	0
$d_{x^2-y^2}$	-0.14	-0.01	0	-0.34	0.55
d_{xy}	2.79	0	0	0.55	-0.96

ε_a = 4%

$J_1(1)$, meV	d_{z^2}	d_{xz}	d_{yz}	$d_{x^2-y^2}$	d_{xy}
d_{z^2}	0.06	0	0	-0.14	2.8
d_{xz}	0	-0.06	-0.17	0	0
d_{yz}	0	-0.42	-0.06	-0.01	0
$d_{x^2-y^2}$	-0.14	-0.01	0	-0.32	0.52
d_{xy}	2.8	0	0	0.52	-0.78

ε_a = 5%

$J_1(1)$, meV	d_{z^2}	d_{xz}	d_{yz}	$d_{x^2-y^2}$	d_{xy}
d_{z^2}	0.08	0	0	-0.14	2.79
d_{xz}	0	-0.04	-0.16	0	0
d_{yz}	0	-0.42	-0.04	-0.01	0
$d_{x^2-y^2}$	-0.14	-0.01	0	-0.3	0.48
d_{xy}	2.79	0	0	0.48	-0.62

Color code:
t_{2g}-t_{2g}: blue
t_{2g}-e_g: orange
e_g-e_g: green

Figure S20. Orbital-resolved isotropic exchange parameter $J_1(1)$ with applied uniaxial strain along *a*.

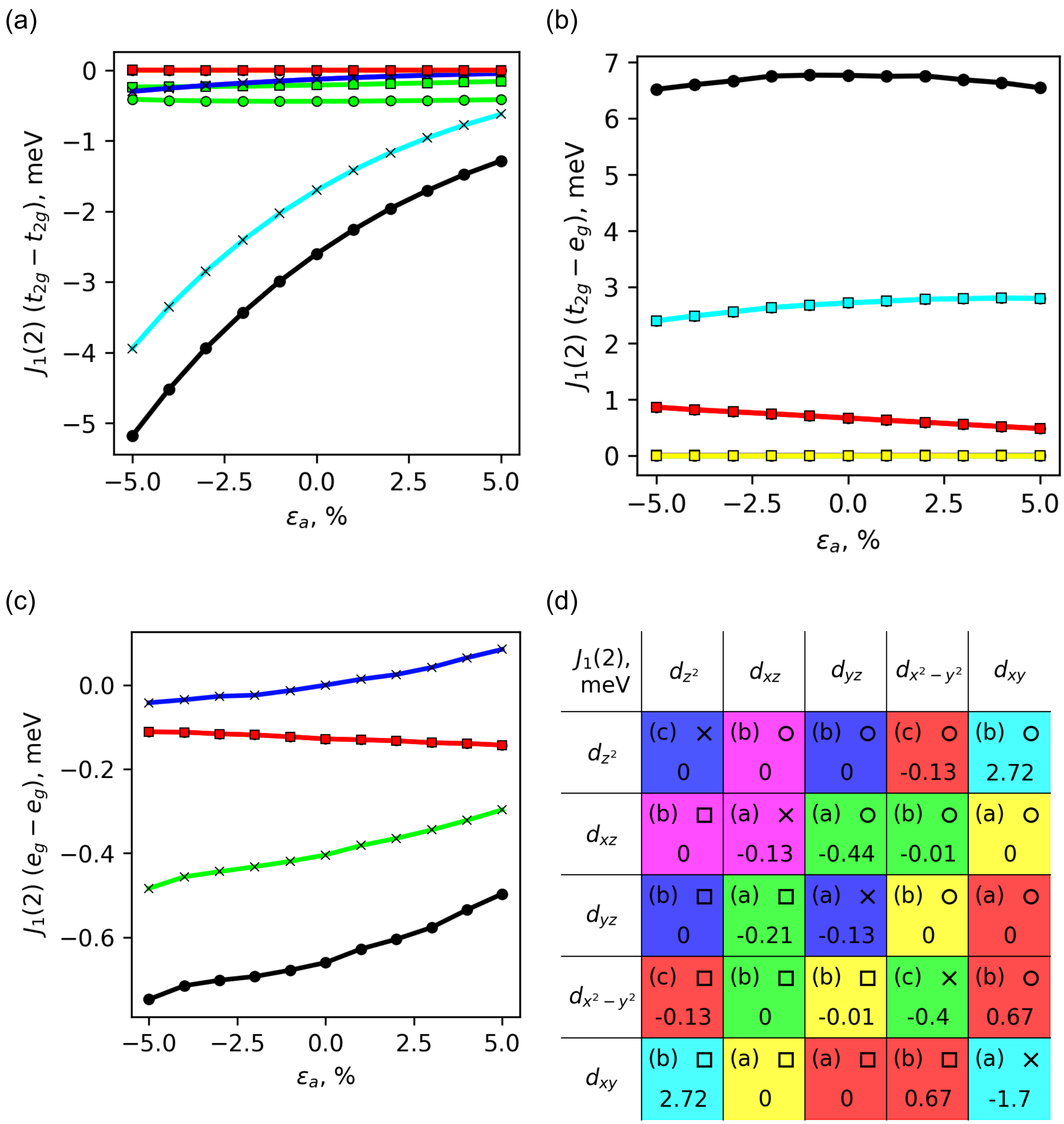

$J_1(2)$, meV	d_{z^2}	d_{xz}	d_{yz}	$d_{x^2-y^2}$	d_{xy}
d_{z^2}	(c) × 0	(b) ○ 0	(b) ○ 0	(c) ○ -0.13	(b) ○ 2.72
d_{xz}	(b) □ 0	(a) × -0.13	(a) ○ -0.44	(b) ○ -0.01	(a) ○ 0
d_{yz}	(b) □ 0	(a) □ -0.21	(a) × -0.13	(b) ○ 0	(a) ○ 0
$d_{x^2-y^2}$	(c) □ -0.13	(b) □ 0	(b) □ -0.01	(c) × -0.4	(b) ○ 0.67
d_{xy}	(b) □ 2.72	(a) □ 0	(a) □ 0	(b) □ 0.67	(a) × -1.7

Figure S21. Orbital-resolved isotropic exchange parameter $J_1(2)$ with applied uniaxial strain along *a*. (a) t_{2g}-t_{2g}; (b) t_{2g}-e_g; (c) e_g-e_g; (d) Color code for the graphs and values for $\varepsilon_a = 0\%$. Black color corresponds to the total exchange of each mechanism (t_{2g}-t_{2g}, t_{2g}-e_g or e_g-e_g).

ε_a = -5%

$J_1(2)$, meV	d_{z^2}	d_{xz}	d_{yz}	$d_{x^2-y^2}$	d_{xy}
d_{z^2}	-0.04	0	0	-0.11	2.4
d_{xz}	0	-0.3	-0.41	0	0
d_{yz}	0	-0.24	-0.3	0	0
$d_{x^2-y^2}$	-0.11	0	0	-0.48	0.86
d_{xy}	2.4	0	0	0.86	-3.94

ε_a = -4%

$J_1(2)$, meV	d_{z^2}	d_{xz}	d_{yz}	$d_{x^2-y^2}$	d_{xy}
d_{z^2}	-0.04	0	0	-0.11	2.48
d_{xz}	0	-0.25	-0.43	-0.01	0
d_{yz}	0	-0.23	-0.25	0	0
$d_{x^2-y^2}$	-0.11	0	-0.01	-0.46	0.82
d_{xy}	2.48	0	0	0.82	-3.35

ε_a = -3%

$J_1(2)$, meV	d_{z^2}	d_{xz}	d_{yz}	$d_{x^2-y^2}$	d_{xy}
d_{z^2}	-0.03	0	0	-0.12	2.56
d_{xz}	0	-0.21	-0.43	-0.01	0
d_{yz}	0	-0.23	-0.21	0	0
$d_{x^2-y^2}$	-0.12	0	-0.01	-0.44	0.78
d_{xy}	2.56	0	0	0.78	-2.85

ε_a = -2%

$J_1(2)$, meV	d_{z^2}	d_{xz}	d_{yz}	$d_{x^2-y^2}$	d_{xy}
d_{z^2}	-0.02	0	0	-0.12	2.63
d_{xz}	0	-0.18	-0.44	-0.01	0
d_{yz}	0	-0.23	-0.18	0	0
$d_{x^2-y^2}$	-0.12	0	-0.01	-0.43	0.74
d_{xy}	2.63	0	0	0.74	-2.41

ε_a = -1%

$J_1(2)$, meV	d_{z^2}	d_{xz}	d_{yz}	$d_{x^2-y^2}$	d_{xy}
d_{z^2}	-0.01	0	0	-0.12	2.68
d_{xz}	0	-0.15	-0.44	-0.01	0
d_{yz}	0	-0.22	-0.15	0	0
$d_{x^2-y^2}$	-0.12	0	-0.01	-0.42	0.71
d_{xy}	2.68	0	0	0.71	-2.03

ε_a = 0%

$J_1(2)$, meV	d_{z^2}	d_{xz}	d_{yz}	$d_{x^2-y^2}$	d_{xy}
d_{z^2}	0	0	0	-0.13	2.72
d_{xz}	0	-0.13	-0.44	-0.01	0
d_{yz}	0	-0.21	-0.13	0	0
$d_{x^2-y^2}$	-0.13	0	-0.01	-0.4	0.67
d_{xy}	2.72	0	0	0.67	-1.7

ε_a = 1%

$J_1(2)$, meV	d_{z^2}	d_{xz}	d_{yz}	$d_{x^2-y^2}$	d_{xy}
d_{z^2}	0.01	0	0	-0.13	2.75
d_{xz}	0	-0.1	-0.44	-0.01	0
d_{yz}	0	-0.2	-0.1	0	0
$d_{x^2-y^2}$	-0.13	0	-0.01	-0.38	0.63
d_{xy}	2.75	0	0	0.63	-1.41

ε_a = 2%

$J_1(2)$, meV	d_{z^2}	d_{xz}	d_{yz}	$d_{x^2-y^2}$	d_{xy}
d_{z^2}	0.02	0	0.01	-0.13	2.78
d_{xz}	0.01	-0.08	-0.43	0	0
d_{yz}	0	-0.19	-0.08	0	0
$d_{x^2-y^2}$	-0.13	0	0	-0.36	0.59
d_{xy}	2.78	0	0	0.59	-1.17

ε_a = 3%

$J_1(2)$, meV	d_{z^2}	d_{xz}	d_{yz}	$d_{x^2-y^2}$	d_{xy}
d_{z^2}	0.04	0	0	-0.14	2.79
d_{xz}	0	-0.07	-0.43	-0.01	0
d_{yz}	0	-0.18	-0.07	0	0
$d_{x^2-y^2}$	-0.14	0	-0.01	-0.34	0.55
d_{xy}	2.79	0	0	0.55	-0.96

ε_a = 4%

$J_1(2)$, meV	d_{z^2}	d_{xz}	d_{yz}	$d_{x^2-y^2}$	d_{xy}
d_{z^2}	0.06	0	0	-0.14	2.8
d_{xz}	0	-0.06	-0.42	-0.01	0
d_{yz}	0	-0.17	-0.06	0	0
$d_{x^2-y^2}$	-0.14	0	-0.01	-0.32	0.52
d_{xy}	2.8	0	0	0.52	-0.78

ε_a = 5%

$J_1(2)$, meV	d_{z^2}	d_{xz}	d_{yz}	$d_{x^2-y^2}$	d_{xy}
d_{z^2}	0.08	0	0	-0.14	2.79
d_{xz}	0	-0.04	-0.42	-0.01	0
d_{yz}	0	-0.16	-0.04	0	0
$d_{x^2-y^2}$	-0.14	0	-0.01	-0.3	0.48
d_{xy}	2.79	0	0	0.48	-0.62

Color code:
t_{2g}-t_{2g}: blue
t_{2g}-e_g: orange
e_g-e_g: green

Figure S22. Orbital-resolved isotropic exchange parameter $J_1(2)$ with applied uniaxial strain along *a*.

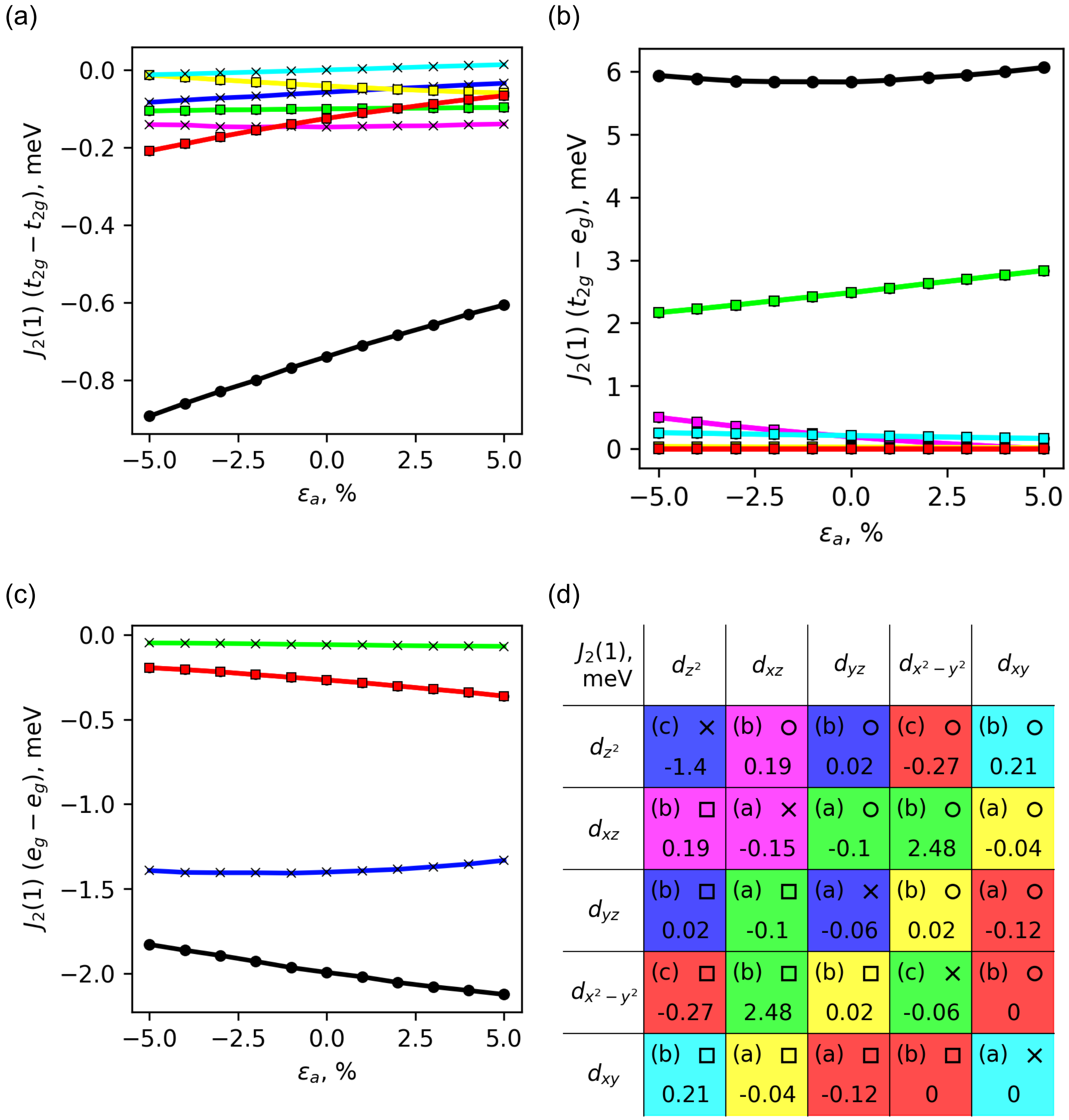

$J_2(1)$, meV	d_{z^2}	d_{xz}	d_{yz}	$d_{x^2-y^2}$	d_{xy}
d_{z^2}	(c) × -1.4	(b) ○ 0.19	(b) ○ 0.02	(c) ○ -0.27	(b) ○ 0.21
d_{xz}	(b) □ 0.19	(a) × -0.15	(a) ○ -0.1	(b) ○ 2.48	(a) ○ -0.04
d_{yz}	(b) □ 0.02	(a) □ -0.1	(a) × -0.06	(b) ○ 0.02	(a) ○ -0.12
$d_{x^2-y^2}$	(c) □ -0.27	(b) □ 2.48	(b) □ 0.02	(c) × -0.06	(b) ○ 0
d_{xy}	(b) □ 0.21	(a) □ -0.04	(a) □ -0.12	(b) □ 0	(a) × 0

Figure S23. Orbital-resolved isotropic exchange parameter $J_2(1)$ with applied uniaxial strain along *a*. (a) t_{2g}-t_{2g}; (b) t_{2g}-e_g; (c) e_g-e_g; (d) Color code for the graphs and values for ε_a = 0%. Black color corresponds to the total exchange of each mechanism (t_{2g}-t_{2g}, t_{2g}-e_g or e_g-e_g).

εa = -5%

$J_2(1)$, meV	d_{z^2}	d_{xz}	d_{yz}	$d_{x^2-y^2}$	d_{xy}
d_{z^2}	-1.39	0.5	0.02	-0.19	0.26
d_{xz}	0.5	-0.14	-0.11	2.17	-0.01
d_{yz}	0.02	-0.11	-0.08	0.03	-0.21
$d_{x^2-y^2}$	-0.19	2.17	0.03	-0.05	0
d_{xy}	0.26	-0.01	-0.21	0	-0.01

εa = -4%

$J_2(1)$, meV	d_{z^2}	d_{xz}	d_{yz}	$d_{x^2-y^2}$	d_{xy}
d_{z^2}	-1.4	0.43	0.02	-0.21	0.25
d_{xz}	0.43	-0.14	-0.1	2.22	-0.02
d_{yz}	0.02	-0.1	-0.08	0.03	-0.19
$d_{x^2-y^2}$	-0.21	2.22	0.03	-0.05	0
d_{xy}	0.25	-0.02	-0.19	0	-0.01

εa = -3%

$J_2(1)$, meV	d_{z^2}	d_{xz}	d_{yz}	$d_{x^2-y^2}$	d_{xy}
d_{z^2}	-1.41	0.36	0.02	-0.22	0.24
d_{xz}	0.36	-0.15	-0.1	2.29	-0.03
d_{yz}	0.02	-0.1	-0.07	0.03	-0.17
$d_{x^2-y^2}$	-0.22	2.29	0.03	-0.05	0
d_{xy}	0.24	-0.03	-0.17	0	-0.01

εa = -2%

$J_2(1)$, meV	d_{z^2}	d_{xz}	d_{yz}	$d_{x^2-y^2}$	d_{xy}
d_{z^2}	-1.41	0.3	0.02	-0.24	0.23
d_{xz}	0.3	-0.15	-0.1	2.35	-0.03
d_{yz}	0.02	-0.1	-0.07	0.03	-0.16
$d_{x^2-y^2}$	-0.24	2.35	0.03	-0.05	0
d_{xy}	0.23	-0.03	-0.16	0	-0.01

εa = -1%

$J_2(1)$, meV	d_{z^2}	d_{xz}	d_{yz}	$d_{x^2-y^2}$	d_{xy}
d_{z^2}	-1.41	0.24	0.02	-0.25	0.22
d_{xz}	0.24	-0.15	-0.1	2.42	-0.04
d_{yz}	0.02	-0.1	-0.06	0.03	-0.14
$d_{x^2-y^2}$	-0.25	2.42	0.03	-0.06	0
d_{xy}	0.22	-0.04	-0.14	0	0

εa = 0%

$J_2(1)$, meV	d_{z^2}	d_{xz}	d_{yz}	$d_{x^2-y^2}$	d_{xy}
d_{z^2}	-1.4	0.19	0.02	-0.27	0.21
d_{xz}	0.19	-0.15	-0.1	2.48	-0.04
d_{yz}	0.02	-0.1	-0.06	0.02	-0.12
$d_{x^2-y^2}$	-0.27	2.48	0.02	-0.06	0
d_{xy}	0.21	-0.04	-0.12	0	0

εa = 1%

$J_2(1)$, meV	d_{z^2}	d_{xz}	d_{yz}	$d_{x^2-y^2}$	d_{xy}
d_{z^2}	-1.39	0.14	0.02	-0.28	0.2
d_{xz}	0.14	-0.15	-0.1	2.56	-0.05
d_{yz}	0.02	-0.1	-0.05	0.02	-0.11
$d_{x^2-y^2}$	-0.28	2.56	0.02	-0.06	0
d_{xy}	0.2	-0.05	-0.11	0	0

εa = 2%

$J_2(1)$, meV	d_{z^2}	d_{xz}	d_{yz}	$d_{x^2-y^2}$	d_{xy}
d_{z^2}	-1.39	0.1	0.02	-0.3	0.19
d_{xz}	0.1	-0.14	-0.1	2.63	-0.05
d_{yz}	0.02	-0.1	-0.05	0.02	-0.1
$d_{x^2-y^2}$	-0.3	2.63	0.02	-0.06	0
d_{xy}	0.19	-0.05	-0.1	0	0.01

εa = 3%

$J_2(1)$, meV	d_{z^2}	d_{xz}	d_{yz}	$d_{x^2-y^2}$	d_{xy}
d_{z^2}	-1.37	0.06	0.02	-0.32	0.18
d_{xz}	0.06	-0.14	-0.1	2.7	-0.05
d_{yz}	0.02	-0.1	-0.04	0.02	-0.09
$d_{x^2-y^2}$	-0.32	2.7	0.02	-0.07	0
d_{xy}	0.18	-0.05	-0.09	0	0.01

εa = 4%

$J_2(1)$, meV	d_{z^2}	d_{xz}	d_{yz}	$d_{x^2-y^2}$	d_{xy}
d_{z^2}	-1.35	0.03	0.02	-0.34	0.17
d_{xz}	0.03	-0.14	-0.1	2.77	-0.06
d_{yz}	0.02	-0.1	-0.04	0.02	-0.08
$d_{x^2-y^2}$	-0.34	2.77	0.02	-0.07	0
d_{xy}	0.17	-0.06	-0.08	0	0.01

εa = 5%

$J_2(1)$, meV	d_{z^2}	d_{xz}	d_{yz}	$d_{x^2-y^2}$	d_{xy}
d_{z^2}	-1.33	0	0.02	-0.36	0.16
d_{xz}	0	-0.14	-0.1	2.84	-0.06
d_{yz}	0.02	-0.1	-0.03	0.02	-0.07
$d_{x^2-y^2}$	-0.36	2.84	0.02	-0.07	0
d_{xy}	0.16	-0.06	-0.07	0	0.01

Color code:
t_{2g}-t_{2g}: blue
t_{2g}-e_g: orange
e_g-e_g: green

Figure S24. Orbital-resolved isotropic exchange parameter $J_2(1)$ with applied uniaxial strain along *a*.

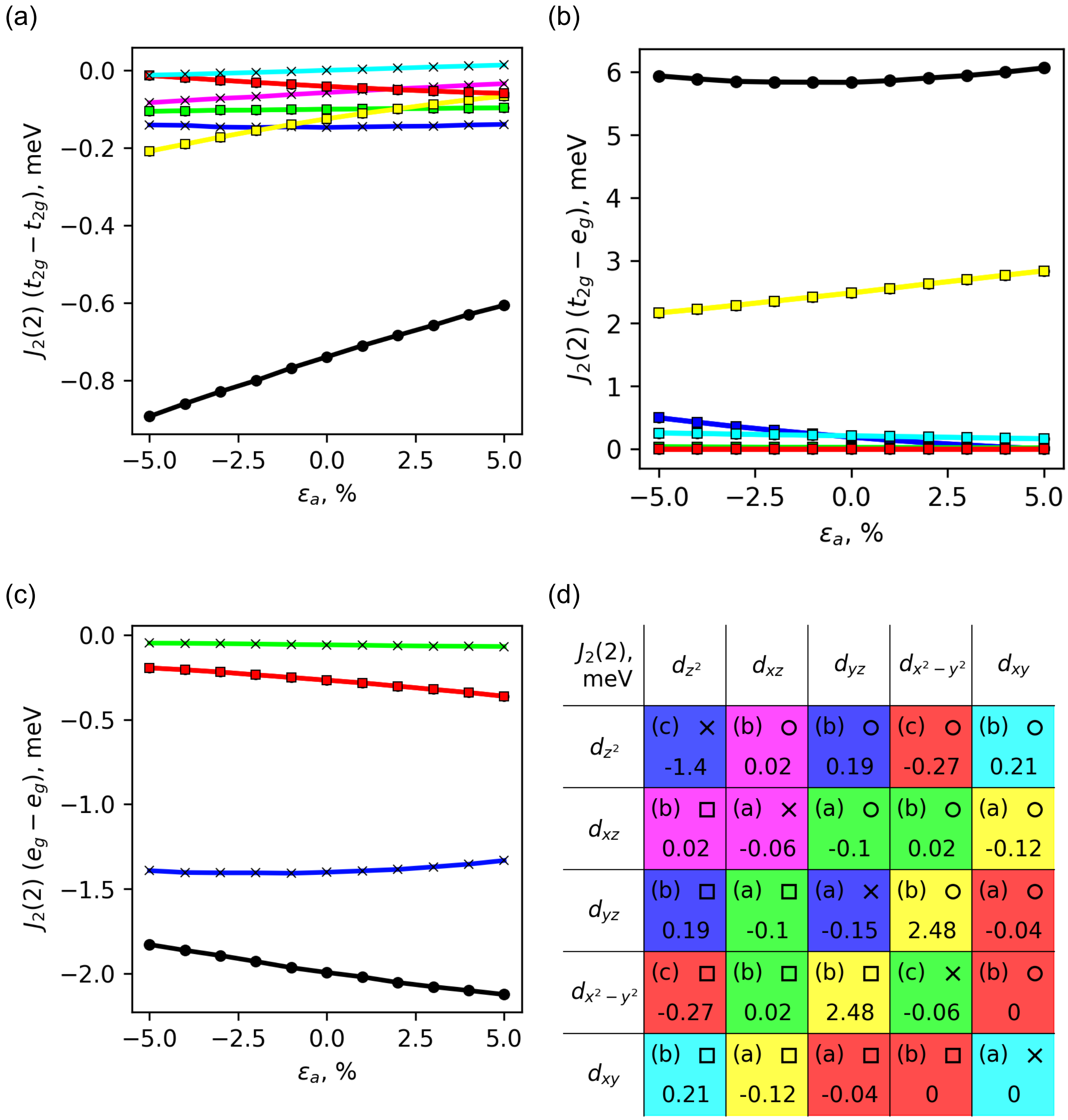

$J_2(2)$, meV	d_{z^2}	d_{xz}	d_{yz}	$d_{x^2-y^2}$	d_{xy}
d_{z^2}	(c) × -1.4	(b) ○ 0.02	(b) ○ 0.19	(c) ○ -0.27	(b) ○ 0.21
d_{xz}	(b) □ 0.02	(a) × -0.06	(a) ○ -0.1	(b) ○ 0.02	(a) ○ -0.12
d_{yz}	(b) □ 0.19	(a) □ -0.1	(a) × -0.15	(b) ○ 2.48	(a) ○ -0.04
$d_{x^2-y^2}$	(c) □ -0.27	(b) □ 0.02	(b) □ 2.48	(c) × -0.06	(b) ○ 0
d_{xy}	(b) □ 0.21	(a) □ -0.12	(a) □ -0.04	(b) □ 0	(a) × 0

Figure S25. Orbital-resolved isotropic exchange parameter $J_2(2)$ with applied uniaxial strain along *a*. (a) t_{2g}-t_{2g}; (b) t_{2g}-e_g; (c) e_g-e_g; (d) Color code for the graphs and values for ε_a = 0%. Black color corresponds to the total exchange of each mechanism (t_{2g}-t_{2g}, t_{2g}-e_g or e_g-e_g).

ε_a = -5%

$J_2(2)$, meV	d_{z^2}	d_{xz}	d_{yz}	$d_{x^2-y^2}$	d_{xy}
d_{z^2}	-1.39	0.02	0.5	-0.19	0.26
d_{xz}	0.02	-0.08	-0.11	0.03	-0.21
d_{yz}	0.5	-0.11	-0.14	2.17	-0.01
$d_{x^2-y^2}$	-0.19	0.03	2.17	-0.05	0
d_{xy}	0.26	-0.21	-0.01	0	-0.01

ε_a = -4%

$J_2(2)$, meV	d_{z^2}	d_{xz}	d_{yz}	$d_{x^2-y^2}$	d_{xy}
d_{z^2}	-1.4	0.02	0.43	-0.21	0.25
d_{xz}	0.02	-0.08	-0.1	0.03	-0.19
d_{yz}	0.43	-0.1	-0.14	2.22	-0.02
$d_{x^2-y^2}$	-0.21	0.03	2.22	-0.05	0
d_{xy}	0.25	-0.19	-0.02	0	-0.01

ε_a = -3%

$J_2(2)$, meV	d_{z^2}	d_{xz}	d_{yz}	$d_{x^2-y^2}$	d_{xy}
d_{z^2}	-1.41	0.02	0.36	-0.22	0.24
d_{xz}	0.02	-0.07	-0.1	0.03	-0.17
d_{yz}	0.36	-0.1	-0.15	2.29	-0.03
$d_{x^2-y^2}$	-0.22	0.03	2.29	-0.05	0
d_{xy}	0.24	-0.17	-0.03	0	-0.01

ε_a = -2%

$J_2(2)$, meV	d_{z^2}	d_{xz}	d_{yz}	$d_{x^2-y^2}$	d_{xy}
d_{z^2}	-1.41	0.02	0.3	-0.24	0.23
d_{xz}	0.02	-0.07	-0.1	0.03	-0.16
d_{yz}	0.3	-0.1	-0.15	2.35	-0.03
$d_{x^2-y^2}$	-0.24	0.03	2.35	-0.05	0
d_{xy}	0.23	-0.16	-0.03	0	-0.01

ε_a = -1%

$J_2(2)$, meV	d_{z^2}	d_{xz}	d_{yz}	$d_{x^2-y^2}$	d_{xy}
d_{z^2}	-1.41	0.02	0.24	-0.25	0.22
d_{xz}	0.02	-0.06	-0.1	0.03	-0.14
d_{yz}	0.24	-0.1	-0.15	2.42	-0.04
$d_{x^2-y^2}$	-0.25	0.03	2.42	-0.06	0
d_{xy}	0.22	-0.14	-0.04	0	0

ε_a = 0%

$J_2(2)$, meV	d_{z^2}	d_{xz}	d_{yz}	$d_{x^2-y^2}$	d_{xy}
d_{z^2}	-1.4	0.02	0.19	-0.27	0.21
d_{xz}	0.02	-0.06	-0.1	0.02	-0.12
d_{yz}	0.19	-0.1	-0.15	2.48	-0.04
$d_{x^2-y^2}$	-0.27	0.02	2.48	-0.06	0
d_{xy}	0.21	-0.12	-0.04	0	0

ε_a = 1%

$J_2(2)$, meV	d_{z^2}	d_{xz}	d_{yz}	$d_{x^2-y^2}$	d_{xy}
d_{z^2}	-1.39	0.02	0.14	-0.28	0.2
d_{xz}	0.02	-0.05	-0.1	0.02	-0.11
d_{yz}	0.14	-0.1	-0.15	2.56	-0.05
$d_{x^2-y^2}$	-0.28	0.02	2.56	-0.06	0
d_{xy}	0.2	-0.11	-0.05	0	0

ε_a = 2%

$J_2(2)$, meV	d_{z^2}	d_{xz}	d_{yz}	$d_{x^2-y^2}$	d_{xy}
d_{z^2}	-1.39	0.02	0.1	-0.3	0.19
d_{xz}	0.02	-0.05	-0.1	0.02	-0.1
d_{yz}	0.1	-0.1	-0.14	2.63	-0.05
$d_{x^2-y^2}$	-0.3	0.02	2.63	-0.06	0
d_{xy}	0.19	-0.1	-0.05	0	0.01

ε_a = 3%

$J_2(2)$, meV	d_{z^2}	d_{xz}	d_{yz}	$d_{x^2-y^2}$	d_{xy}
d_{z^2}	-1.37	0.02	0.06	-0.32	0.18
d_{xz}	0.02	-0.04	-0.1	0.02	-0.09
d_{yz}	0.06	-0.1	-0.14	2.7	-0.05
$d_{x^2-y^2}$	-0.32	0.02	2.7	-0.07	0
d_{xy}	0.18	-0.09	-0.05	0	0.01

ε_a = 4%

$J_2(2)$, meV	d_{z^2}	d_{xz}	d_{yz}	$d_{x^2-y^2}$	d_{xy}
d_{z^2}	-1.35	0.02	0.03	-0.34	0.17
d_{xz}	0.02	-0.04	-0.1	0.02	-0.08
d_{yz}	0.03	-0.1	-0.14	2.77	-0.06
$d_{x^2-y^2}$	-0.34	0.02	2.77	-0.07	0
d_{xy}	0.17	-0.08	-0.06	0	0.01

ε_a = 5%

$J_2(2)$, meV	d_{z^2}	d_{xz}	d_{yz}	$d_{x^2-y^2}$	d_{xy}
d_{z^2}	-1.33	0.02	0	-0.36	0.16
d_{xz}	0.02	-0.03	-0.1	0.02	-0.07
d_{yz}	0	-0.1	-0.14	2.84	-0.06
$d_{x^2-y^2}$	-0.36	0.02	2.84	-0.07	0
d_{xy}	0.16	-0.07	-0.06	0	0.01

Color code:
t_{2g}-t_{2g}: blue
t_{2g}-e_g: orange
e_g-e_g: green

Figure S26. Orbital-resolved isotropic exchange parameter $J_2(2)$ with applied uniaxial strain along *a*.

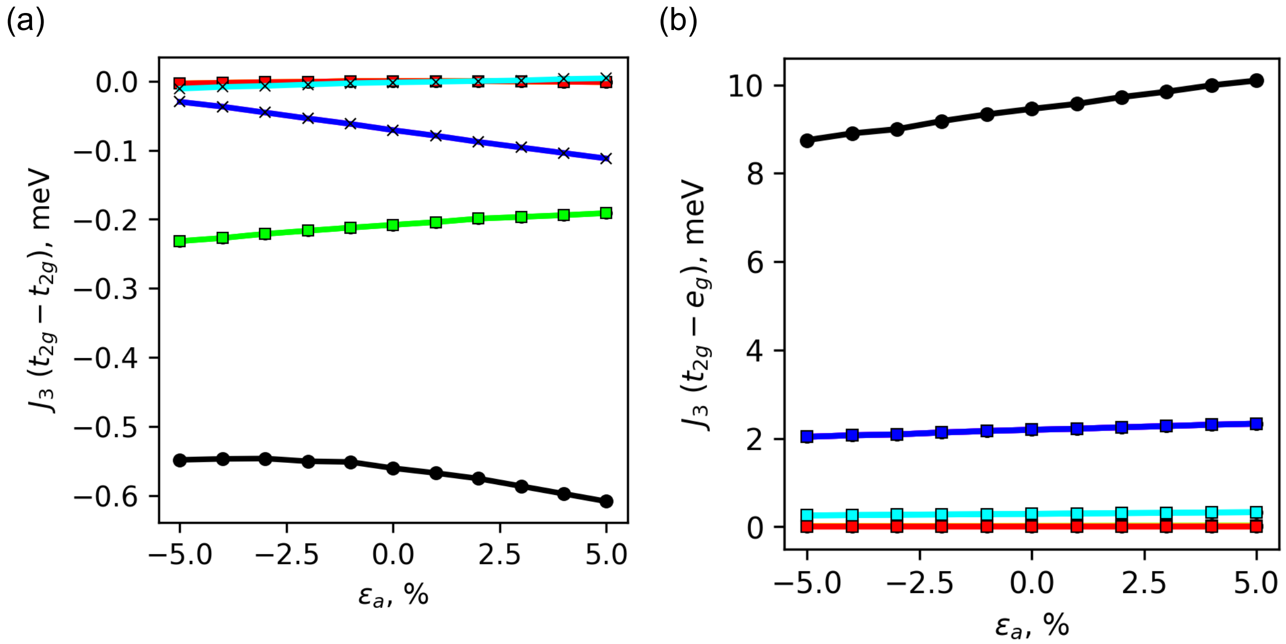

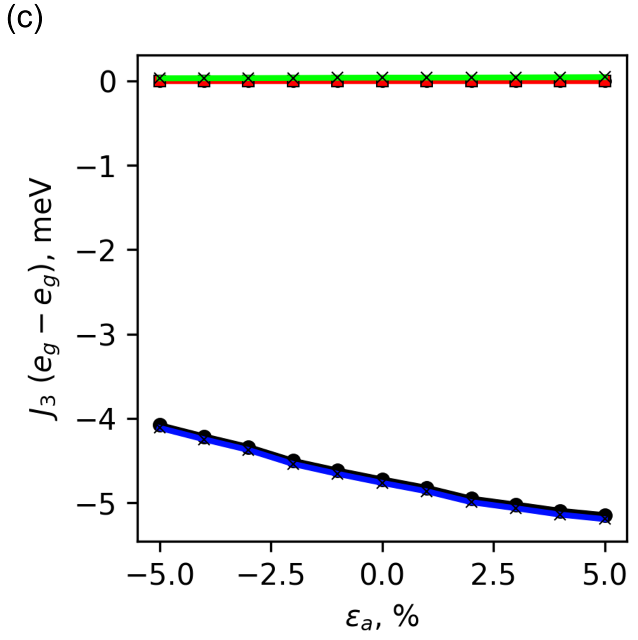

(d)

J_3, meV	d_{z^2}	d_{xz}	d_{yz}	$d_{x^2-y^2}$	d_{xy}
d_{z^2}	(c) × -4.77	(b) ○ 2.2	(b) ○ 2.2	(c) ○ 0	(b) ○ 0.29
d_{xz}	(b) □ 2.2	(a) × -0.07	(a) ○ -0.21	(b) ○ 0.02	(a) ○ 0
d_{yz}	(b) □ 2.2	(a) □ -0.21	(a) × -0.07	(b) ○ 0.02	(a) ○ 0
$d_{x^2-y^2}$	(c) □ 0	(b) □ 0.02	(b) □ 0.02	(c) × 0.04	(b) ○ 0
d_{xy}	(b) □ 0.29	(a) □ 0	(a) □ 0	(b) □ 0	(a) × 0

Figure S27. Orbital-resolved isotropic exchange parameter J_3 with applied uniaxial strain along *a*. (a) t_{2g}-t_{2g}; (b) t_{2g}-e_g; (c) e_g-e_g; (d) Color code for the graphs and values for $\varepsilon_a = 0\%$. Black color corresponds to the total exchange of each mechanism (t_{2g}-t_{2g}, t_{2g}-e_g or e_g-e_g).

ε_a = -5%

J_3, meV	d_{z^2}	d_{xz}	d_{yz}	$d_{x^2-y^2}$	d_{xy}
d_{z^2}	-4.11	2.04	2.04	0	0.26
d_{xz}	2.04	-0.03	-0.23	0.02	0
d_{yz}	2.04	-0.23	-0.03	0.02	0
$d_{x^2-y^2}$	0	0.02	0.02	0.03	0
d_{xy}	0.26	0	0	0	-0.01

ε_a = -4%

J_3, meV	d_{z^2}	d_{xz}	d_{yz}	$d_{x^2-y^2}$	d_{xy}
d_{z^2}	-4.25	2.07	2.07	0	0.27
d_{xz}	2.07	-0.04	-0.23	0.02	0
d_{yz}	2.07	-0.23	-0.04	0.02	0
$d_{x^2-y^2}$	0	0.02	0.02	0.03	0
d_{xy}	0.27	0	0	0	-0.01

ε_a = -3%

J_3, meV	d_{z^2}	d_{xz}	d_{yz}	$d_{x^2-y^2}$	d_{xy}
d_{z^2}	-4.38	2.09	2.09	0	0.27
d_{xz}	2.09	-0.05	-0.22	0.02	0
d_{yz}	2.09	-0.22	-0.05	0.02	0
$d_{x^2-y^2}$	0	0.02	0.02	0.04	0
d_{xy}	0.27	0	0	0	-0.01

ε_a = -2%

J_3, meV	d_{z^2}	d_{xz}	d_{yz}	$d_{x^2-y^2}$	d_{xy}
d_{z^2}	-4.54	2.13	2.13	0	0.28
d_{xz}	2.13	-0.05	-0.22	0.02	0
d_{yz}	2.13	-0.22	-0.05	0.02	0
$d_{x^2-y^2}$	0	0.02	0.02	0.04	0
d_{xy}	0.28	0	0	0	0

ε_a = -1%

J_3, meV	d_{z^2}	d_{xz}	d_{yz}	$d_{x^2-y^2}$	d_{xy}
d_{z^2}	-4.66	2.17	2.17	0	0.29
d_{xz}	2.17	-0.06	-0.21	0.02	0
d_{yz}	2.17	-0.21	-0.06	0.02	0
$d_{x^2-y^2}$	0	0.02	0.02	0.04	0
d_{xy}	0.29	0	0	0	0

ε_a = 0%

J_3, meV	d_{z^2}	d_{xz}	d_{yz}	$d_{x^2-y^2}$	d_{xy}
d_{z^2}	-4.77	2.2	2.2	0	0.29
d_{xz}	2.2	-0.07	-0.21	0.02	0
d_{yz}	2.2	-0.21	-0.07	0.02	0
$d_{x^2-y^2}$	0	0.02	0.02	0.04	0
d_{xy}	0.29	0	0	0	0

ε_a = 1%

J_3, meV	d_{z^2}	d_{xz}	d_{yz}	$d_{x^2-y^2}$	d_{xy}
d_{z^2}	-4.87	2.22	2.22	0	0.3
d_{xz}	2.22	-0.08	-0.2	0.02	0
d_{yz}	2.22	-0.2	-0.08	0.02	0
$d_{x^2-y^2}$	0	0.02	0.02	0.04	0
d_{xy}	0.3	0	0	0	0

ε_a = 2%

J_3, meV	d_{z^2}	d_{xz}	d_{yz}	$d_{x^2-y^2}$	d_{xy}
d_{z^2}	-5.0	2.25	2.25	0	0.31
d_{xz}	2.25	-0.09	-0.2	0.02	0
d_{yz}	2.25	-0.2	-0.09	0.02	0
$d_{x^2-y^2}$	0	0.02	0.02	0.04	0
d_{xy}	0.31	0	0	0	0

ε_a = 3%

J_3, meV	d_{z^2}	d_{xz}	d_{yz}	$d_{x^2-y^2}$	d_{xy}
d_{z^2}	-5.07	2.28	2.28	0	0.32
d_{xz}	2.28	-0.1	-0.2	0.02	0
d_{yz}	2.28	-0.2	-0.1	0.02	0
$d_{x^2-y^2}$	0	0.02	0.02	0.04	0
d_{xy}	0.32	0	0	0	0

ε_a = 4%

J_3, meV	d_{z^2}	d_{xz}	d_{yz}	$d_{x^2-y^2}$	d_{xy}
d_{z^2}	-5.14	2.31	2.31	0	0.32
d_{xz}	2.31	-0.1	-0.19	0.02	0
d_{yz}	2.31	-0.19	-0.1	0.02	0
$d_{x^2-y^2}$	0	0.02	0.02	0.04	0
d_{xy}	0.32	0	0	0	0

ε_a = 5%

J_3, meV	d_{z^2}	d_{xz}	d_{yz}	$d_{x^2-y^2}$	d_{xy}
d_{z^2}	-5.2	2.33	2.33	0	0.33
d_{xz}	2.33	-0.11	-0.19	0.02	0
d_{yz}	2.33	-0.19	-0.11	0.02	0
$d_{x^2-y^2}$	0	0.02	0.02	0.05	0
d_{xy}	0.33	0	0	0	0

Color code:
t_{2g}-t_{2g}: blue
t_{2g}-e_g: orange
e_g-e_g: green

Figure S28. Orbital-resolved isotropic exchange parameter J_3 with applied uniaxial strain along *a*.

3.4 Magnetic moment per atom vs strain applied along *a* (U = 3 eV)

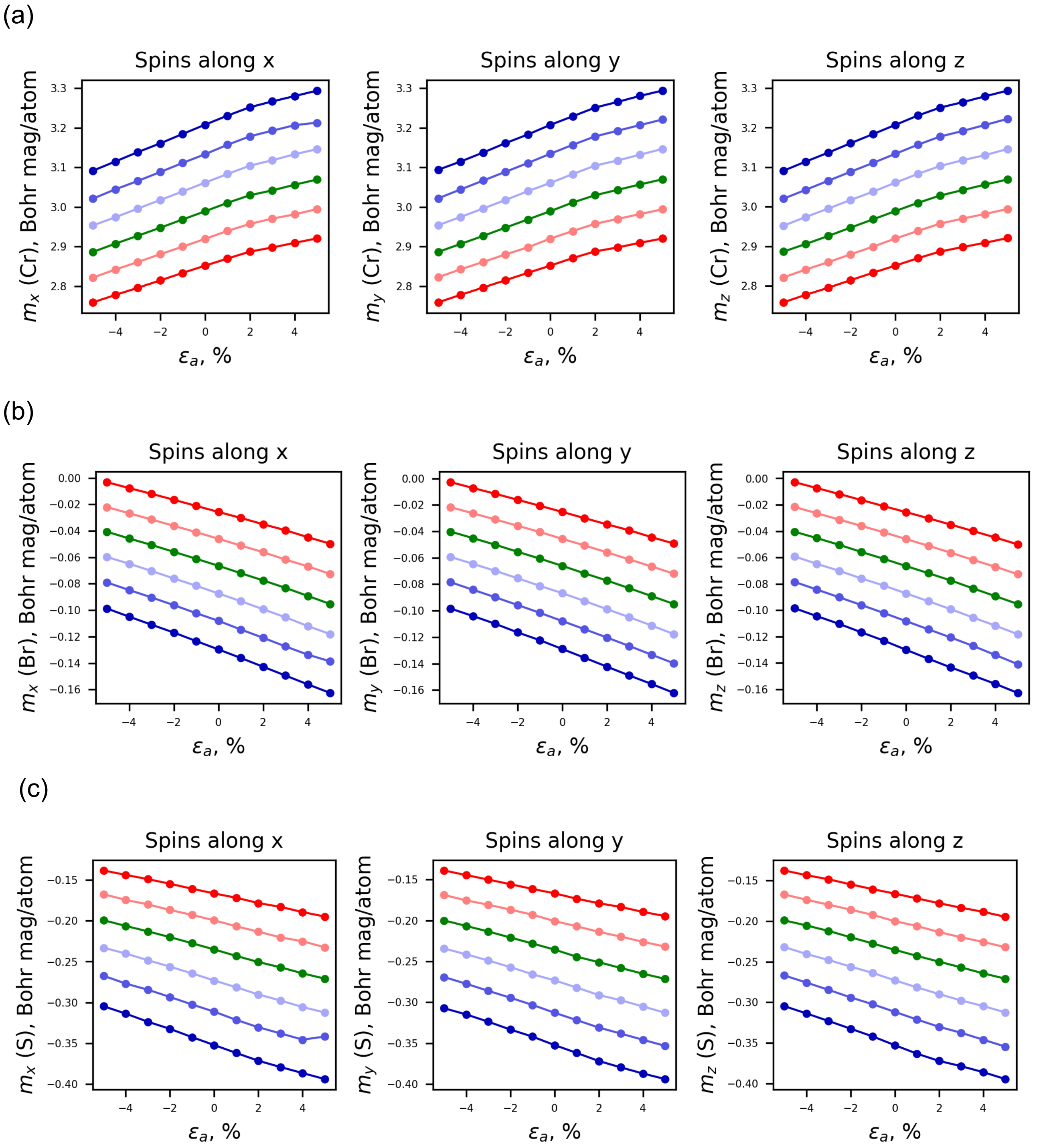

Figure S29. Magnetic moment per (a) Cr, (b) Br (c) S atom for strain applied along *a* for each type of strain three DFT calculations are presented with alignment of spins along x, y or z Cartesian axis (specified in the pictures). Colors from red to blue corresponds to the values of Hubbard U - from 1 eV to 6 eV (with the step of 1 eV, i.e., green corresponds to 3 eV)

3.5 Isotropic exchange vs strain applied along *b* (U = 3 eV)

Table S5. Orbital resolved isotropic exchange parameters with strain applied along *b*.

	J_1, meV			J_2, meV			J_3, meV		
ε_b	t_{2g}-t_{2g}	t_{2g}-e_g	e_g-e_g	t_{2g}-t_{2g}	t_{2g}-e_g	e_g-e_g	t_{2g}-t_{2g}	t_{2g}-e_g	e_g-e_g
**-5%**	-2.63	6.57	-0.60	-0.79	7.38	-1.61	-1.28	10.74	-3.48
**-4%**	-2.63	6.64	-0.60	-0.79	6.95	-1.68	-1.10	10.54	-3.73
**-3%**	-2.62	6.66	-0.62	-0.78	6.59	-1.75	-0.93	10.25	-3.94
**-2%**	-2.62	6.70	-0.63	-0.77	6.29	-1.83	-0.79	9.98	-4.21
**-1%**	-2.61	6.73	-0.64	-0.76	6.05	-1.90	-0.67	9.71	-4.46
**0%**	-2.60	6.76	-0.66	-0.74	5.83	-1.99	-0.56	9.46	-4.73
**1%**	-2.59	6.81	-0.67	-0.72	5.70	-2.08	-0.47	9.17	-5.00
**2%**	-2.57	6.83	-0.68	-0.69	5.61	-2.17	-0.39	8.87	-5.26
**3%**	-2.55	6.84	-0.70	-0.66	5.56	-2.28	-0.32	8.60	-5.52
**4%**	-2.54	6.86	-0.71	-0.64	5.57	-2.39	-0.27	8.30	-5.79
**5%**	-2.51	6.87	-0.73	-0.61	5.60	-2.50	-0.22	7.98	-6.01

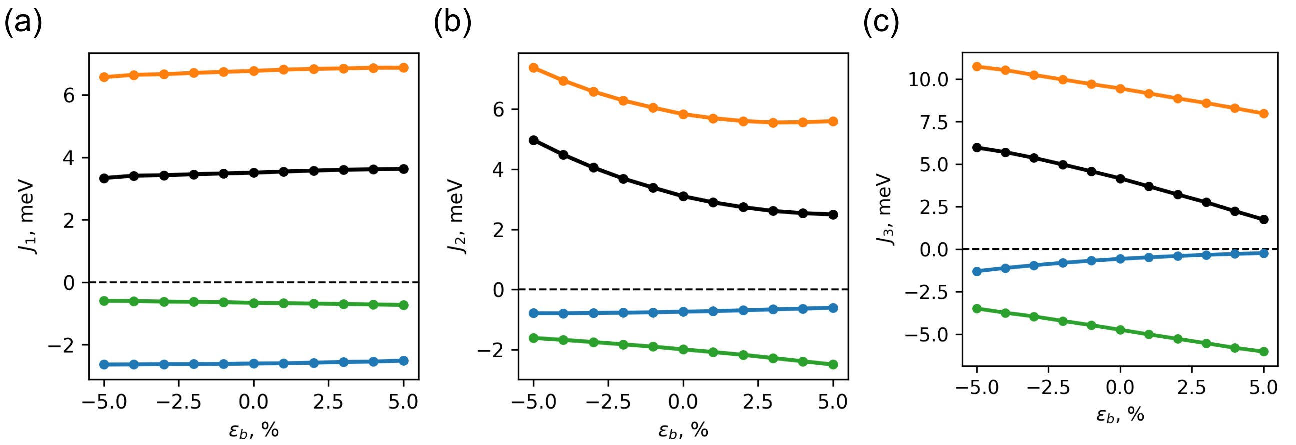

Figure S30. Orbital-resolved isotropic exchange parameters: (a) J_1; (b) J_2; (c) J_3, with strain applied along *b* axis. Black color corresponds to the total parameter J_i, green - to the e_g-e_g exchange channel, orange - to the t_{2g}-e_g exchange channel and blue - to the t_{2g}-t_{2g} exchange channel.

(a) (b)

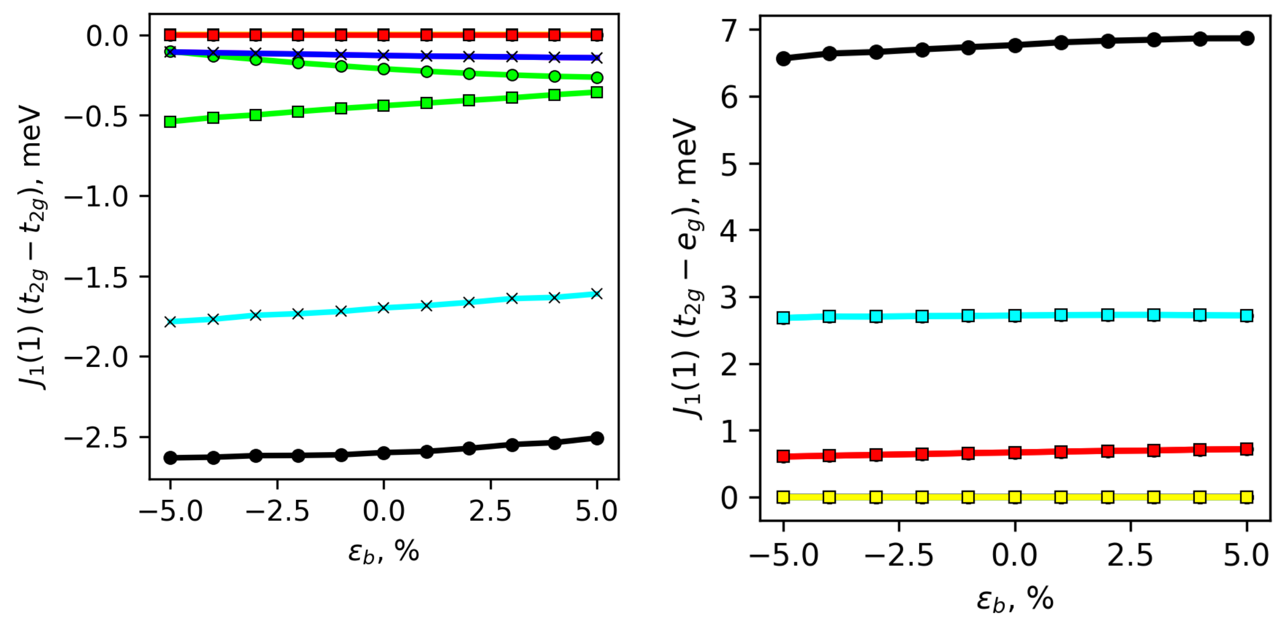

(c)

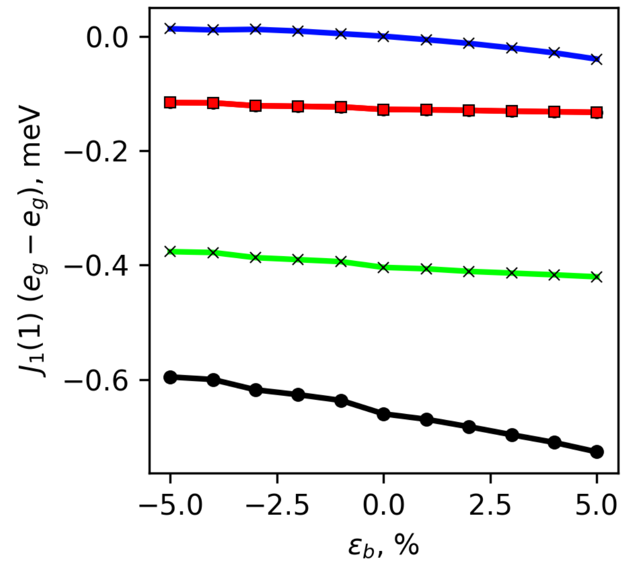

(d)

$J_1(1)$, meV	d_{z^2}	d_{xz}	d_{yz}	$d_{x^2-y^2}$	d_{xy}
d_{z^2}	(c) × 0	(b) ○ 0	(b) ○ 0	(c) ○ -0.13	(b) ○ 2.72
d_{xz}	(b) □ 0	(a) × -0.13	(a) ○ -0.21	(b) ○ 0	(a) ○ 0
d_{yz}	(b) □ 0	(a) □ -0.44	(a) × -0.13	(b) ○ -0.01	(a) ○ 0
$d_{x^2-y^2}$	(c) □ -0.13	(b) □ -0.01	(b) □ 0	(c) × -0.4	(b) ○ 0.67
d_{xy}	(b) □ 2.72	(a) □ 0	(a) □ 0	(b) □ 0.67	(a) × -1.7

Figure S31. Orbital-resolved isotropic exchange parameter $J_1(1)$ with applied uniaxial strain along *b*. (a) t_{2g}-t_{2g}; (b) t_{2g}-e_g; (c) e_g-e_g; (d) Color code for the graphs and values for ε_b = 0%. Black color corresponds to the total exchange of each mechanism (t_{2g}-t_{2g}, t_{2g}-e_g or e_g-e_g).

ε_b = -5%

$J_1(1)$, meV	d_{z^2}	d_{xz}	d_{yz}	$d_{x^2-y^2}$	d_{xy}
d_{z^2}	0.01	0	0	-0.12	2.68
d_{xz}	0	-0.1	-0.1	0	0
d_{yz}	0	-0.54	-0.1	-0.01	0
$d_{x^2-y^2}$	-0.12	-0.01	0	-0.38	0.6
d_{xy}	2.68	0	0	0.6	-1.78

ε_b = -4%

$J_1(1)$, meV	d_{z^2}	d_{xz}	d_{yz}	$d_{x^2-y^2}$	d_{xy}
d_{z^2}	0.01	0	0	-0.12	2.7
d_{xz}	0	-0.11	-0.13	0	0
d_{yz}	0	-0.51	-0.11	-0.01	0
$d_{x^2-y^2}$	-0.12	-0.01	0	-0.38	0.62
d_{xy}	2.7	0	0	0.62	-1.77

ε_b = -3%

$J_1(1)$, meV	d_{z^2}	d_{xz}	d_{yz}	$d_{x^2-y^2}$	d_{xy}
d_{z^2}	0.01	0	0	-0.12	2.7
d_{xz}	0	-0.11	-0.15	0	0
d_{yz}	0	-0.5	-0.11	-0.01	0
$d_{x^2-y^2}$	-0.12	-0.01	0	-0.39	0.63
d_{xy}	2.7	0	0	0.63	-1.74

ε_b = -2%

$J_1(1)$, meV	d_{z^2}	d_{xz}	d_{yz}	$d_{x^2-y^2}$	d_{xy}
d_{z^2}	0.01	0	0	-0.12	2.71
d_{xz}	0	-0.12	-0.17	0	0
d_{yz}	0	-0.48	-0.12	-0.01	0
$d_{x^2-y^2}$	-0.12	-0.01	0	-0.39	0.64
d_{xy}	2.71	0	0	0.64	-1.74

ε_b = -1%

$J_1(1)$, meV	d_{z^2}	d_{xz}	d_{yz}	$d_{x^2-y^2}$	d_{xy}
d_{z^2}	0	0	0	-0.12	2.71
d_{xz}	0	-0.12	-0.19	0	0
d_{yz}	0	-0.46	-0.12	-0.01	0
$d_{x^2-y^2}$	-0.12	-0.01	0	-0.39	0.66
d_{xy}	2.71	0	0	0.66	-1.72

ε_b = 0%

$J_1(1)$, meV	d_{z^2}	d_{xz}	d_{yz}	$d_{x^2-y^2}$	d_{xy}
d_{z^2}	0	0	0	-0.13	2.72
d_{xz}	0	-0.13	-0.21	0	0
d_{yz}	0	-0.44	-0.13	-0.01	0
$d_{x^2-y^2}$	-0.13	-0.01	0	-0.4	0.67
d_{xy}	2.72	0	0	0.67	-1.7

ε_b = 1%

$J_1(1)$, meV	d_{z^2}	d_{xz}	d_{yz}	$d_{x^2-y^2}$	d_{xy}
d_{z^2}	-0.01	0	0	-0.13	2.72
d_{xz}	0	-0.13	-0.22	0	0
d_{yz}	0	-0.42	-0.13	-0.01	0
$d_{x^2-y^2}$	-0.13	-0.01	0	-0.41	0.68
d_{xy}	2.72	0	0	0.68	-1.68

ε_b = 2%

$J_1(1)$, meV	d_{z^2}	d_{xz}	d_{yz}	$d_{x^2-y^2}$	d_{xy}
d_{z^2}	-0.01	0	0	-0.13	2.73
d_{xz}	0	-0.13	-0.24	0	0
d_{yz}	0	-0.41	-0.13	-0.01	0
$d_{x^2-y^2}$	-0.13	-0.01	0	-0.41	0.69
d_{xy}	2.73	0	0	0.69	-1.66

ε_b = 3%

$J_1(1)$, meV	d_{z^2}	d_{xz}	d_{yz}	$d_{x^2-y^2}$	d_{xy}
d_{z^2}	-0.02	0	0	-0.13	2.73
d_{xz}	0	-0.14	-0.25	0	0
d_{yz}	0	-0.39	-0.14	-0.01	0
$d_{x^2-y^2}$	-0.13	-0.01	0	-0.41	0.7
d_{xy}	2.73	0	0	0.7	-1.64

ε_b = 4%

$J_1(1)$, meV	d_{z^2}	d_{xz}	d_{yz}	$d_{x^2-y^2}$	d_{xy}
d_{z^2}	-0.03	0	0	-0.13	2.72
d_{xz}	0	-0.14	-0.26	0	0
d_{yz}	0	-0.37	-0.14	-0.01	0
$d_{x^2-y^2}$	-0.13	-0.01	0	-0.42	0.71
d_{xy}	2.72	0	0	0.71	-1.63

ε_b = 5%

$J_1(1)$, meV	d_{z^2}	d_{xz}	d_{yz}	$d_{x^2-y^2}$	d_{xy}
d_{z^2}	-0.04	0	0	-0.13	2.72
d_{xz}	0	-0.14	-0.26	0	0
d_{yz}	0	-0.36	-0.14	-0.01	0
$d_{x^2-y^2}$	-0.13	-0.01	0	-0.42	0.72
d_{xy}	2.72	0	0	0.72	-1.61

Color code:
t_{2g}-t_{2g}: blue
t_{2g}-e_g: orange
e_g-e_g: green

Figure S32. Orbital-resolved isotropic exchange parameter $J_1(1)$ with applied uniaxial strain along *b*.

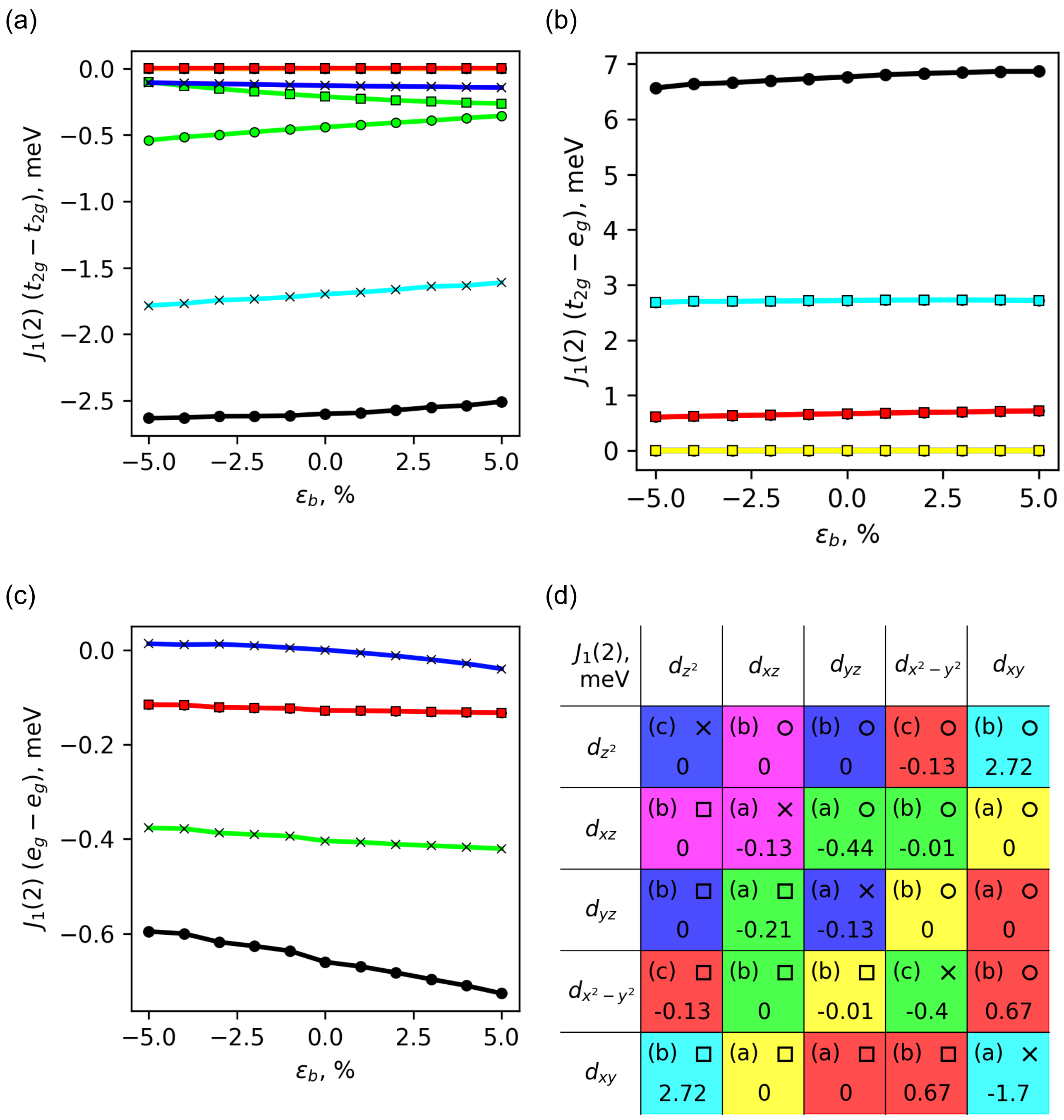

$J_1(2)$, meV	d_{z^2}	d_{xz}	d_{yz}	$d_{x^2-y^2}$	d_{xy}
d_{z^2}	(c) × 0	(b) ○ 0	(b) ○ 0	(c) ○ -0.13	(b) ○ 2.72
d_{xz}	(b) □ 0	(a) × -0.13	(a) ○ -0.44	(b) ○ -0.01	(a) ○ 0
d_{yz}	(b) □ 0	(a) □ -0.21	(a) × -0.13	(b) ○ 0	(a) ○ 0
$d_{x^2-y^2}$	(c) □ -0.13	(b) □ 0	(b) □ -0.01	(c) × -0.4	(b) ○ 0.67
d_{xy}	(b) □ 2.72	(a) □ 0	(a) □ 0	(b) □ 0.67	(a) × -1.7

Figure S33. Orbital-resolved isotropic exchange parameter $J_1(2)$ with applied uniaxial strain along *b*. (a) t_{2g}-t_{2g}; (b) t_{2g}-e_g; (c) e_g-e_g; (d) Color code for the graphs and values for $\varepsilon_b = 0\%$ (d). Black color corresponds to the total exchange of each mechanism (t_{2g}-t_{2g}, t_{2g}-e_g or e_g-e_g).

ε_b = -5%

$J_1(2)$, meV	d_{z^2}	d_{xz}	d_{yz}	$d_{x^2-y^2}$	d_{xy}
d_{z^2}	0.01	0	0	-0.12	2.68
d_{xz}	0	-0.1	-0.54	-0.01	0
d_{yz}	0	-0.1	-0.1	0	0
$d_{x^2-y^2}$	-0.12	0	-0.01	-0.38	0.6
d_{xy}	2.68	0	0	0.6	-1.78

ε_b = -4%

$J_1(2)$, meV	d_{z^2}	d_{xz}	d_{yz}	$d_{x^2-y^2}$	d_{xy}
d_{z^2}	0.01	0	0	-0.12	2.7
d_{xz}	0	-0.11	-0.51	-0.01	0
d_{yz}	0	-0.13	-0.11	0	0
$d_{x^2-y^2}$	-0.12	0	-0.01	-0.38	0.62
d_{xy}	2.7	0	0	0.62	-1.77

ε_b = -3%

$J_1(2)$, meV	d_{z^2}	d_{xz}	d_{yz}	$d_{x^2-y^2}$	d_{xy}
d_{z^2}	0.01	0	0	-0.12	2.7
d_{xz}	0	-0.11	-0.5	-0.01	0
d_{yz}	0	-0.15	-0.11	0	0
$d_{x^2-y^2}$	-0.12	0	-0.01	-0.39	0.63
d_{xy}	2.7	0	0	0.63	-1.74

ε_b = -2%

$J_1(2)$, meV	d_{z^2}	d_{xz}	d_{yz}	$d_{x^2-y^2}$	d_{xy}
d_{z^2}	0.01	0	0	-0.12	2.71
d_{xz}	0	-0.12	-0.48	-0.01	0
d_{yz}	0	-0.17	-0.12	0	0
$d_{x^2-y^2}$	-0.12	0	-0.01	-0.39	0.64
d_{xy}	2.71	0	0	0.64	-1.74

ε_b = -1%

$J_1(2)$, meV	d_{z^2}	d_{xz}	d_{yz}	$d_{x^2-y^2}$	d_{xy}
d_{z^2}	0	0	0	-0.12	2.71
d_{xz}	0	-0.12	-0.46	-0.01	0
d_{yz}	0	-0.19	-0.12	0	0
$d_{x^2-y^2}$	-0.12	0	-0.01	-0.39	0.66
d_{xy}	2.71	0	0	0.66	-1.72

ε_b = 0%

$J_1(2)$, meV	d_{z^2}	d_{xz}	d_{yz}	$d_{x^2-y^2}$	d_{xy}
d_{z^2}	0	0	0	-0.13	2.72
d_{xz}	0	-0.13	-0.44	-0.01	0
d_{yz}	0	-0.21	-0.13	0	0
$d_{x^2-y^2}$	-0.13	0	-0.01	-0.4	0.67
d_{xy}	2.72	0	0	0.67	-1.7

ε_b = 1%

$J_1(2)$, meV	d_{z^2}	d_{xz}	d_{yz}	$d_{x^2-y^2}$	d_{xy}
d_{z^2}	-0.01	0	0	-0.13	2.72
d_{xz}	0	-0.13	-0.42	-0.01	0
d_{yz}	0	-0.22	-0.13	0	0
$d_{x^2-y^2}$	-0.13	0	-0.01	-0.41	0.68
d_{xy}	2.72	0	0	0.68	-1.68

ε_b = 2%

$J_1(2)$, meV	d_{z^2}	d_{xz}	d_{yz}	$d_{x^2-y^2}$	d_{xy}
d_{z^2}	-0.01	0	0	-0.13	2.73
d_{xz}	0	-0.13	-0.41	-0.01	0
d_{yz}	0	-0.24	-0.13	0	0
$d_{x^2-y^2}$	-0.13	0	-0.01	-0.41	0.69
d_{xy}	2.73	0	0	0.69	-1.66

ε_b = 3%

$J_1(2)$, meV	d_{z^2}	d_{xz}	d_{yz}	$d_{x^2-y^2}$	d_{xy}
d_{z^2}	-0.02	0	0	-0.13	2.73
d_{xz}	0	-0.14	-0.39	-0.01	0
d_{yz}	0	-0.25	-0.14	0	0
$d_{x^2-y^2}$	-0.13	0	-0.01	-0.41	0.7
d_{xy}	2.73	0	0	0.7	-1.64

ε_b = 4%

$J_1(2)$, meV	d_{z^2}	d_{xz}	d_{yz}	$d_{x^2-y^2}$	d_{xy}
d_{z^2}	-0.03	0	0	-0.13	2.72
d_{xz}	0	-0.14	-0.37	-0.01	0
d_{yz}	0	-0.26	-0.14	0	0
$d_{x^2-y^2}$	-0.13	0	-0.01	-0.42	0.71
d_{xy}	2.72	0	0	0.71	-1.63

ε_b = 5%

$J_1(2)$, meV	d_{z^2}	d_{xz}	d_{yz}	$d_{x^2-y^2}$	d_{xy}
d_{z^2}	-0.04	0	0	-0.13	2.72
d_{xz}	0	-0.14	-0.36	-0.01	0
d_{yz}	0	-0.26	-0.14	0	0
$d_{x^2-y^2}$	-0.13	0	-0.01	-0.42	0.72
d_{xy}	2.72	0	0	0.72	-1.61

Color code:
t_{2g}-t_{2g}: blue
t_{2g}-e_g: orange
e_g-e_g: green

Figure S34. Orbital-resolved isotropic exchange parameter $J_1(2)$ with applied uniaxial strain along *b*.

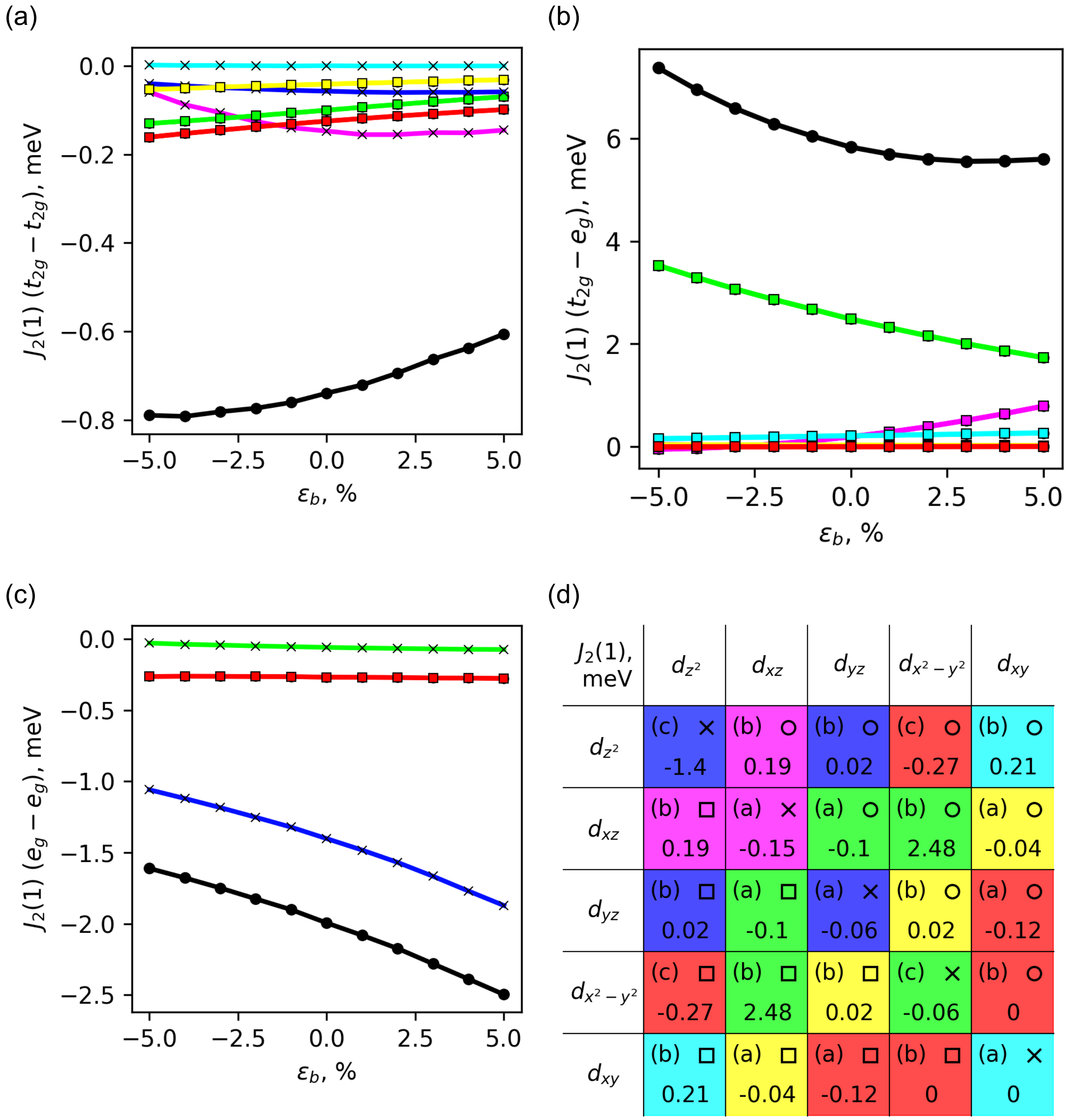

Figure S35. Orbital-resolved isotropic exchange parameter $J_2(1)$ with applied uniaxial strain along *b*. (a) t_{2g}-t_{2g}; (b) t_{2g}-e_g; (c) e_g-e_g; (d) Color code for the graphs and values for ε_b = 0%. Black color corresponds to the total exchange of each mechanism (t_{2g}-t_{2g}, t_{2g}-e_g or e_g-e_g).

ε_b = -5%

$J_2(1)$, meV	d_{z^2}	d_{xz}	d_{yz}	$d_{x^2-y^2}$	d_{xy}
d_{z^2}	-1.06	-0.05	0.04	-0.26	0.15
d_{xz}	-0.05	-0.06	-0.13	3.53	-0.05
d_{yz}	0.04	-0.13	-0.04	0.03	-0.16
$d_{x^2-y^2}$	-0.26	3.53	0.03	-0.03	-0.01
d_{xy}	0.15	-0.05	-0.16	-0.01	0

ε_b = -4%

$J_2(1)$, meV	d_{z^2}	d_{xz}	d_{yz}	$d_{x^2-y^2}$	d_{xy}
d_{z^2}	-1.12	-0.04	0.03	-0.26	0.16
d_{xz}	-0.04	-0.09	-0.12	3.29	-0.05
d_{yz}	0.03	-0.12	-0.05	0.03	-0.15
$d_{x^2-y^2}$	-0.26	3.29	0.03	-0.04	-0.01
d_{xy}	0.16	-0.05	-0.15	-0.01	0

ε_b = -3%

$J_2(1)$, meV	d_{z^2}	d_{xz}	d_{yz}	$d_{x^2-y^2}$	d_{xy}
d_{z^2}	-1.18	0	0.03	-0.26	0.17
d_{xz}	0	-0.11	-0.12	3.07	-0.05
d_{yz}	0.03	-0.12	-0.05	0.03	-0.15
$d_{x^2-y^2}$	-0.26	3.07	0.03	-0.04	-0.01
d_{xy}	0.17	-0.05	-0.15	-0.01	0

ε_b = -2%

$J_2(1)$, meV	d_{z^2}	d_{xz}	d_{yz}	$d_{x^2-y^2}$	d_{xy}
d_{z^2}	-1.25	0.05	0.02	-0.26	0.19
d_{xz}	0.05	-0.13	-0.11	2.87	-0.05
d_{yz}	0.02	-0.11	-0.05	0.03	-0.14
$d_{x^2-y^2}$	-0.26	2.87	0.03	-0.05	-0.01
d_{xy}	0.19	-0.05	-0.14	-0.01	0

ε_b = -1%

$J_2(1)$, meV	d_{z^2}	d_{xz}	d_{yz}	$d_{x^2-y^2}$	d_{xy}
d_{z^2}	-1.32	0.11	0.02	-0.26	0.2
d_{xz}	0.11	-0.14	-0.11	2.68	-0.04
d_{yz}	0.02	-0.11	-0.06	0.03	-0.13
$d_{x^2-y^2}$	-0.26	2.68	0.03	-0.05	0
d_{xy}	0.2	-0.04	-0.13	0	0

ε_b = 0%

$J_2(1)$, meV	d_{z^2}	d_{xz}	d_{yz}	$d_{x^2-y^2}$	d_{xy}
d_{z^2}	-1.4	0.19	0.02	-0.27	0.21
d_{xz}	0.19	-0.15	-0.1	2.48	-0.04
d_{yz}	0.02	-0.1	-0.06	0.02	-0.12
$d_{x^2-y^2}$	-0.27	2.48	0.02	-0.06	0
d_{xy}	0.21	-0.04	-0.12	0	0

ε_b = 1%

$J_2(1)$, meV	d_{z^2}	d_{xz}	d_{yz}	$d_{x^2-y^2}$	d_{xy}
d_{z^2}	-1.48	0.28	0.01	-0.27	0.22
d_{xz}	0.28	-0.16	-0.09	2.32	-0.04
d_{yz}	0.01	-0.09	-0.06	0.02	-0.12
$d_{x^2-y^2}$	-0.27	2.32	0.02	-0.06	0
d_{xy}	0.22	-0.04	-0.12	0	0

ε_b = 2%

$J_2(1)$, meV	d_{z^2}	d_{xz}	d_{yz}	$d_{x^2-y^2}$	d_{xy}
d_{z^2}	-1.57	0.39	0.01	-0.27	0.23
d_{xz}	0.39	-0.16	-0.09	2.16	-0.04
d_{yz}	0.01	-0.09	-0.06	0.02	-0.11
$d_{x^2-y^2}$	-0.27	2.16	0.02	-0.07	0
d_{xy}	0.23	-0.04	-0.11	0	0

ε_b = 3%

$J_2(1)$, meV	d_{z^2}	d_{xz}	d_{yz}	$d_{x^2-y^2}$	d_{xy}
d_{z^2}	-1.67	0.51	0.01	-0.27	0.24
d_{xz}	0.51	-0.15	-0.08	2.0	-0.04
d_{yz}	0.01	-0.08	-0.06	0.02	-0.11
$d_{x^2-y^2}$	-0.27	2.0	0.02	-0.07	0
d_{xy}	0.24	-0.04	-0.11	0	0

ε_b = 4%

$J_2(1)$, meV	d_{z^2}	d_{xz}	d_{yz}	$d_{x^2-y^2}$	d_{xy}
d_{z^2}	-1.77	0.64	0	-0.27	0.25
d_{xz}	0.64	-0.15	-0.08	1.87	-0.03
d_{yz}	0	-0.08	-0.06	0.02	-0.1
$d_{x^2-y^2}$	-0.27	1.87	0.02	-0.07	0
d_{xy}	0.25	-0.03	-0.1	0	0

ε_b = 5%

$J_2(1)$, meV	d_{z^2}	d_{xz}	d_{yz}	$d_{x^2-y^2}$	d_{xy}
d_{z^2}	-1.87	0.79	0	-0.28	0.26
d_{xz}	0.79	-0.15	-0.07	1.73	-0.03
d_{yz}	0	-0.07	-0.06	0.02	-0.1
$d_{x^2-y^2}$	-0.28	1.73	0.02	-0.07	0
d_{xy}	0.26	-0.03	-0.1	0	0

Color code:
t_{2g}-t_{2g}: blue
t_{2g}-e_g: orange
e_g-e_g: green

Figure S36. Orbital-resolved isotropic exchange parameter $J_2(1)$ with applied uniaxial strain along *b*.

(a)

(b)

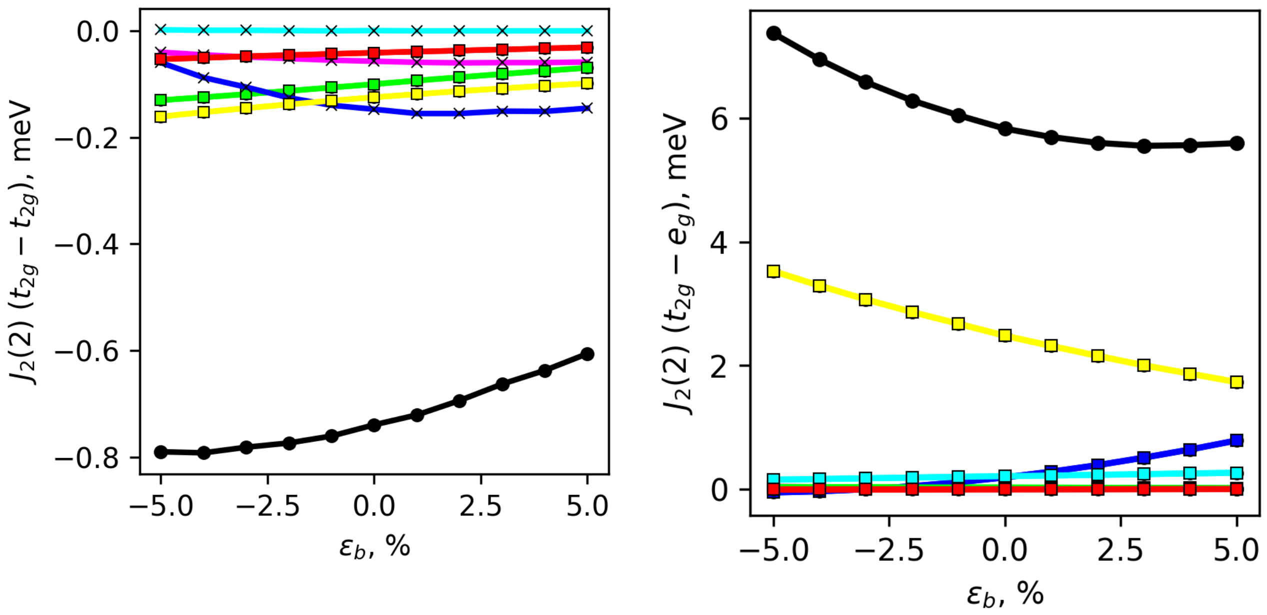

(c)

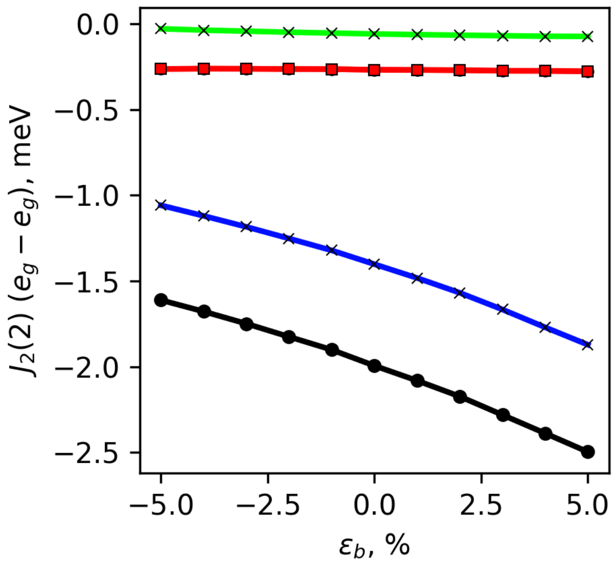

(d)

$J_2(2)$, meV	d_{z^2}	d_{xz}	d_{yz}	$d_{x^2-y^2}$	d_{xy}
d_{z^2}	(c) × -1.4	(b) ○ 0.02	(b) ○ 0.19	(c) ○ -0.27	(b) ○ 0.21
d_{xz}	(b) □ 0.02	(a) × -0.06	(a) ○ -0.1	(b) ○ 0.02	(a) ○ -0.12
d_{yz}	(b) □ 0.19	(a) □ -0.1	(a) × -0.15	(b) ○ 2.48	(a) ○ -0.04
$d_{x^2-y^2}$	(c) □ -0.27	(b) □ 0.02	(b) □ 2.48	(c) × -0.06	(b) ○ 0
d_{xy}	(b) □ 0.21	(a) □ -0.12	(a) □ -0.04	(b) □ 0	(a) × 0

Figure S37. Orbital-resolved isotropic exchange parameter $J_2(2)$ with applied uniaxial strain along *b*. (a) t_{2g}-t_{2g}; (b) t_{2g}-e_g; (c) e_g-e_g; (d) Color code for the graphs and values for $\varepsilon_b = 0\%$ (d). Black color corresponds to the total exchange of each mechanism (t_{2g}-t_{2g}, t_{2g}-e_g or e_g-e_g).

ε_b = -5%

$J_2(2)$, meV	d_{z^2}	d_{xz}	d_{yz}	$d_{x^2-y^2}$	d_{xy}
d_{z^2}	-1.06	0.04	-0.05	-0.26	0.15
d_{xz}	0.04	-0.04	-0.13	0.03	-0.16
d_{yz}	-0.05	-0.13	-0.06	3.53	-0.05
$d_{x^2-y^2}$	-0.26	0.03	3.53	-0.03	-0.01
d_{xy}	0.15	-0.16	-0.05	-0.01	0

ε_b = -4%

$J_2(2)$, meV	d_{z^2}	d_{xz}	d_{yz}	$d_{x^2-y^2}$	d_{xy}
d_{z^2}	-1.12	0.03	-0.04	-0.26	0.16
d_{xz}	0.03	-0.05	-0.12	0.03	-0.15
d_{yz}	-0.04	-0.12	-0.09	3.29	-0.05
$d_{x^2-y^2}$	-0.26	0.03	3.29	-0.04	-0.01
d_{xy}	0.16	-0.15	-0.05	-0.01	0

ε_b = -3%

$J_2(2)$, meV	d_{z^2}	d_{xz}	d_{yz}	$d_{x^2-y^2}$	d_{xy}
d_{z^2}	-1.18	0.03	0	-0.26	0.17
d_{xz}	0.03	-0.05	-0.12	0.03	-0.15
d_{yz}	0	-0.12	-0.11	3.07	-0.05
$d_{x^2-y^2}$	-0.26	0.03	3.07	-0.04	-0.01
d_{xy}	0.17	-0.15	-0.05	-0.01	0

ε_b = -2%

$J_2(2)$, meV	d_{z^2}	d_{xz}	d_{yz}	$d_{x^2-y^2}$	d_{xy}
d_{z^2}	-1.25	0.02	0.05	-0.26	0.19
d_{xz}	0.02	-0.05	-0.11	0.03	-0.14
d_{yz}	0.05	-0.11	-0.13	2.87	-0.05
$d_{x^2-y^2}$	-0.26	0.03	2.87	-0.05	-0.01
d_{xy}	0.19	-0.14	-0.05	-0.01	0

ε_b = -1%

$J_2(2)$, meV	d_{z^2}	d_{xz}	d_{yz}	$d_{x^2-y^2}$	d_{xy}
d_{z^2}	-1.32	0.02	0.11	-0.26	0.2
d_{xz}	0.02	-0.06	-0.11	0.03	-0.13
d_{yz}	0.11	-0.11	-0.14	2.68	-0.04
$d_{x^2-y^2}$	-0.26	0.03	2.68	-0.05	0
d_{xy}	0.2	-0.13	-0.04	0	0

ε_b = 0%

$J_2(2)$, meV	d_{z^2}	d_{xz}	d_{yz}	$d_{x^2-y^2}$	d_{xy}
d_{z^2}	-1.4	0.02	0.19	-0.27	0.21
d_{xz}	0.02	-0.06	-0.1	0.02	-0.12
d_{yz}	0.19	-0.1	-0.15	2.48	-0.04
$d_{x^2-y^2}$	-0.27	0.02	2.48	-0.06	0
d_{xy}	0.21	-0.12	-0.04	0	0

ε_b = 1%

$J_2(2)$, meV	d_{z^2}	d_{xz}	d_{yz}	$d_{x^2-y^2}$	d_{xy}
d_{z^2}	-1.48	0.01	0.28	-0.27	0.22
d_{xz}	0.01	-0.06	-0.09	0.02	-0.12
d_{yz}	0.28	-0.09	-0.16	2.32	-0.04
$d_{x^2-y^2}$	-0.27	0.02	2.32	-0.06	0
d_{xy}	0.22	-0.12	-0.04	0	0

ε_b = 2%

$J_2(2)$, meV	d_{z^2}	d_{xz}	d_{yz}	$d_{x^2-y^2}$	d_{xy}
d_{z^2}	-1.57	0.01	0.39	-0.27	0.23
d_{xz}	0.01	-0.06	-0.09	0.02	-0.11
d_{yz}	0.39	-0.09	-0.16	2.16	-0.04
$d_{x^2-y^2}$	-0.27	0.02	2.16	-0.07	0
d_{xy}	0.23	-0.11	-0.04	0	0

ε_b = 3%

$J_2(2)$, meV	d_{z^2}	d_{xz}	d_{yz}	$d_{x^2-y^2}$	d_{xy}
d_{z^2}	-1.67	0.01	0.51	-0.27	0.24
d_{xz}	0.01	-0.06	-0.08	0.02	-0.11
d_{yz}	0.51	-0.08	-0.15	2.0	-0.04
$d_{x^2-y^2}$	-0.27	0.02	2.0	-0.07	0
d_{xy}	0.24	-0.11	-0.04	0	0

ε_b = 4%

$J_2(2)$, meV	d_{z^2}	d_{xz}	d_{yz}	$d_{x^2-y^2}$	d_{xy}
d_{z^2}	-1.77	0	0.64	-0.27	0.25
d_{xz}	0	-0.06	-0.08	0.02	-0.1
d_{yz}	0.64	-0.08	-0.15	1.87	-0.03
$d_{x^2-y^2}$	-0.27	0.02	1.87	-0.07	0
d_{xy}	0.25	-0.1	-0.03	0	0

ε_b = 5%

$J_2(2)$, meV	d_{z^2}	d_{xz}	d_{yz}	$d_{x^2-y^2}$	d_{xy}
d_{z^2}	-1.87	0	0.79	-0.28	0.26
d_{xz}	0	-0.06	-0.07	0.02	-0.1
d_{yz}	0.79	-0.07	-0.15	1.73	-0.03
$d_{x^2-y^2}$	-0.28	0.02	1.73	-0.07	0
d_{xy}	0.26	-0.1	-0.03	0	0

Color code:
t_{2g}-t_{2g}: blue
t_{2g}-e_g: orange
e_g-e_g: green

Figure S38. Orbital-resolved isotropic exchange parameter $J_2(2)$ with applied uniaxial strain along *b*.

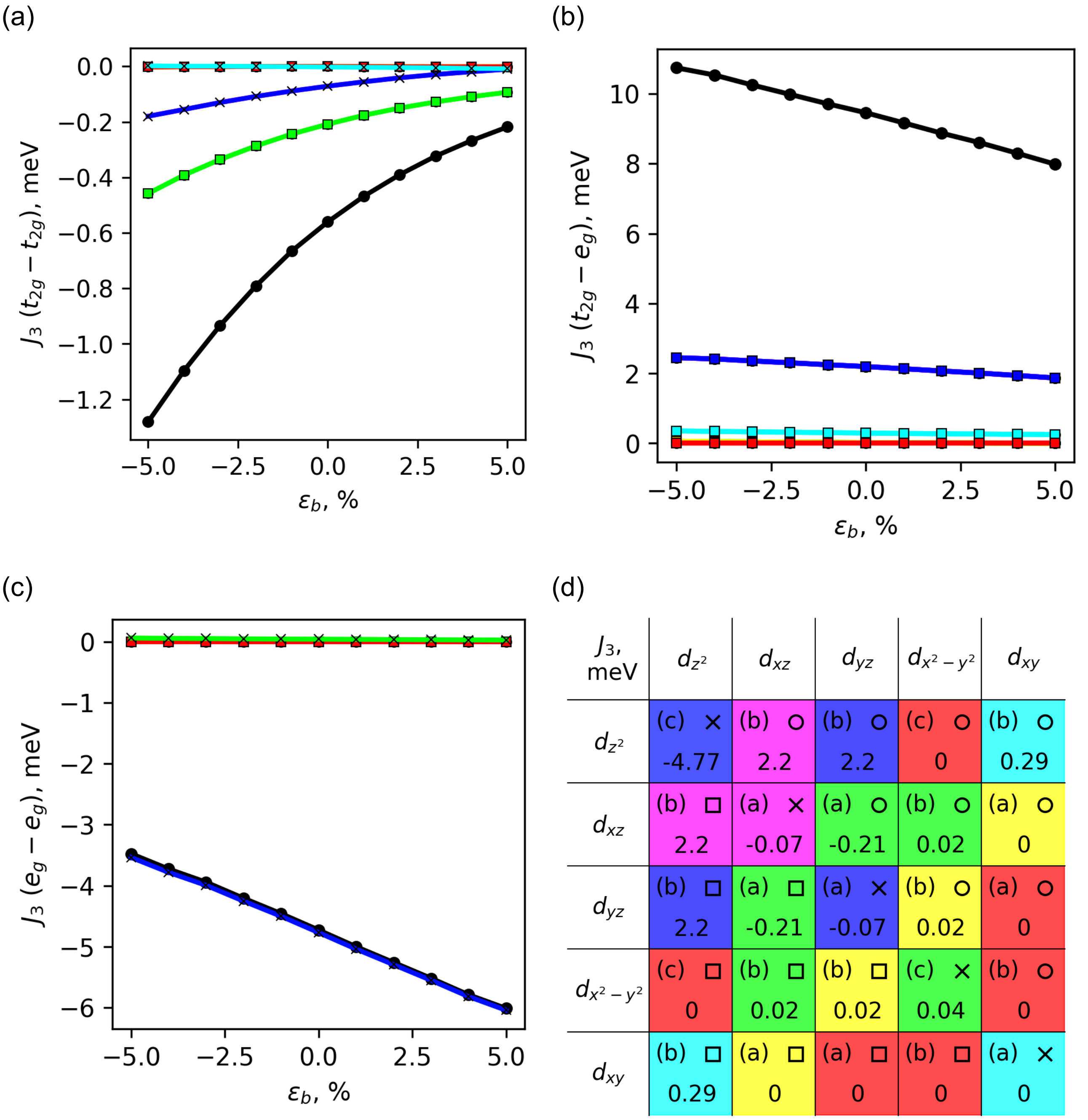

J_3, meV	d_{z^2}	d_{xz}	d_{yz}	$d_{x^2-y^2}$	d_{xy}
d_{z^2}	(c) × -4.77	(b) ○ 2.2	(b) ○ 2.2	(c) ○ 0	(b) ○ 0.29
d_{xz}	(b) □ 2.2	(a) × -0.07	(a) ○ -0.21	(b) ○ 0.02	(a) ○ 0
d_{yz}	(b) □ 2.2	(a) □ -0.21	(a) × -0.07	(b) ○ 0.02	(a) ○ 0
$d_{x^2-y^2}$	(c) □ 0	(b) □ 0.02	(b) □ 0.02	(c) × 0.04	(b) ○ 0
d_{xy}	(b) □ 0.29	(a) □ 0	(a) □ 0	(b) □ 0	(a) × 0

Figure S39. Orbital-resolved isotropic exchange parameter J_3 with applied uniaxial strain along *b*. (a) t_{2g}-t_{2g}; (b) t_{2g}-e_g; (c) e_g-e_g; (d) Color code for the graphs and values for ε_b = 0% (d). Black color corresponds to the total exchange of each mechanism (t_{2g}-t_{2g}, t_{2g}-e_g or e_g-e_g).

ε_b = -5%

J_3, meV	d_{z^2}	d_{xz}	d_{yz}	$d_{x^2-y^2}$	d_{xy}
d_{z^2}	-3.54	2.45	2.45	0	0.35
d_{xz}	2.45	-0.18	-0.46	0.06	0
d_{yz}	2.45	-0.46	-0.18	0.06	0
$d_{x^2-y^2}$	0	0.06	0.06	0.06	0
d_{xy}	0.35	0	0	0	0

ε_b = -4%

J_3, meV	d_{z^2}	d_{xz}	d_{yz}	$d_{x^2-y^2}$	d_{xy}
d_{z^2}	-3.78	2.41	2.41	0	0.34
d_{xz}	2.41	-0.16	-0.39	0.05	0
d_{yz}	2.41	-0.39	-0.16	0.05	0
$d_{x^2-y^2}$	0	0.05	0.05	0.05	0
d_{xy}	0.34	0	0	0	0

ε_b = -3%

J_3, meV	d_{z^2}	d_{xz}	d_{yz}	$d_{x^2-y^2}$	d_{xy}
d_{z^2}	-3.99	2.36	2.36	0	0.33
d_{xz}	2.36	-0.13	-0.34	0.04	0
d_{yz}	2.36	-0.34	-0.13	0.04	0
$d_{x^2-y^2}$	0	0.04	0.04	0.05	0
d_{xy}	0.33	0	0	0	0

ε_b = -2%

J_3, meV	d_{z^2}	d_{xz}	d_{yz}	$d_{x^2-y^2}$	d_{xy}
d_{z^2}	-4.25	2.3	2.3	0	0.32
d_{xz}	2.3	-0.11	-0.29	0.03	0
d_{yz}	2.3	-0.29	-0.11	0.03	0
$d_{x^2-y^2}$	0	0.03	0.03	0.05	0
d_{xy}	0.32	0	0	0	0

ε_b = -1%

J_3, meV	d_{z^2}	d_{xz}	d_{yz}	$d_{x^2-y^2}$	d_{xy}
d_{z^2}	-4.5	2.25	2.25	0	0.3
d_{xz}	2.25	-0.09	-0.24	0.03	0
d_{yz}	2.25	-0.24	-0.09	0.03	0
$d_{x^2-y^2}$	0	0.03	0.03	0.04	0
d_{xy}	0.3	0	0	0	0

ε_b = 0%

J_3, meV	d_{z^2}	d_{xz}	d_{yz}	$d_{x^2-y^2}$	d_{xy}
d_{z^2}	-4.77	2.2	2.2	0	0.29
d_{xz}	2.2	-0.07	-0.21	0.02	0
d_{yz}	2.2	-0.21	-0.07	0.02	0
$d_{x^2-y^2}$	0	0.02	0.02	0.04	0
d_{xy}	0.29	0	0	0	0

ε_b = 1%

J_3, meV	d_{z^2}	d_{xz}	d_{yz}	$d_{x^2-y^2}$	d_{xy}
d_{z^2}	-5.04	2.13	2.13	0	0.28
d_{xz}	2.13	-0.06	-0.18	0.02	0
d_{yz}	2.13	-0.18	-0.06	0.02	0
$d_{x^2-y^2}$	0	0.02	0.02	0.04	0
d_{xy}	0.28	0	0	0	0

ε_b = 2%

J_3, meV	d_{z^2}	d_{xz}	d_{yz}	$d_{x^2-y^2}$	d_{xy}
d_{z^2}	-5.29	2.07	2.07	0	0.27
d_{xz}	2.07	-0.04	-0.15	0.01	0
d_{yz}	2.07	-0.15	-0.04	0.01	0
$d_{x^2-y^2}$	0	0.01	0.01	0.04	0
d_{xy}	0.27	0	0	0	0

ε_b = 3%

J_3, meV	d_{z^2}	d_{xz}	d_{yz}	$d_{x^2-y^2}$	d_{xy}
d_{z^2}	-5.55	2.01	2.01	0	0.27
d_{xz}	2.01	-0.03	-0.13	0.01	0
d_{yz}	2.01	-0.13	-0.03	0.01	0
$d_{x^2-y^2}$	0	0.01	0.01	0.03	0
d_{xy}	0.27	0	0	0	0

ε_b = 4%

J_3, meV	d_{z^2}	d_{xz}	d_{yz}	$d_{x^2-y^2}$	d_{xy}
d_{z^2}	-5.82	1.94	1.94	0	0.26
d_{xz}	1.94	-0.02	-0.11	0	0
d_{yz}	1.94	-0.11	-0.02	0	0
$d_{x^2-y^2}$	0	0	0	0.03	0
d_{xy}	0.26	0	0	0	-0.01

ε_b = 5%

J_3, meV	d_{z^2}	d_{xz}	d_{yz}	$d_{x^2-y^2}$	d_{xy}
d_{z^2}	-6.04	1.87	1.87	0	0.25
d_{xz}	1.87	-0.01	-0.09	0	0
d_{yz}	1.87	-0.09	-0.01	0	0
$d_{x^2-y^2}$	0	0	0	0.03	0
d_{xy}	0.25	0	0	0	-0.01

Color code:
t_{2g}-t_{2g}: blue
t_{2g}-e_g: orange
e_g-e_g: green

Figure S40. Orbital-resolved isotropic exchange parameter J_3 with applied uniaxial strain along *b*.

3.6 Magnetic moment per atom vs strain applied along *b* (U = 3 eV)

(a)

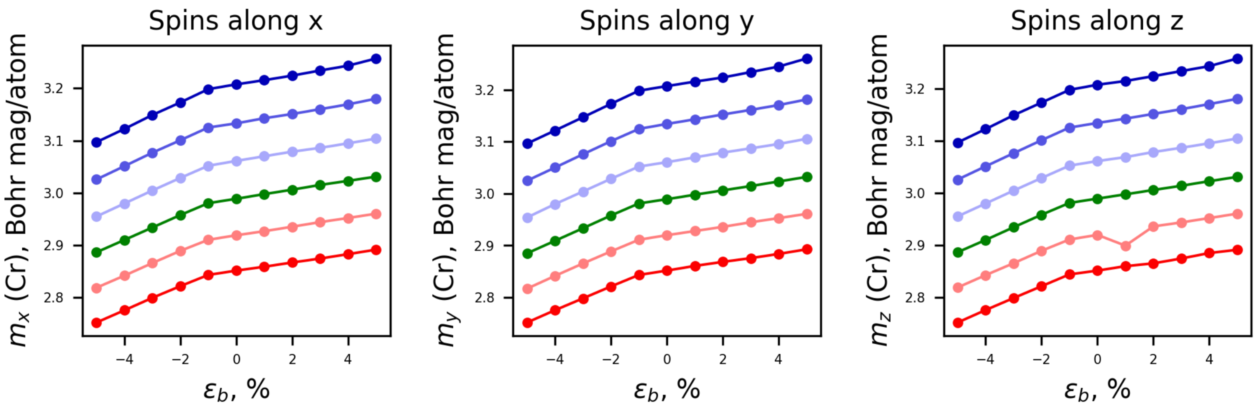

(b)

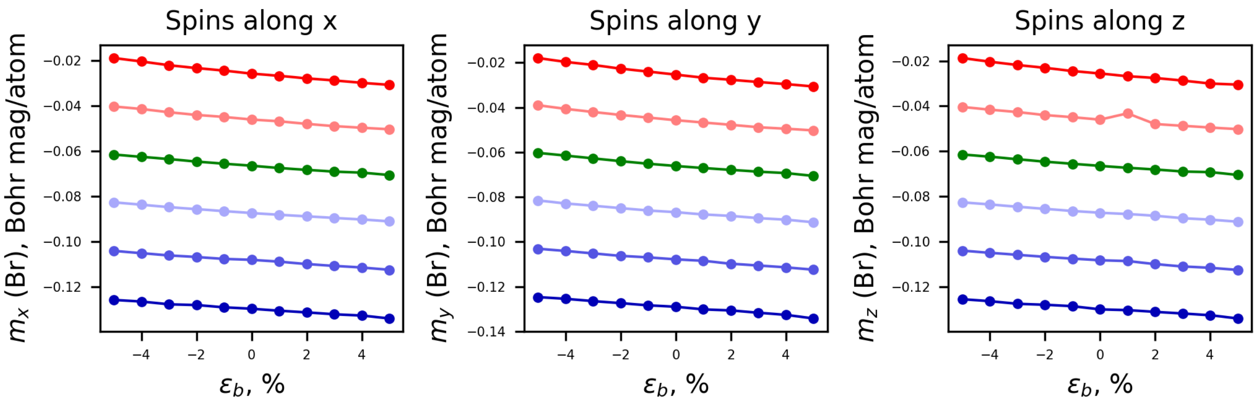

(c)

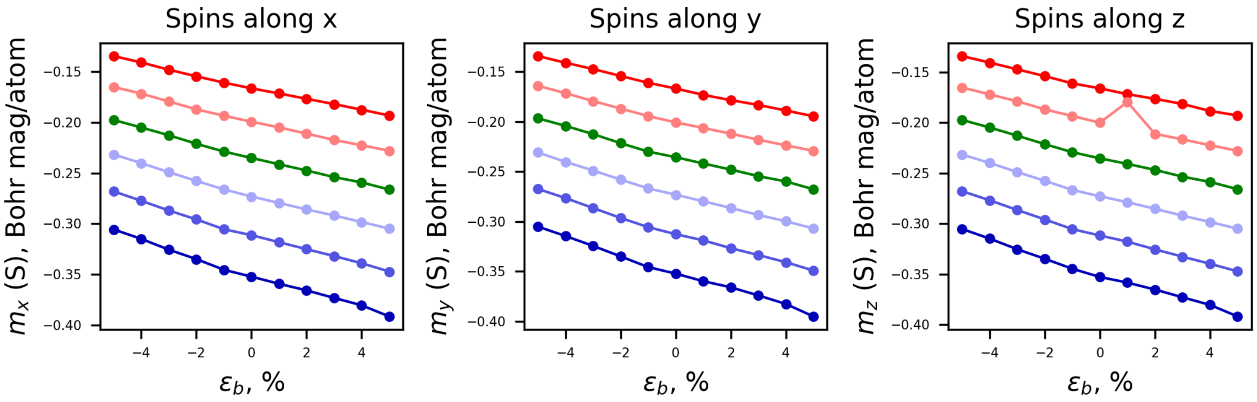

Figure S41. Magnetic moment per (a) Cr, (b) Br (c) S atom for strain applied along *b* for each type of strain three DFT calculations are presented with alignment of spins along x, y or z Cartesian axis (specified in the pictures). Colors from red to blue corresponds to the values of Hubbard U - from 1 eV to 6 eV (with the step of 1 eV, i.e., green corresponds to 3 eV)

4. Hopping integrals

In this section we present the hopping parameters between the atoms present in the unit cell, extracted from the Wannier Hamiltonian. The section starts with a discussion to summarize the main results, Fig. S42 present a view of CrSBr structure where the atoms are labeled according to the criteria used in the Tables S6-S20 which present the hopping parameters.

The analysis of hopping parameters agrees with the orbital resolved exchange picture. The interaction between first neighbors is mainly originated by the competition between d_{xy}-d_{z^2} (t_{2g}-e_g) FM channel and d_{xy}-d_{xy} (t_{2g}-t_{2g}) AFM pathway, where the p_x and p_y orbitals of both S and Br atoms mediate the super exchange interaction. The second neighbors can be explained in terms of two pathways namely the FM t_{2g}-e_g and the AFM e_g-e_g. The FM is governed by the hopping between d_{xz} (d_{yz}) and d_{x2-y2} Cr orbitals. This pathway is mediated by p_x (p_y) coupled with the d_{xz} (d_{yz}) and the p_y (p_x) with the d_{x2-y2}. Between orbitals of Sulfur there is Coulomb exchange. The e_g-e_g interaction is governed by exchange between d_{z^2} of two Cr atoms. This interaction is mediated by p_y and p_x orbitals of Sulphur along *a* which form a sigma bond with the d_{z^2} orbitals. Thus, the p_z orbital is overlapping with the d_{z^2} of Cr and forming another sigma bond. In the third neighbor t_{2g}-e_g (FM) is governed by hopping between d_{xz}/d_{yz} and d_{z^2} Cr orbitals through p_x/p_y and p_z orbitals of S. The e_g-e_g interaction (AFM) is governed by hopping between d_{z^2} and d_{z^2} Cr orbitals through p_z orbitals of S. Under application of tensile strain, we observe that the overall hopping parameters decrease as atoms are further away, while there is an increase of the on-site energies.

For J_1 0% of strain, the angles between Cr-S-Cr (α) and Cr-Br-Cr (β) are 96° and 90°, respectively. For the Cr-S-Cr pathway, α changes from 92.5° to 98.5° under -5% to +5% of strain along *a* while β

ranges from 86.5° to 92.5°. According to the Kanamori rules, a FM behavior is expected given that α and β are close to 90°. For the whole range of strain there is an almost constant behavior in the contribution of t_{2g}-e_g orbitals to the FM exchange of the system, however the system turns more FM when *a* is expanded as a result of the weaker overlapping between d_{xy}-p_x-p_y-d_{xy} (t_{2g}-t_{2g}) orbitals of Cr atoms and ligands and thus reducing the AFM character.

J_2 interaction is influenced by the angle between Cr-S-Cr (γ) for 0% of strain is 97°, but we do not observe pronounced differences in γ either for modification in *a* or in *b*, which only varies ± 0.7 in the regimes of ± 5 % of strain. J_2 remains almost constant under strain in *a* as a consequence of the unchanged e_g-e_g, t_{2g}-t_{2g} and t_{2g}-e_g mechanisms. There is only a slightly dependence between t_{2g}-e_g and strain in *b*. In this case, compression in *b* enhances this t_{2g}-e_g mechanism, thus favoring the overall FM character.

The angle between Cr-S-Cr (δ) for 0% of strain is 157.6°. Modifications in *a* are not affecting either δ or J_3 and thus keeping the overall FM character almost constant. However, when a variation along *b* is induced, there is a progressive enhancement of the AFM behavior by ranging of strain along this crystallographic axis from -5 to +5 %. This is due to the better alignment the d_z^2-p_z- d_z^2 orbitals of Cr and S atoms, which enhance AFM channel that governs this pathway.

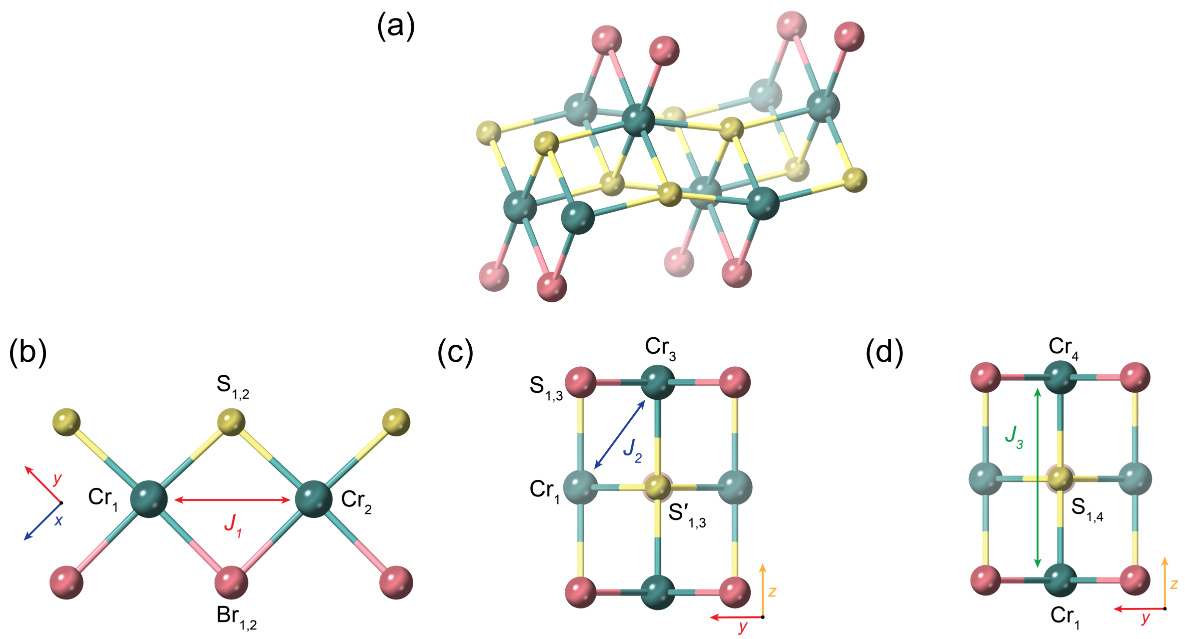

Figure S42. (a) Lateral view of monolayer CrSBr. (b) Schematic representation of the different magnetic exchange channels J_1 (a), J_2 (b) and J_3 (c), where Cr, Br and S atoms are numbered for identifying the hopping terms that govern each channel (Tables S6-S20). Note that in (b) $S_{1,3}$ is referring to a Sulfur that is hidden below a Br atom.

Table S6. Absolute values of the hopping integrals between *d* orbitals of Cr_1 and Cr_2 atoms and *p* orbitals of $S_{1,2}$ and $Br_{1,2}$. Hopping between orbitals involved in the most relevant magnetic superexchange channels for J_1 are highlighted in bold.

		**$S_{1,2}$ (p_z)**	**$S_{1,2}$ (p_x)**	**$S_{1,2}$ (p_y)**	**$Br_{1,2}$ (p_z)**	**$Br_{1,2}$ (p_x)**	**$Br_{1,2}$ (p_y)**
**Cr_1**	**d_{z2}**	0.00	0.71	0.09	0.00	0.03	0.56
	**d_{xz}**	0.55	0.00	0.00	0.02	0.00	0.00
	**d_{yz}**	0.07	0.00	0.00	0.58	0.00	0.00
	**d_{x2-y2}**	0.00	1.14	0.14	0.00	0.01	1.08
	**d_{xy}**	0.00	0.13	**0.63**	0.00	**0.52**	0.01
**Cr_2**	**d_{z2}**	0.00	0.09	**0.71**	0.00	**0.56**	0.03
	**d_{xz}**	0.07	0.00	0.00	0.58	0.00	0.00
	**d_{yz}**	0.55	0.00	0.00	0.02	0.00	0.00
	**d_{x2-y2}**	0.00	0.14	1.14	0.00	1.08	0.01
	**d_{xy}**	0.00	**0.63**	0.13	0.00	0.01	**0.52**

Table S7. Absolute values of the hopping integrals between *d* orbitals of Cr_1 and Cr_2 atoms and *p* orbitals of $S_{1,2}$ and $Br_{1,2}$ for 5% compressive strain along *a*. Hopping between orbitals involved in the most relevant magnetic superexchange channels for J_1 are highlighted in bold.

		**$S_{1,2}$ (p_z)**	**$S_{1,2}$ (p_x)**	**$S_{1,2}$ (p_y)**	**$Br_{1,2}$ (p_z)**	**$Br_{1,2}$ (p_x)**	**$Br_{1,2}$ (p_y)**
**Cr_1**	**d_{z2}**	0.00	0.74	0.07	0.00	0.01	0.58
	**d_{xz}**	0.64	0.00	0.00	0.03	0.00	0.00
	**d_{yz}**	0.05	0.00	0.00	0.65	0.00	0.00
	**d_{x2-y2}**	0.00	1.26	0.12	0.00	0.05	1.19
	**d_{xy}**	0.00	0.09	**0.74**	0.00	**0.60**	0.04
**Cr_2**	**d_{z2}**	0.00	0.07	**0.74**	0.00	**0.58**	0.01
	**d_{xz}**	0.05	0.00	0.00	0.65	0.00	0.00
	**d_{yz}**	0.64	0.00	0.00	0.03	0.00	0.00
	**d_{x2-y2}**	0.00	0.12	1.26	0.00	1.19	0.05
	**d_{xy}**	0.00	**0.74**	0.09	0.00	0.04	**0.60**

Table S8. Absolute values of the hopping integrals between *d* orbitals of Cr_1 and Cr_2 atoms and *p* orbitals of $S_{1,2}$ and $Br_{1,2}$ for 5% tensile strain along *a*. Hopping between orbitals involved in the most relevant magnetic superexchange channels for J_1 are highlighted in bold

		**$S_{1,2}$ (p_z)**	**$S_{1,2}$ (p_x)**	**$S_{1,2}$ (p_y)**	**$Br_{1,2}$ (p_z)**	**$Br_{1,2}$ (p_x)**	**$Br_{1,2}$ (p_y)**
**Cr_1**	**d_{z2}**	0.00	0.69	0.10	0.00	0.04	0.54
	**d_{xz}**	0.48	0.00	0.00	0.01	0.00	0.00
	**d_{yz}**	0.07	0.00	0.00	0.52	0.00	0.00
	**d_{x2-y2}**	0.00	1.04	0.16	0.00	0.02	0.98
	**d_{xy}**	0.00	0.17	**0.54**	0.00	**0.45**	0.05
**Cr_2**	**d_{z2}**	0.00	0.10	**0.69**	0.00	**0.54**	0.04
	**d_{xz}**	0.07	0.00	0.00	0.52	0.00	0.00
	**d_{yz}**	0.48	0.00	0.00	0.01	0.00	0.00
	**d_{x2-y2}**	0.00	0.16	1.04	0.00	0.98	0.02
	**d_{xy}**	0.00	**0.54**	0.17	0.00	0.05	**0.45**

Table S9. Absolute values of the hopping integrals between *d* orbitals of Cr_1 and Cr_2 atoms and *p* orbitals of $S_{1,2}$ and $Br_{1,2}$ for 5% compressive strain along *b*. Hopping between orbitals involved in the most relevant magnetic superexchange channels for J_1 are highlighted in bold.

		$S_{1,2}$ (p_z)	$S_{1,2}$ (p_x)	$S_{1,2}$ (p_y)	$Br_{1,2}$ (p_z)	$Br_{1,2}$ (p_x)	$Br_{1,2}$ (p_y)
Cr_1	d_{z2}	0.00	0.83	0.10	0.00	0.03	0.63
	d_{xz}	0.53	0.00	0.00	0.03	0.00	0.00
	d_{yz}	0.08	0.00	0.00	0.59	0.00	0.00
	d_{x2-y2}	0.00	1.16	0.14	0.00	0.01	1.08
	d_{xy}	0.00	0.14	**0.64**	0.00	**0.53**	0.02
Cr_2	d_{z2}	0.00	0.10	**0.83**	0.00	**0.63**	0.03
	d_{xz}	0.08	0.00	0.00	0.59	0.00	0.00
	d_{yz}	0.53	0.00	0.00	0.03	0.00	0.00
	d_{x2-y2}	0.00	0.14	1.16	0.00	1.08	0.01
	d_{xy}	0.00	**0.64**	0.14	0.00	0.02	**0.53**

Table S10. Absolute values of the hopping integrals between *d* orbitals of Cr_1 and Cr_2 atoms and *p* orbitals of $S_{1,2}$ and $Br_{1,2}$ for 5% tensile strain along *b*. Hopping between orbitals involved in the most relevant magnetic superexchange channels for J_1 are highlighted in bold

		**$S_{1,2}$ (p_z)**	**$S_{1,2}$ (p_x)**	**$S_{1,2}$ (p_y)**	**$Br_{1,2}$ (p_z)**	**$Br_{1,2}$ (p_x)**	**$Br_{1,2}$ (p_y)**
**Cr_1**	**d_{z2}**	0.00	0.61	0.07	0.00	0.03	0.50
	**d_{xz}**	0.57	0.00	0.00	0.01	0.00	0.00
	**d_{yz}**	0.06	0.00	0.00	0.58	0.00	0.00
	**d_{x2-y2}**	0.00	1.13	0.15	0.00	0.01	1.09
	**d_{xy}**	0.00	0.13	**0.63**	0.00	**0.52**	0.00
**Cr_2**	**d_{z2}**	0.00	0.07	**0.61**	0.00	**0.50**	0.03
	**d_{xz}**	0.06	0.00	0.00	0.58	0.00	0.00
	**d_{yz}**	0.57	0.00	0.00	0.01	0.00	0.00
	**d_{x2-y2}**	0.00	0.15	1.13	0.00	1.09	0.01
	**d_{xy}**	0.00	**0.63**	0.13	0.00	0.00	**0.52**

Table S11. Absolute values of the hopping integrals between *d* orbitals of Cr_1 and Cr_3 atoms and *p* orbitals of $S_{1,3}$ and $S'_{1,3}$. Hopping between orbitals involved in the most relevant magnetic superexchange channels for J_2 are highlighted in bold.

		$S_{1,3}$ (p_z)	$S_{1,3}$ (p_x)	$S_{1,3}$ (p_y)	$S'_{1,3}$ (p_z)	$S'_{1,3}$ (p_x)	$S'_{1,3}$ (p_y)
Cr_1	d_{z2}	**1.18**	0.31	0.31	0.00	0.09	**0.71**
	d_{xz}	0.33	**0.51**	0.08	0.07	0.00	0.00
	d_{yz}	0.33	0.08	**0.51**	0.55	0.00	0.00
	d_{x2-y2}	0.00	0.00	0.00	0.00	0.14	1.14
	d_{xy}	0.08	0.08	0.08	0.00	0.63	0.14
Cr_3	d_{z2}	0.00	0.09	**0.71**	**1.19**	0.31	0.31
	d_{xz}	0.07	0.00	0.00	0.33	0.51	0.08
	d_{yz}	0.55	0.00	0.00	0.33	0.08	0.51
	d_{x2-y2}	0.00	0.14	**1.14**	0.00	0.00	0.00
	d_{xy}	0.00	0.63	0.14	0.08	0.08	0.08

Table S12. Absolute values of the hopping integrals between *d* orbitals of Cr_1 and Cr_3 atoms and *p* orbitals of $S_{1,3}$ and $S'_{1,3}$ for 5% compressive strain along *a*. Hopping between orbitals involved in the most relevant magnetic superexchange channels for J_2 are highlighted in bold.

		$S_{1,3}$ (p_z)	$S_{1,3}$ (p_x)	$S_{1,3}$ (p_y)	$S'_{1,3}$ (p_z)	$S'_{1,3}$ (p_x)	$S'_{1,3}$ (p_y)
Cr_1	d_{z2}	**1.23**	0.32	0.32	0.00	0.07	**0.74**
	d_{xz}	0.34	**0.51**	0.08	0.05	0.00	0.00
	d_{yz}	0.34	0.08	**0.51**	0.64	0.00	0.00
	d_{x2-y2}	0.00	0.01	0.01	0.00	0.13	1.26
	d_{xy}	0.06	0.08	0.08	0.00	0.74	0.09
Cr_3	d_{z2}	0.00	0.07	**0.74**	**1.23**	0.32	0.32
	d_{xz}	0.05	0.00	0.00	0.34	0.51	0.08
	d_{yz}	0.64	0.00	0.00	0.34	0.08	0.51
	d_{x2-y2}	0.00	0.13	**1.26**	0.00	0.01	0.01
	d_{xy}	0.00	0.74	0.09	0.06	0.08	0.08

Table S13. Absolute values of the hopping integrals between *d* orbitals of Cr_1 and Cr_3 atoms and *p* orbitals of $S_{1,3}$ and $S'_{1,3}$ for 5% tensile strain along *a*. Hopping between orbitals involved in the most relevant magnetic superexchange channels for J_2 are highlighted in bold.

		$S_{1,3}$ (p_z)	$S_{1,3}$ (p_x)	$S_{1,3}$ (p_y)	$S'_{1,3}$ (p_z)	$S'_{1,3}$ (p_x)	$S'_{1,3}$ (p_y)
**Cr_1**	**d_{z2}**	**1.14**	0.31	0.31	0.00	0.10	**0.69**
	**d_{xz}**	0.33	**0.51**	0.07	0.07	0.00	0.00
	**d_{yz}**	0.33	0.07	**0.51**	0.48	0.00	0.00
	**d_{x2-y2}**	0.00	0.01	0.01	0.00	0.16	1.04
	**d_{xy}**	0.09	0.07	0.07	0.00	0.54	0.17
**Cr_3**	**d_{z2}**	0.00	0.10	**0.69**	**1.14**	0.31	0.31
	**d_{xz}**	0.07	0.00	0.00	0.33	0.51	0.07
	**d_{yz}**	0.48	0.00	0.00	0.33	0.07	0.51
	**d_{x2-y2}**	0.00	0.16	**1.04**	0.00	0.01	0.01
	**d_{xy}**	0.00	0.54	0.17	0.09	0.07	0.07

Table S14. Absolute values of the hopping integrals between *d* orbitals of Cr_1 and Cr_3 atoms and *p* orbitals of $S_{1,3}$ and $S'_{1,3}$ for 5% compressive strain along *b*. Hopping between orbitals involved in the most relevant magnetic superexchange channels for J_2 are highlighted in bold.

		$S_{1,3}$ (p_z)	$S_{1,3}$ (p_x)	$S_{1,3}$ (p_y)	$S'_{1,3}$ (p_z)	$S'_{1,3}$ (p_x)	$S'_{1,3}$ (p_y)
Cr_1	d_{z2}	**1.29**	0.37	0.37	0.00	0.11	**0.83**
	d_{xz}	0.41	**0.63**	0.10	0.08	0.00	0.00
	d_{yz}	0.41	0.10	**0.63**	0.53	0.00	0.00
	d_{x2-y2}	0.00	0.02	0.02	0.00	0.14	1.16
	d_{xy}	0.10	0.10	0.10	0.00	0.64	0.14
Cr_3	d_{z2}	0.00	0.11	**0.83**	**1.29**	0.37	0.37
	d_{xz}	0.08	0.00	0.00	0.41	0.63	0.10
	d_{yz}	0.53	0.00	0.00	0.41	0.10	0.63
	d_{x2-y2}	0.00	0.14	**1.16**	0.00	0.02	0.02
	d_{xy}	0.00	0.64	0.14	0.10	0.10	0.10

Table S15. Absolute values of the hopping integrals between *d* orbitals of Cr_1 and Cr_3 atoms and *p* orbitals of $S_{1,3}$ and $S'_{1,3}$ for 5% tensile strain along *b*. Hopping between orbitals involved in the most relevant magnetic superexchange channels for J_2 are highlighted in bold.

		$S_{1,3}$ (p_z)	$S_{1,3}$ (p_x)	$S_{1,3}$ (p_y)	$S'_{1,3}$ (p_z)	$S'_{1,3}$ (p_x)	$S'_{1,3}$ (p_y)
Cr_1	d_{z2}	**1.08**	0.27	0.27	0.00	0.07	**0.61**
	d_{xz}	0.27	**0.41**	0.06	0.06	0.00	0.00
	d_{yz}	0.27	0.06	**0.41**	0.57	0.00	0.00
	d_{x2-y2}	0.00	0.01	0.01	0.00	0.15	1.13
	d_{xy}	0.06	0.06	0.06	0.00	0.63	0.13
Cr_3	d_{z2}	0.00	0.07	**0.61**	**1.08**	0.27	0.27
	d_{xz}	0.06	0.00	0.00	0.27	0.41	0.06
	d_{yz}	0.57	0.00	0.00	0.27	0.06	0.41
	d_{x2-y2}	0.00	0.15	**1.13**	0.00	0.01	0.01
	d_{xy}	0.00	0.63	0.13	0.06	0.06	0.06

Table S16. Absolute values of the hopping integrals between *d* orbitals of Cr_1 and Cr_4 atoms and *p* orbitals of $S_{1,4}$. Hopping between orbitals involved in the most relevant magnetic superexchange channels for J_3 are highlighted in bold.

		$S_{1,4}$ (p_z)	$S_{1,4}$ (p_x)	$S_{1,4}$ (p_y)
Cr_1	d_{z2}	**1.18**	0.31	0.31
	d_{xz}	**0.33**	**0.51**	0.08
	d_{yz}	0.33	0.08	0.51
	d_{x2-y2}	0.00	0.00	0.00
	d_{xy}	0.08	0.07	0.07
Cr_4	d_{z2}	**1.18**	**0.31**	0.31
	d_{xz}	0.33	0.51	0.08
	d_{yz}	0.33	0.08	0.51
	d_{x2-y2}	0.00	0.00	0.00
	d_{xy}	0.08	0.07	0.07

Table S17. Absolute values of the hopping integrals between *d* orbitals of Cr_1 and Cr_4 atoms and *p* orbitals of $S_{1,4}$ for 5% compressive strain along *a*. Hopping between orbitals involved in the most relevant magnetic superexchange channels for J_3 are highlighted in bold.

		$S_{1,4}$ (p_z)	$S_{1,4}$ (p_x)	$S_{1,4}$ (p_y)
Cr_1	d_{z2}	**1.23**	0.32	0.32
	d_{xz}	**0.34**	**0.51**	0.08
	d_{yz}	0.34	0.08	0.51
	d_{x2-y2}	0.00	0.01	0.01
	d_{xy}	0.06	0.08	0.08
Cr_4	d_{z2}	**1.23**	0.32	0.32
	d_{xz}	**0.34**	**0.51**	0.08
	d_{yz}	0.34	0.08	0.51
	d_{x2-y2}	0.00	0.01	0.01
	d_{xy}	0.06	0.08	0.08

Table S18. Absolute values of the hopping integrals between *d* orbitals of Cr_1 and Cr_4 atoms and *p* orbitals of $S_{1,4}$ for 5% tensile strain along *a*. Hopping between orbitals involved in the most relevant magnetic superexchange channels for J_3 are highlighted in bold.

		$S_{1,4}$ (p_z)	$S_{1,4}$ (p_x)	$S_{1,4}$ (p_y)
Cr_1	d_{z2}	**1.14**	0.31	0.31
	d_{xz}	**0.33**	**0.51**	0.07
	d_{yz}	0.33	0.07	0.51
	d_{x2-y2}	0.00	0.01	0.01
	d_{xy}	0.09	0.07	0.07
Cr_4	d_{z2}	**1.14**	0.31	0.31
	d_{xz}	**0.33**	**0.51**	0.07
	d_{yz}	0.33	0.07	0.51
	d_{x2-y2}	0.00	0.01	0.01
	d_{xy}	0.09	0.07	0.07

Table S19. Absolute values of the hopping integrals between *d* orbitals of Cr_1 and Cr_4 atoms and *p* orbitals of $S_{1,4}$ for 5% compressive strain along *b*. Hopping between orbitals involved in the most relevant magnetic superexchange channels for J_3 are highlighted in bold.

		$S_{1,4}$ (p_z)	$S_{1,4}$ (p_x)	$S_{1,4}$ (p_y)
Cr_1	d_{z2}	**1.29**	0.37	0.37
	d_{xz}	**0.41**	**0.63**	0.10
	d_{yz}	0.41	0.10	0.63
	d_{x2-y2}	0.00	0.01	0.01
	d_{xy}	0.10	0.10	0.10
Cr_4	d_{z2}	**1.29**	0.37	0.37
	d_{xz}	**0.41**	**0.63**	0.10
	d_{yz}	0.41	0.10	0.63
	d_{x2-y2}	0.00	0.01	0.01
	d_{xy}	0.10	0.10	0.10

Table S20. Absolute values of the hopping integrals between *d* orbitals of Cr_1 and Cr_4 atoms and *p* orbitals of $S_{1,4}$ for 5% tensile strain along *b*. Hopping between orbitals involved in the most relevant magnetic superexchange channels for J_3 are highlighted in bold.

		$S_{1,4}$ (p_z)	$S_{1,4}$ (p_x)	$S_{1,4}$ (p_y)
Cr_1	d_{z2}	**1.07**	0.27	0.27
	d_{xz}	**0.27**	**0.41**	0.06
	d_{yz}	0.27	0.06	0.41
	d_{x2-y2}	0.00	0.01	0.01
	d_{xy}	0.06	0.06	0.06
Cr_4	d_{z2}	**1.07**	0.27	0.27
	d_{xz}	**0.27**	**0.41**	0.06
	d_{yz}	0.27	0.06	0.41
	d_{x2-y2}	0.00	0.01	0.01
	d_{xy}	0.06	0.06	0.06

5. Phase diagram of the exchange Hamiltonian.

In order to rationalize problematic regions in the Curie temperature and group velocity calculations we plotted phase diagram (Figure S43a) of exchange Hamiltonian (eq. 1). In Figure S43b four possible ground states for this Hamiltonian are presented. Note that for each AFM state a "pair" state exists, which is totally equivalent to the one provided in the Figure S43b (for example AFM-ab "pair" state will be the one where spin-up and spin-down lines are aligned along different diagonal in the right bottom picture in Figure S43b).

We mapped the phase diagram (Figures S43c, d) to the regions of strain and Hubbard U discussed in the main text using fit for exchange parameters (eq. 1 in the main text).

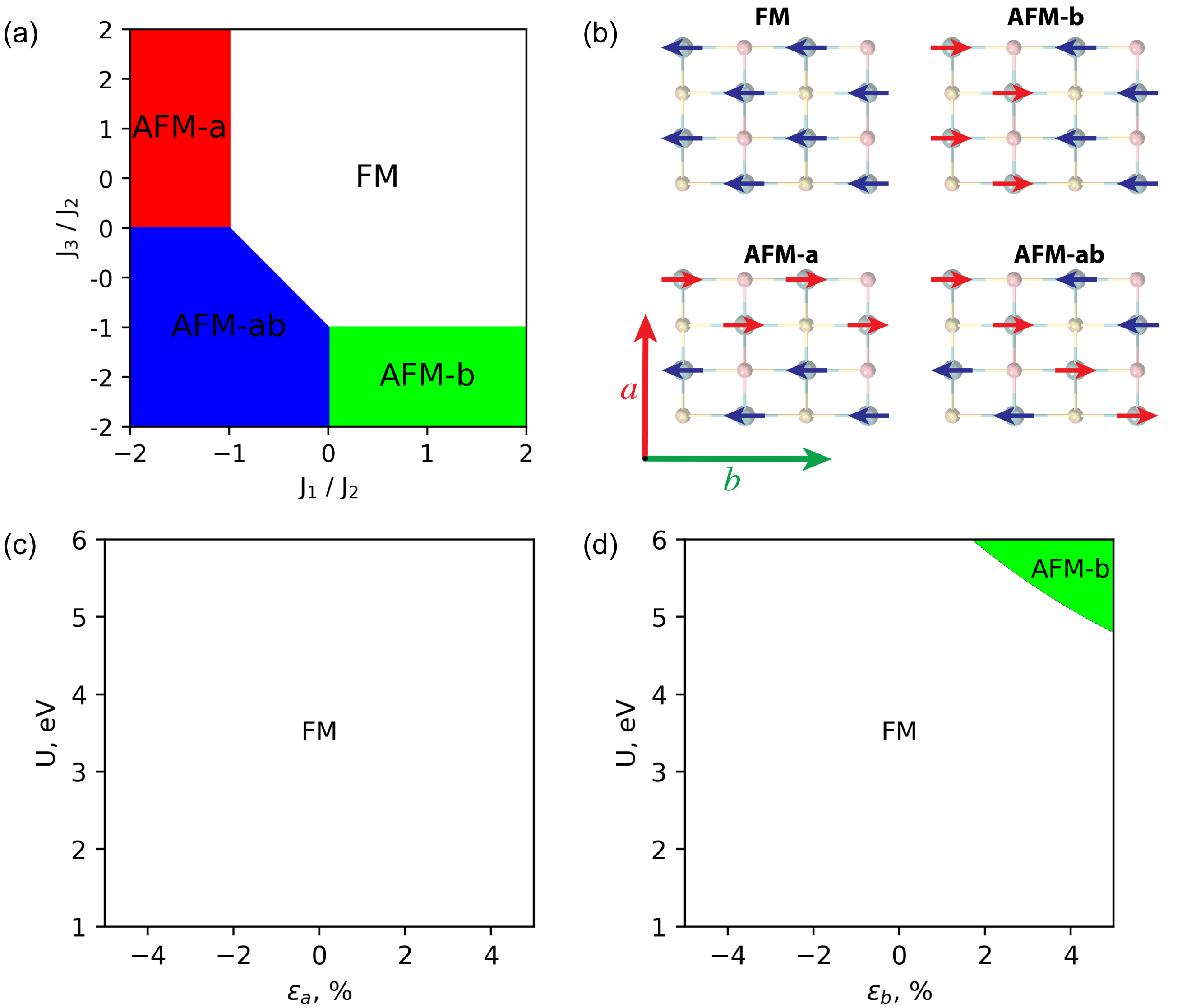

Figure S43. (a) Phase diagram of the exchange Hamiltonian (eq. 1). (b) Four spin structures corresponding to possible ground states. Phase diagram in the coordinates of strain and Hubbard U for (c) strain along *a* axis, (d) strain along *b* axis.

6. Magnon dispersion and Curie temperature

Starting from exchange Hamiltonian (eq. 1) we obtained spin-wave Hamiltonian through Holstein-Primakoff transformation (eqs. 2, 3 in the main text), where structural factors for first, second and third order neighbors have the following form (x axis is along *a* crystallographic axis, y axis is along *b* crystallographic axis):

$$\gamma_k^{(1)} = \cos(\mathrm{k_x a})$$

$$\gamma_k^{(2)} = \cos\left(\frac{k_x a}{2}\right)\cos\left(\frac{k_y b}{2}\right) \tag{2}$$

$$\gamma_k^{(3)} = \cos(\mathrm{k}_y b)$$

Obtained magnon dispersion relations were used to calculate Curie temperature by solving equation 3 self-consistently.

$$M(T) = S - \frac{1}{2(2\pi)^2}\int_{\mathrm{BZ}} \frac{\mathrm{d}^2\vec{k}}{\mathrm{e}^{\frac{M(T)E(\vec{k})}{Sk_bT}} - 1} = S - \frac{1}{2N_{k_x}N_{k_y}}\sum_{\vec{k}} \frac{1}{\mathrm{e}^{\frac{M(T)E(\vec{k})}{Sk_bT}} - 1} \tag{3}$$

where S = 3/2 - is a spin of magnetic Cr atom, M(T) is associated with it magnetic moment. Numerical integration was performed at 300×300 k-grid with temperature step of 0.1 K. Curie temperature (T_C) was defined as a temperature at which $M(T_C) = M_0/2$.

Anisotropy gap, which is presented in Figures 4b, e in the main text, has the following form:

$$\Delta = 2S\sum_{n=1}^{3}(J_n^z n_n) + 2SD \tag{4}$$

In Figures S44, S45 magnon dispersion variation under two types of applied strain for 6 values of Hubbard U is presented. K-path notation is the same as in [6].

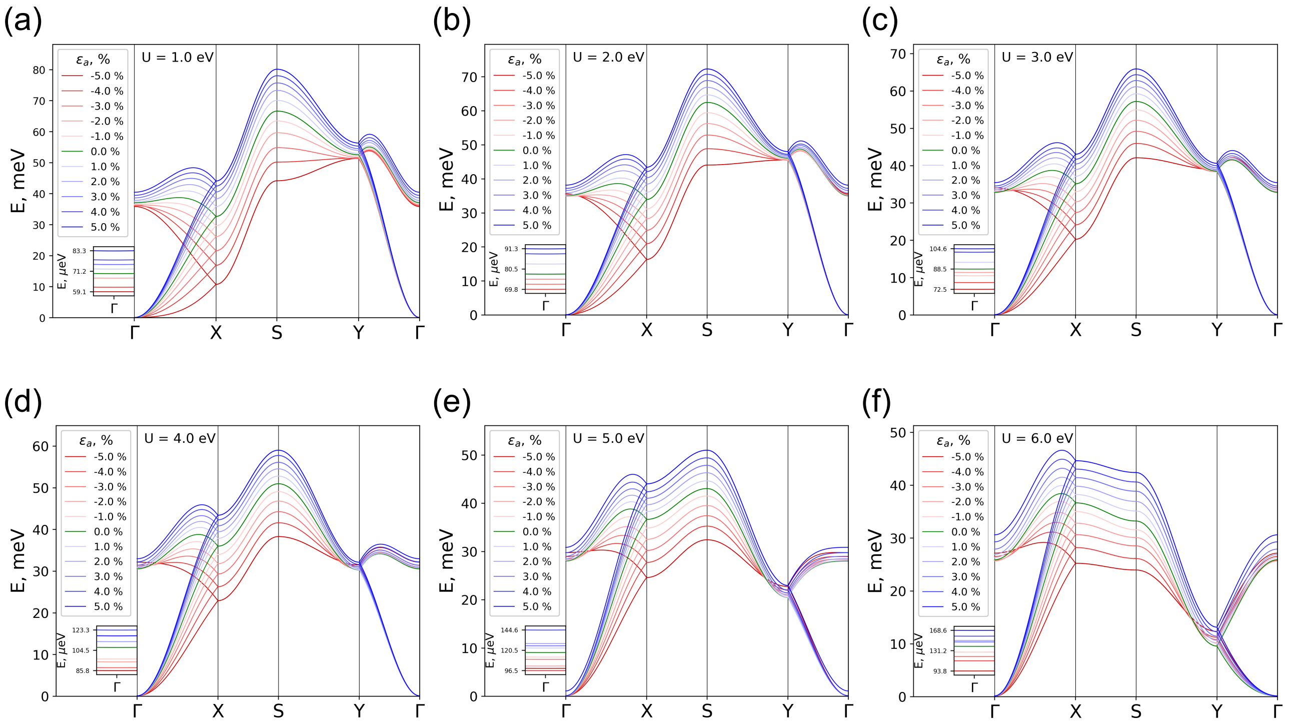

Figure S44. Magnon dispersion for Hubbard U 1-6 eV for (a-f) for uniaxial strain along a. Values of strain and Hubbard U are specified in the pictures.

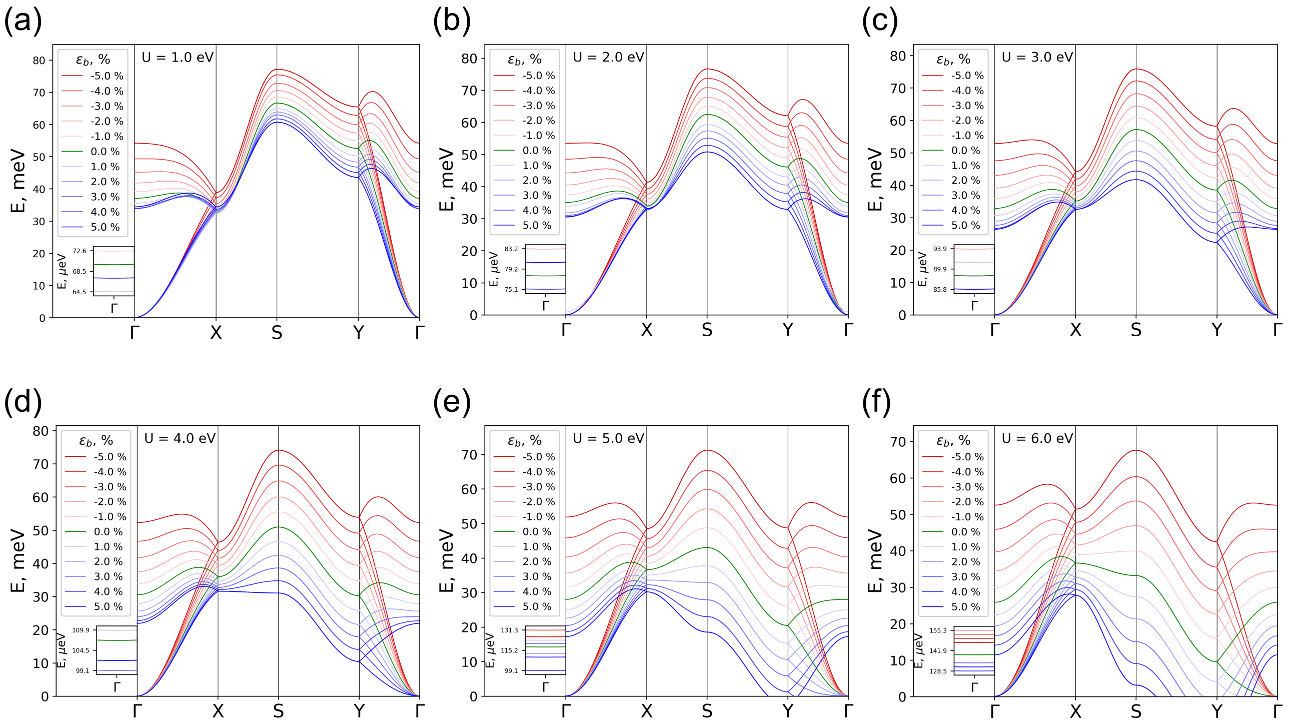

Figure S45. Magnon dispersion for Hubbard U 1-6 eV for (a-f) for uniaxial strain along b. Values of strain and Hubbard U are specified in the pictures.

7. LLG-driven atomistic spin dynamic simulation

We simulated dynamic behavior of spin waves in the monolayer sample with unit cell replicated 541 times along a and 401 times along b, which corresponds to the dimensions of the unstrained sample of 190 nm × 190 nm. Spin wave were induced by application of an input magnetic field oscillating along the direction of c crystallographic axis

$$B = B_0 \sin(2\pi\nu t) \tag{5}$$

where B_0 = 0.13 T, ν = 1 THz. Duration of the input field is set to 1 ps (unless the frequency/time correction is introduced, see below). In Figure S47 the snapshots of that simulations are presented. (x axis is along a, y - along b, z axis - along c). Note that with that input magnetic field's frequency only the magnons with k-vectors around Γ-point are exited.

Group velocity was calculated by tracking the coordinate of the spin deviation maximum along two directions (x and y) with time and fitting obtained dependency (example of this dependency is provided in Fig. S46)

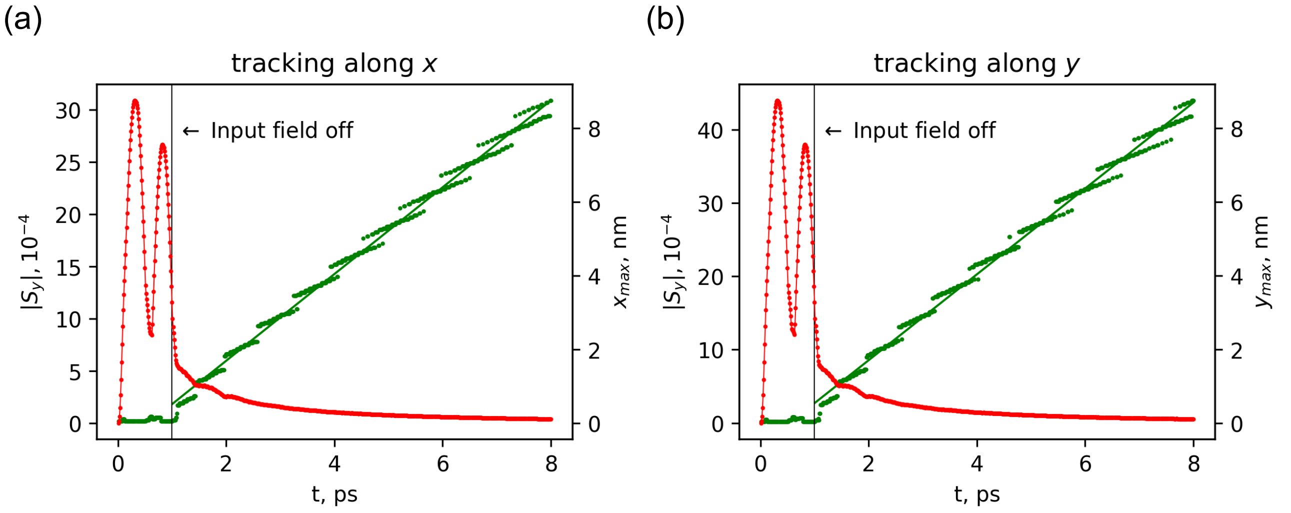

Figure S46. Example of data obtained from dynamic simulations for U = 3 eV, ε = 0%. (a) tracking along x, (b) tracking along y. Red line corresponds to the absolute value of spin deviation along y, Green line is a fit for the x_{max} and y_{max}.

7.1 Snapshots of spin dynamics simulations

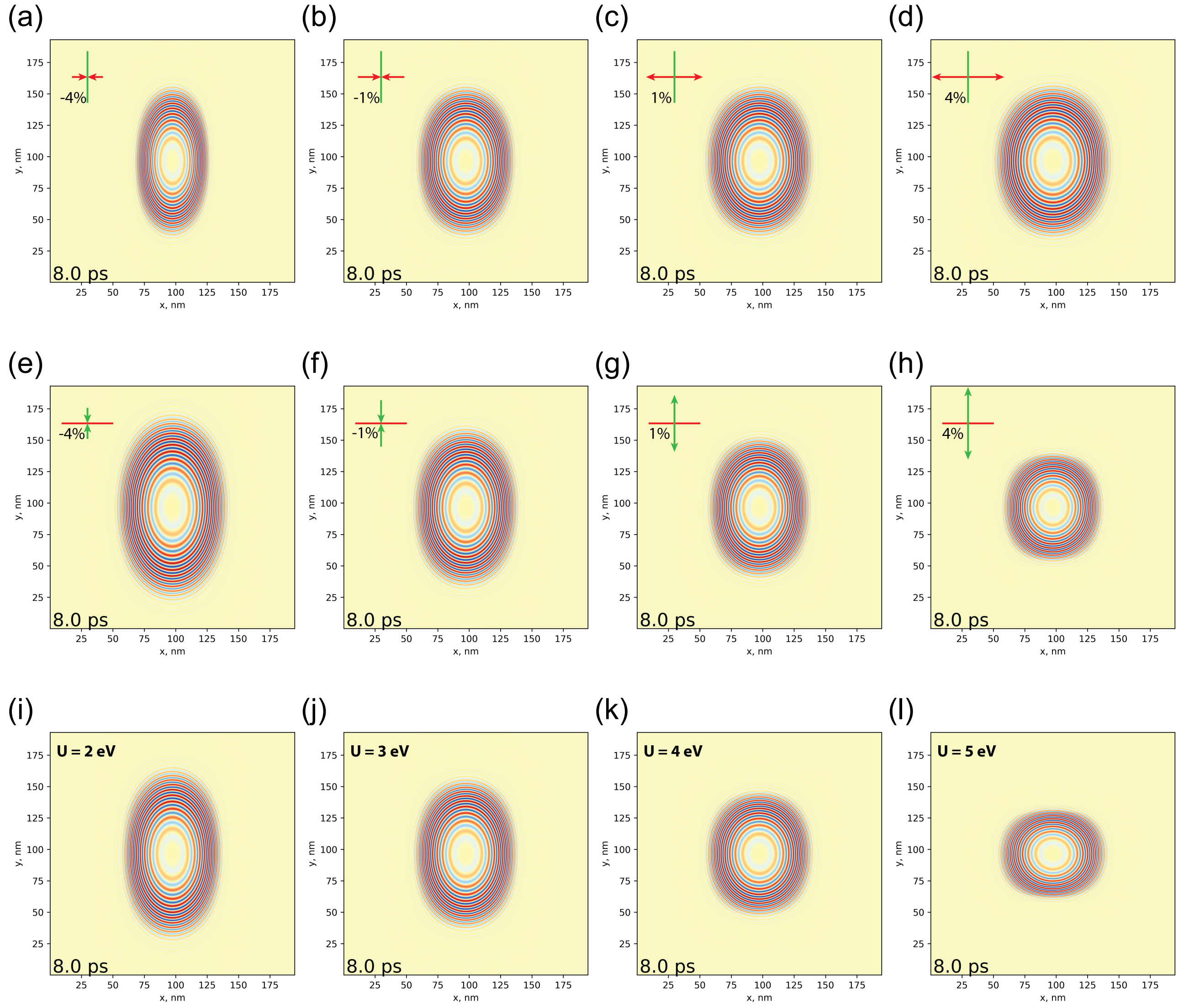

Figure S47. Snapshots of dynamics simulations for uniaxial strain (a-d) along *a* axis, (e-h) along *b* axis and (i-l) with several values of Hubbard U. Without frequency/time correction.

7.2 Group velocity

The results of dynamic simulation where directions of group velocity and strain are perpendicular to each other are presented in Figure S48. Those results coexist with the magnon dispersion from Figures S44 and S45 since strain along *a* keep the slope of acoustic branch of dispersion along Y-Γ almost unchanged, while for the case of strain along *b* the slope of acoustic branch of dispersion along X-Γ is changing (see also Figures S52, S53).

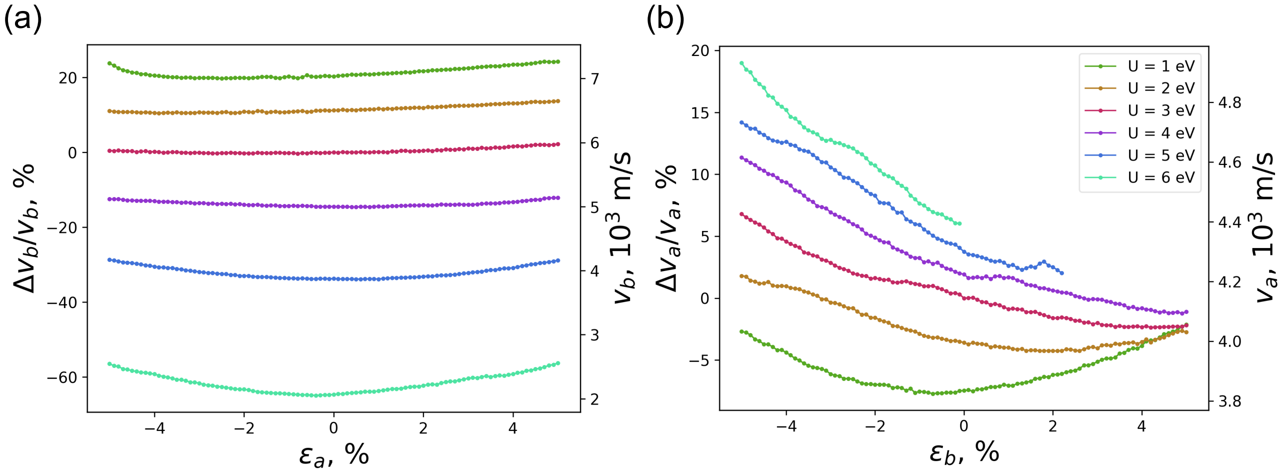

Figure S48. (a) Group velocity along *b* with strain applied along *a* and (b) group velocity along *a* with strain applied along *b*. Calculations without frequency/time correction.

7.3 Group velocity with frequency/time correction

As was mentioned in the main text group velocities undergo several abrupt changes, which we attributed to the deactivation of magnon modes. Let us investigate the case of v_a and ε_a, where we will focus on the Γ-X path of magnon dispersion and U = 3 eV (Figures 3a, 5a in the main text).

Because of the sample's boundary conditions the wave vector k_x assumes N discrete values. Thus the eigenstates of the SW Hamiltonian can be represented as

$$|n_1, n_2, \ldots, n_N\rangle \tag{6}$$

The frequency of perturbating magnetic field is kept unchanged with strain, so upon compressing of the monolayer the number of exited magnon modes increases (because magnon dispersion curve decreases), while the group velocity of each mode decreases. Thus, lets assume that at some value of strain ε_0 (0% in Figure 5a of the main text, U = 3 eV) the modes n_1, ...n_i are exited, then with further compressing the group velocity of each mode n_1, ...n_i will decrease gradually (see Figure S51c) until around $\varepsilon_1 < \varepsilon_0$ (-0.5% in Figure 5a of the main text, U = 3 eV). At this point the energy of the mode n_i is not low enough for the perturbating magnetic field to excite it, therefore step-like decrease (around -0.5% in Figure 5a of the main text, U = 3 eV) will occur since the mode n_i is deactivated. Hereafter, the group velocity of each mode n_1, ..., n_{i-1} will further decrease gradually until it will not be possible to excite the mode n_{i-1} (-2% in Figure 5a of the main text, U = 3 eV).

Since we have the information about evolution of the magnon dispersion with strain one can introduce the correction for the frequency and duration of input magnetic field (in a way that frequency is following the dispersion and product νt is conserved). Indeed, we calculated that correction (Figure S50), where for U = 3 eV and ε = 0%: ν = 1 THz and t = 1 ps. For each combination of strain and group velocity direction we defined the k-vectors (k_x or k_y) which corresponds to the reference point (ε = 0%, U = 3 eV) and then modified the frequency of the input field with respect to the change of magnon frequency for this k-vector. We calculated group velocities from dynamic simulations with frequency/time correction introduced (Figure S49). and found that group velocities now are following smooth trends, which basically mirroring the trends for frequency corrections (Figure S50) as expected.

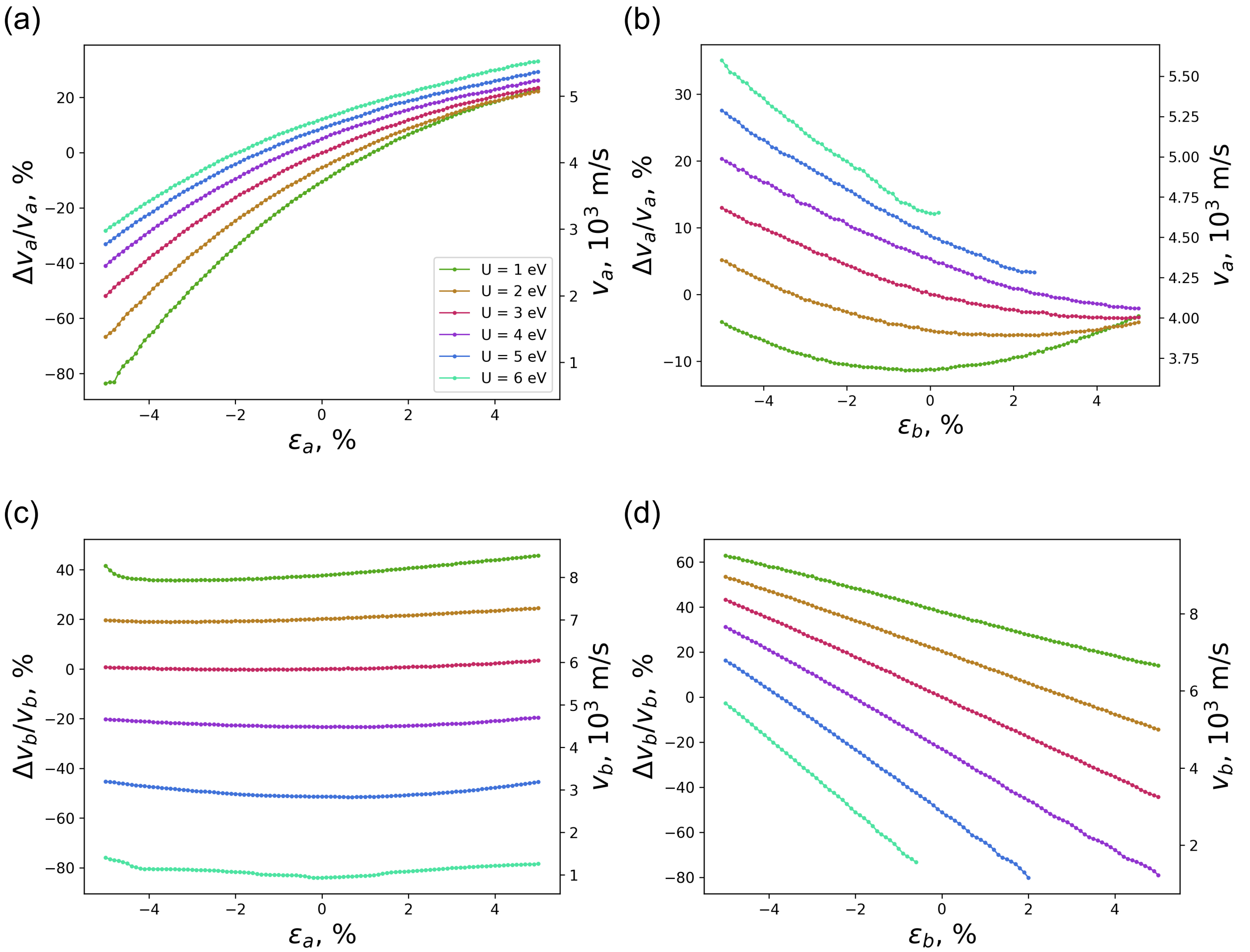

Figure S49. Group velocities with frequency and time correction. With strain applied along (a, c) a and (b, d) b. Velocity in the direction of (a, b) a and (c, d) b.

7.4 Correction of frequency

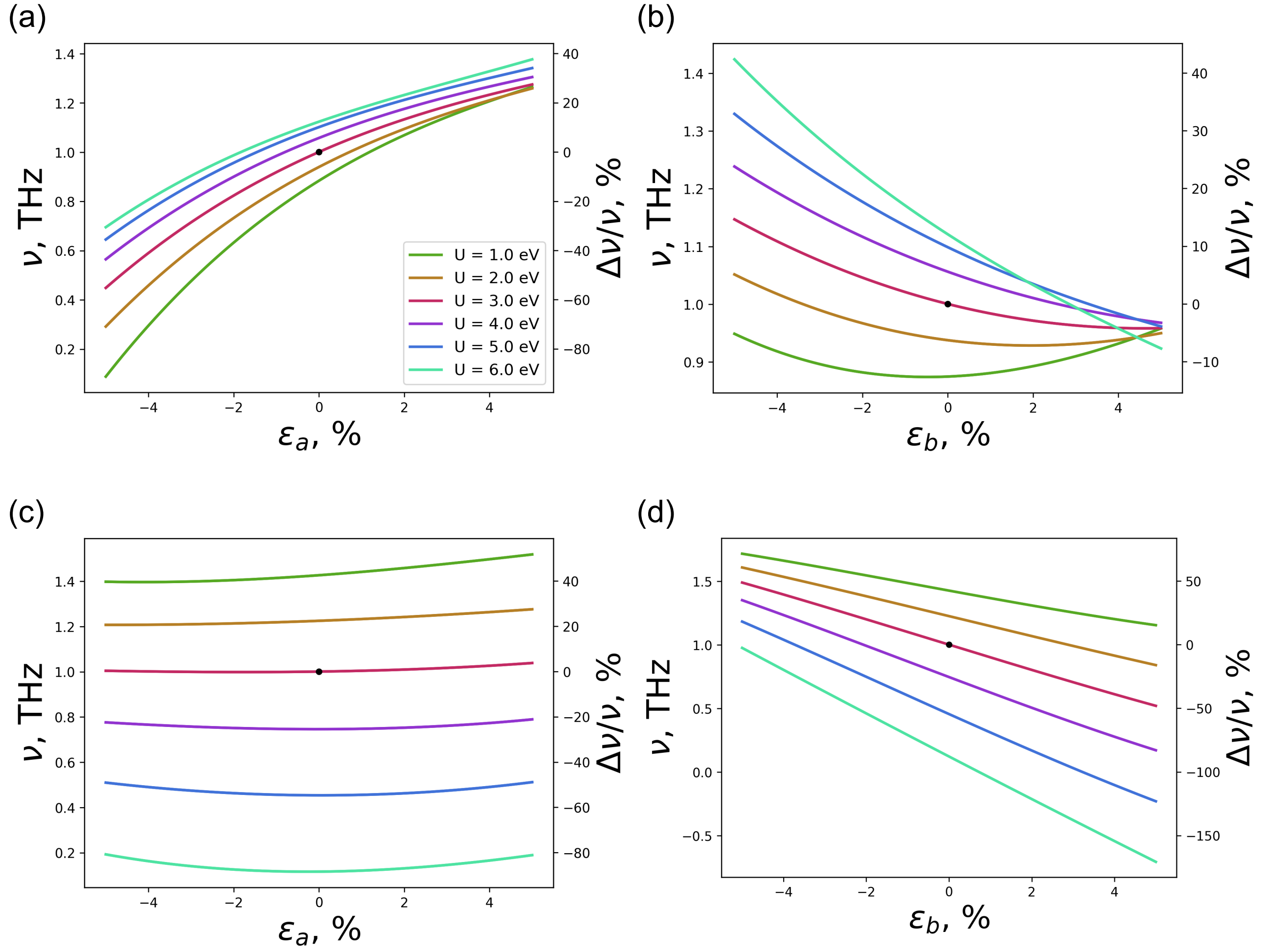

Figure S50. Frequency correction for the dynamic simulation. Reference point is chosen to be at ε = 0%, U = 3 eV with ν = 1 THz, t = 1ps (black point in the pictures). (a, b) Correction for calculating v_a with strain applied along (a) *a* (b) *b*. (c, d) Correction for calculating v_b with strain applied along (c) *a*, (d) *b*.

7.5 Group velocities vs momentum within Brillouin zone

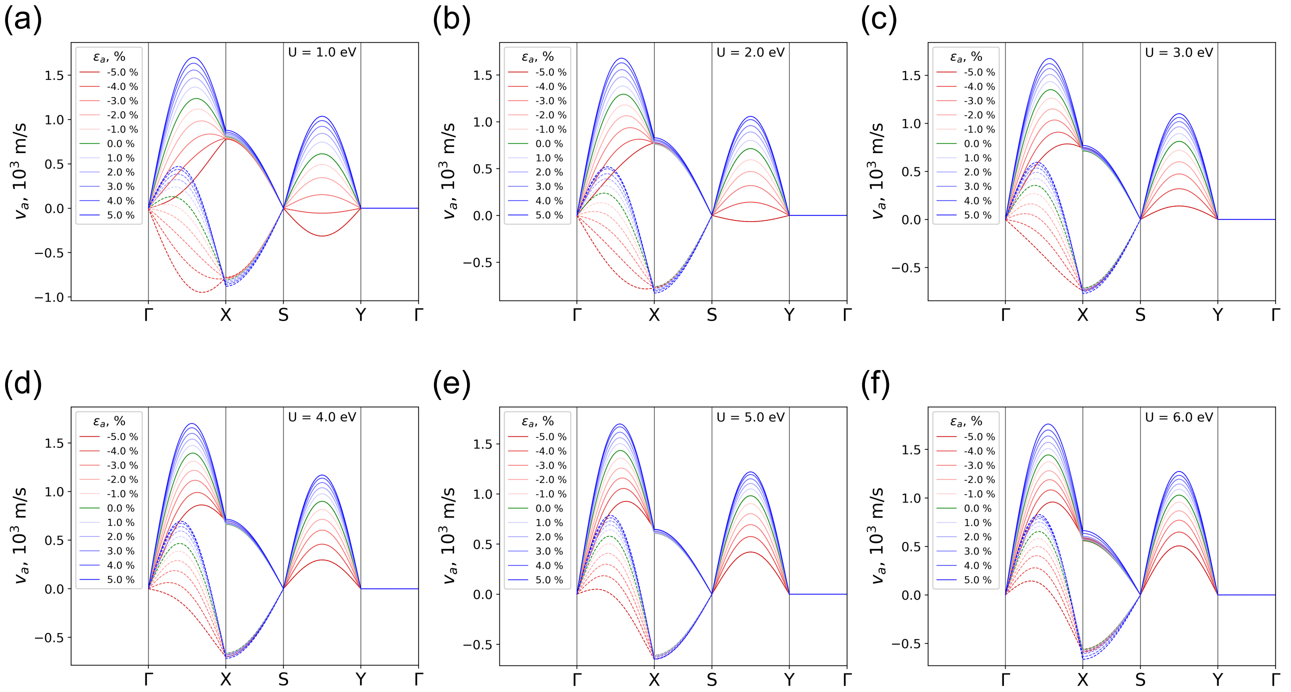

Figure S51. Group velocity v_a as a function of momentum for Hubbard U 1-6 eV (a-f) for uniaxial strain along a. Values of strain and Hubbard U are specified in the pictures. Solid lines - acoustic mode (in-phase), dashed lines - optical modes (out-of-phase).

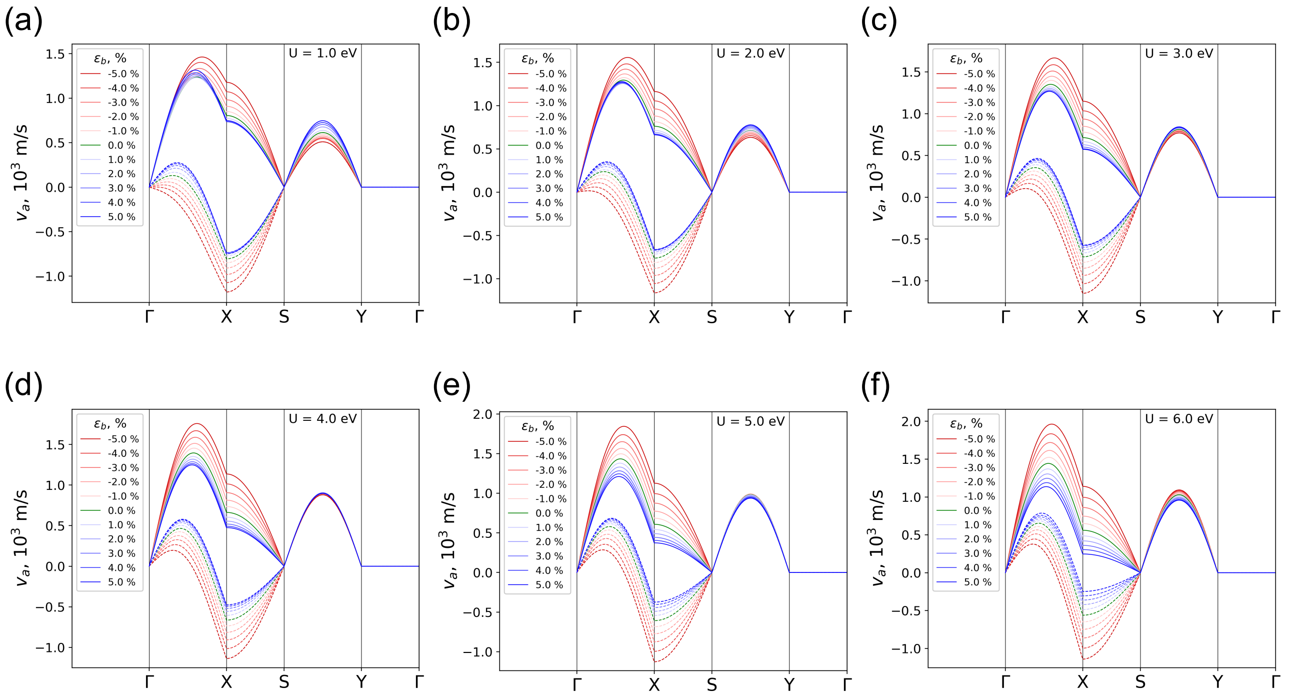

Figure S52. Group velocity v_a as a function of momentum for Hubbard U 1-6 eV (a-f) for uniaxial strain along b. Values of strain and Hubbard U are specified in the pictures. Solid lines - acoustic mode (in-phase), dashed lines - optical modes (out-of-phase).

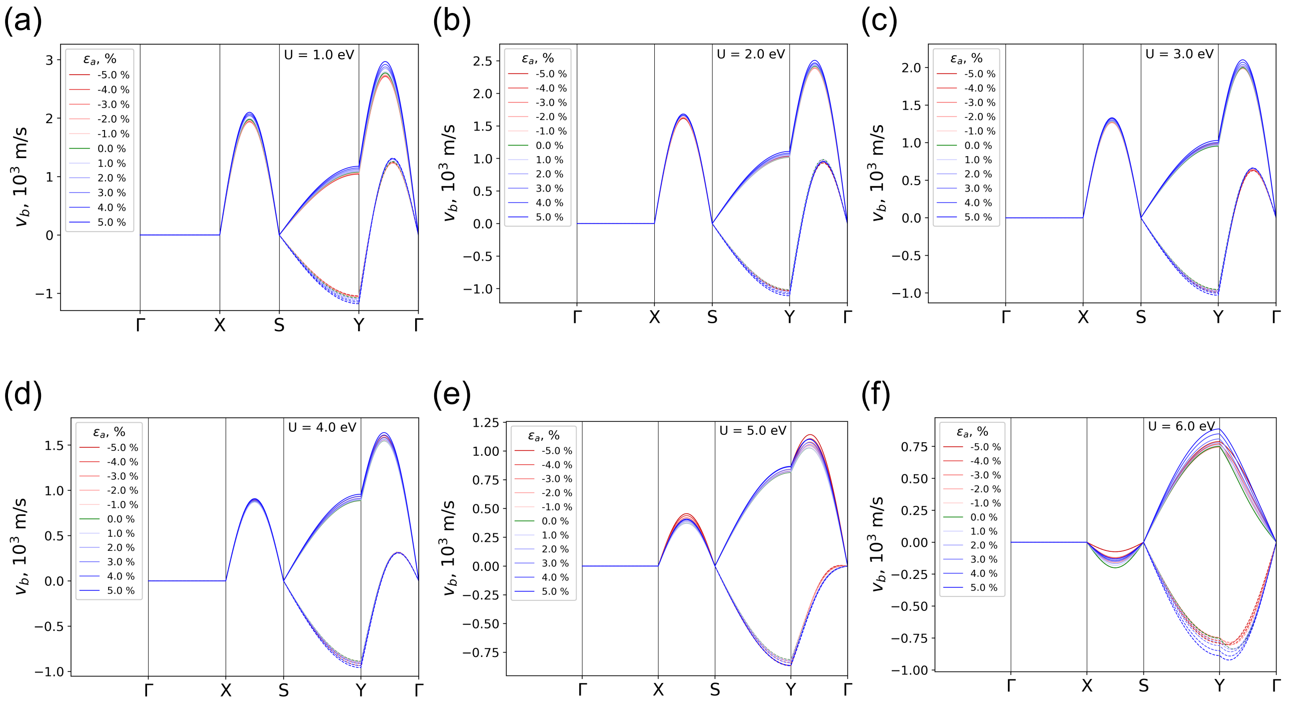

Figure S53. Group velocity v_b as a function of momentum for Hubbard U 1-6 eV (a-f) for uniaxial strain along a. Values of strain and Hubbard U are specified in the pictures. Solid lines - acoustic mode (in-phase), dashed lines - optical modes (out-of-phase).

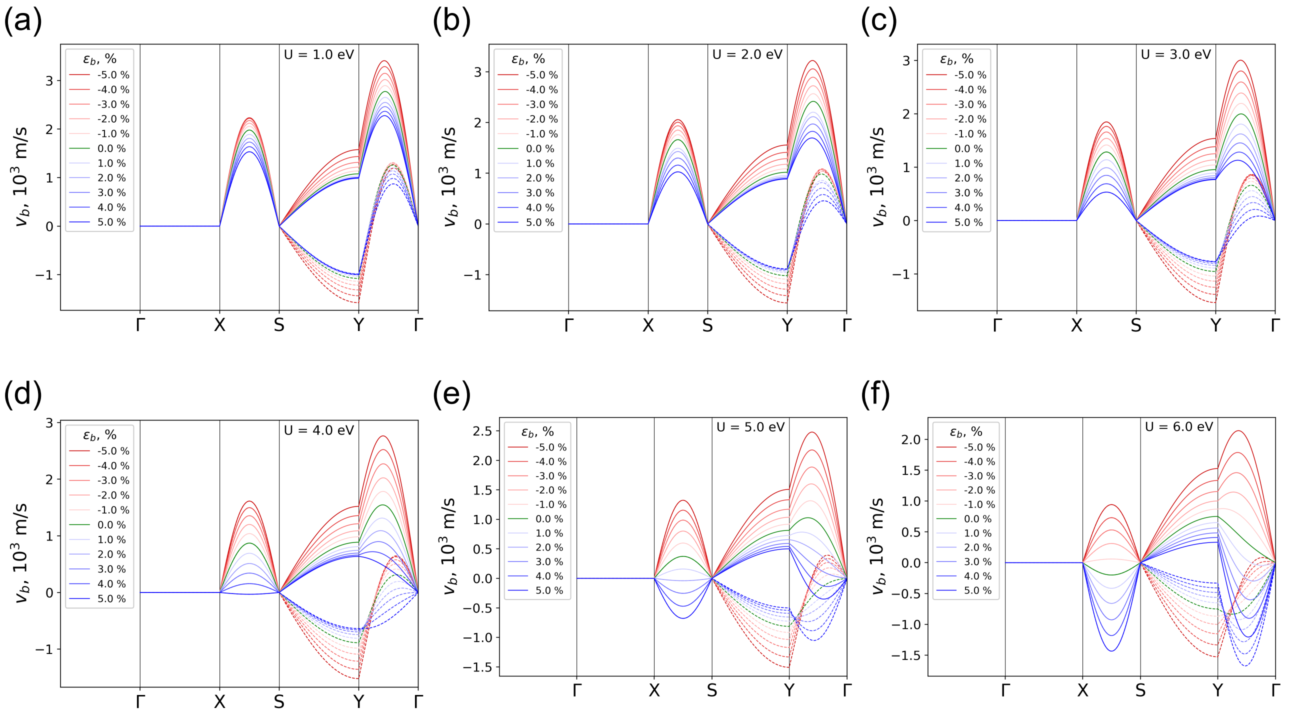

Figure S54. Group velocity v_b as a function of momentum for Hubbard U 1-6 eV (a-f) for uniaxial strain along b. Values of strain and Hubbard U are specified in the pictures. Solid lines - acoustic mode (in-phase), dashed lines - optical modes (out-of-phase).

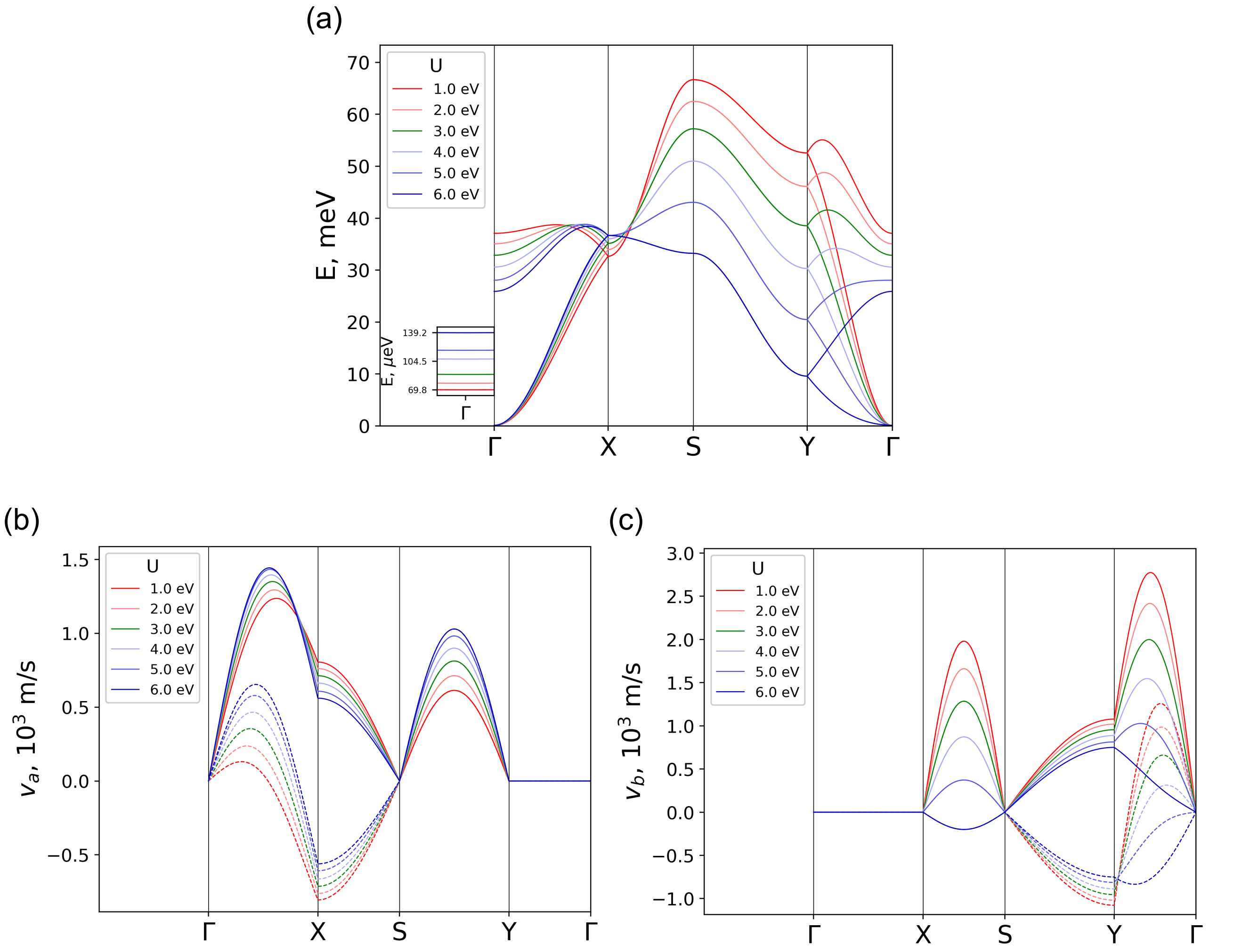

Figure S55. (a) Magnon dispersion, (b) group velocity along *a*, (c) group velocity along *b* for ε = 0% under the variation of Hubbard U. Values Hubbard U are specified in the pictures.

8. Evolution of self-consistent Hubbard U with strain

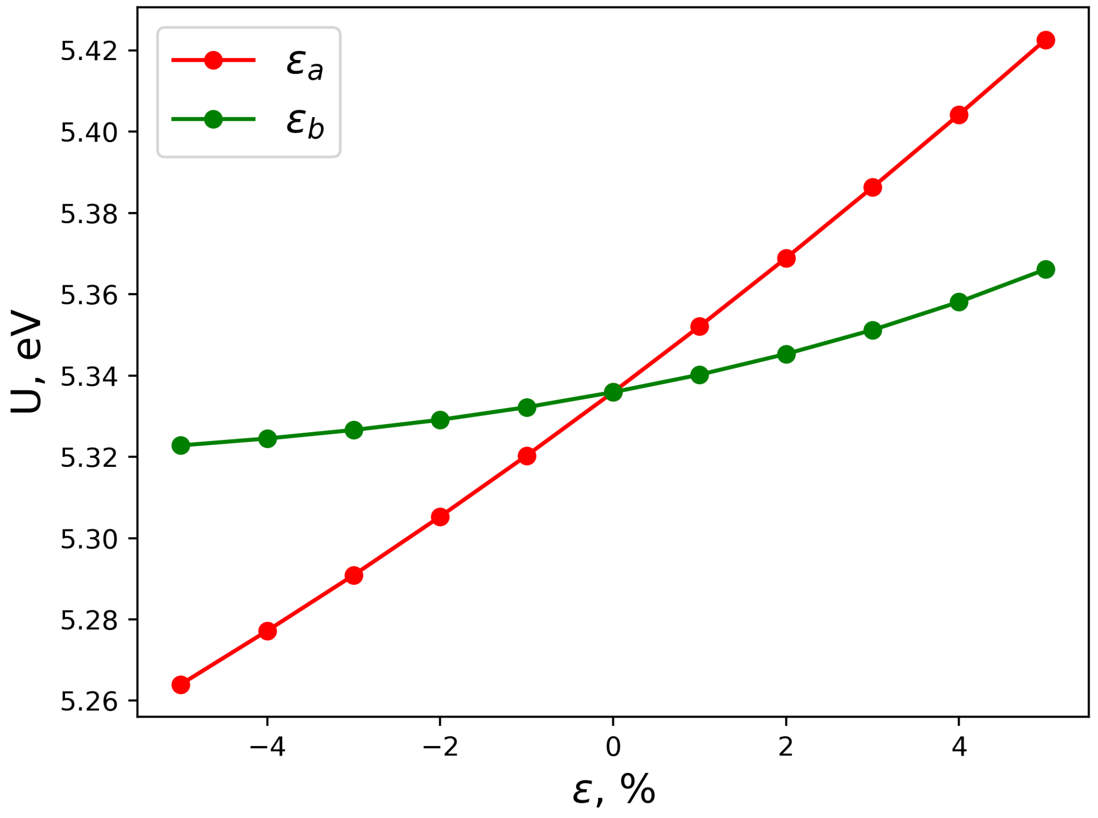

Figure S56. Evolution of self-consistent Hubbard U determined by density functional perturbation theory in the hp.x code of the Quantum Espresso package with strain along *a* axis (red) and *b* axis (green).

(1) Guo, Y.; Zhang, Y.; Yuan, S.; Wang, B.; Wang, J. Chromium Sulfide Halide Monolayers: Intrinsic Ferromagnetic Semiconductors with Large Spin Polarization and High Carrier Mobility. *Nanoscale* **2018**, *10* (37), 18036–18042.

(2) Xu, X.; Wang, X.; Chang, P.; Chen, X.; Guan, L.; Tao, J. Strong Spin-Phonon Coupling in Two-Dimensional Magnetic Semiconductor CrSBr. *The Journal of Physical Chemistry C* **2022**, *126* (25), 10574–10583.

(3) Yang, K.; Wang, G.; Liu, L.; Lu, D.; Wu, H. Triaxial Magnetic Anisotropy in the Two-Dimensional Ferromagnetic Semiconductor CrSBr. *Physical Review B* **2021**, *104* (14), 144416.

(4) Wang, H.; Qi, J.; Qian, X. Electrically Tunable High Curie Temperature Two-Dimensional Ferromagnetism in van Der Waals Layered Crystals. *Applied Physics Letters* **2020**, *117* (8), 083102.

(5) Scheie, A.; Ziebel, M.; Chica, D. G.; Bae, Y. J.; Wang, X.; Kolesnikov, A. I.; Zhu, X.; Roy, X. Spin Waves and Magnetic Exchange Hamiltonian in CrSBr. Adv. Sci. **2022**, 2202467.

(6) Setyawan W.; Curtarolo S. High-throughput electronic band structure calculations: Challenges and tools. *Computational materials science* **2010,** 49(2), 299-312.
